# Simultaneous mass spectrometry analysis of cisplatin with oligonucleotide-peptide mixtures: implications for the mechanism of action

**DOI:** 10.1007/s00775-022-01924-9

**Published:** 2022-01-22

**Authors:** Farangis Mansouri, Luc Patiny, Daniel Ortiz, Laure Menin, Curtis A. Davey, Fakhrossadat Mohammadi, Paul J. Dyson

**Affiliations:** 1 Institute of Chemical Sciences and Engineering, Swiss Federal Institute of Technology Lausanne (EPFL), 1015 Lausanne, Switzerland; 2 Department of Chemistry Institute for Advanced Studies in Basic Sciences (IASBS), 444 Prof. Sobouti Blvd., Gava Zang, 45137-66731 Zanjan, Iran; 3 School of Biological Sciences, Nanyang Technological University, 60 Nanyang Drive, Singapore, 637551 Singapore; 4 NTU Institute of Structural Biology, Nanyang Technological University, 59 Nanyang Drive, Singapore, 636921 Singapore

**Keywords:** Cisplatin, Mass spectrometry, Drug targets, Histone core particle

## Abstract

Although genomic DNA is the primary target of anticancer platinum-based drugs, interactions with proteins also play a significant role in their overall activity. In this study, competitive binding of cisplatin with an oligonucleotide and two peptides corresponding to segments of H2A and H2B histone proteins was investigated by mass spectrometry. Following the determination of the cisplatin binding sites on the oligonucleotide and peptides by tandem mass spectrometry, competitive binding was studied and transfer of platinum fragments from the platinated peptides to the oligonucleotide explored. In conjunction with previous studies on the nucleosome, the results suggest that all four of the abundant histone proteins serve as a platinum drug reservoir in the cell nucleus, providing an adduct pool that can be ultimately transferred to the DNA.

**Graphical abstract:**

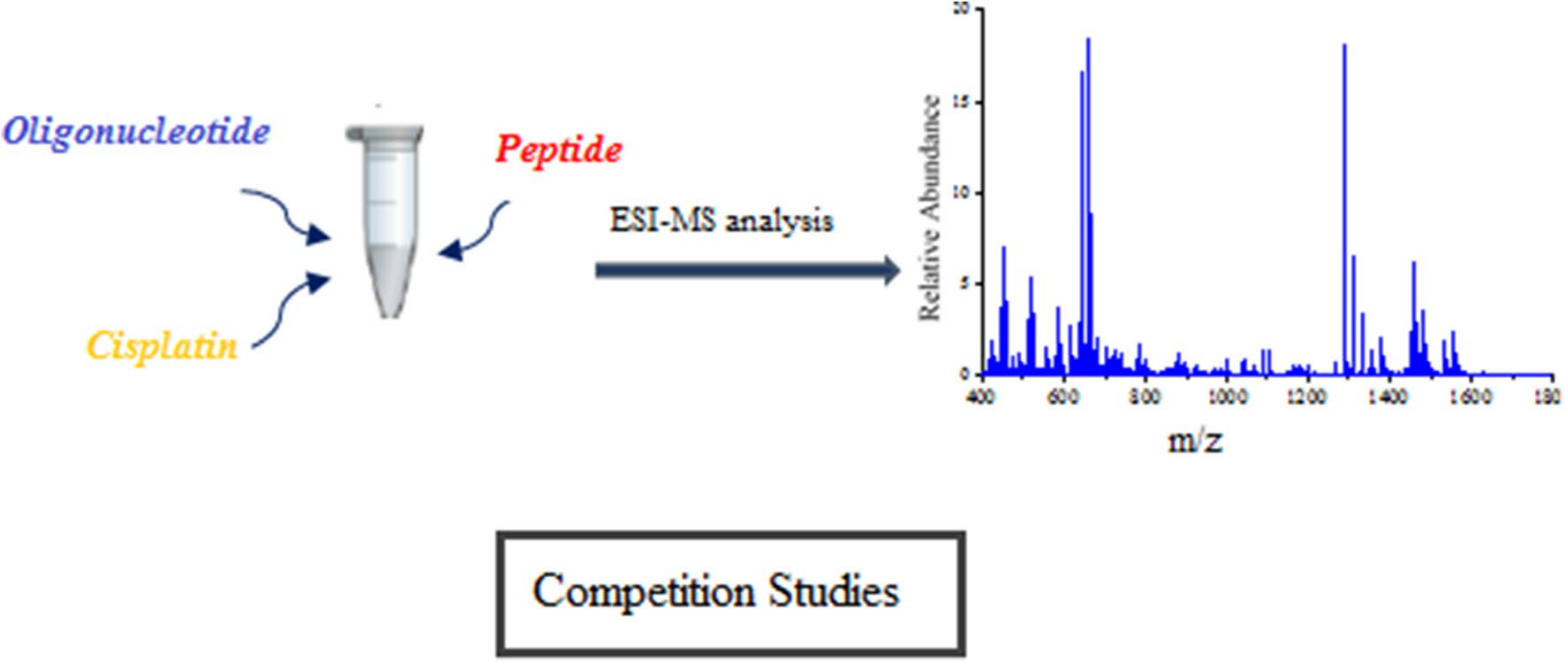

**Supplementary Information:**

The online version contains supplementary material available at 10.1007/s00775-022-01924-9.

## Introduction

Metal complexes exert a wide range of therapeutic effects [1, 2] and consequently are extensively used in the diagnosis and treatment of diseases [3]. Among these metal complexes, *cis*-diamminedichloroplatinum(II), i.e. cisplatin, is the most commonly used drug in cancer chemotherapy [4]. Cisplatin is a prodrug, and once taken up by a cancer cell, it undergoes aquation and the hydrolysed (activated) forms of cisplatin react with DNA to form intra-strand or inter-strand cross-links [5, 6]. In addition to DNA binding, cisplatin reacts with many proteins [7–10], with certain cisplatin-protein interactions leading to drug resistance [11]. It has been shown that platinum-modified DNA can react further with chromosomal high mobility group proteins HMG1 and HMG2 affording DNA-Pt-HMG-domain adducts, which prevent nucleotide repair [12, 13]. Recent reports have revealed that other proteins, such as cytokeratin, elongation factor and histones, bind to DNA after cisplatin treatment [14, 15], affecting nuclear metabolism and chromatin organization [16].

Interestingly, there is a correlation between cytotoxicity and protein-DNA cross-linking [17, 18], and histone proteins are particularly important because they are involved in the formation of the nucleosome as a structural unit of chromatin. Crystallographic studies have shown that platinum-based drugs form extensive adducts with the nucleosome, which appears to be particularly susceptible as sequences which lack preferential targets such as GG or AG sites are platinated, implying that reactivity is enhanced by the histone-bound configuration of DNA or by the proximity of histone proteins [19, 20]. Indeed, guanine platination is largely controlled by solvent/steric access to the N7 atom, which is moreover modulated by the histone-bound DNA conformation [21].

**Fig. 1:**
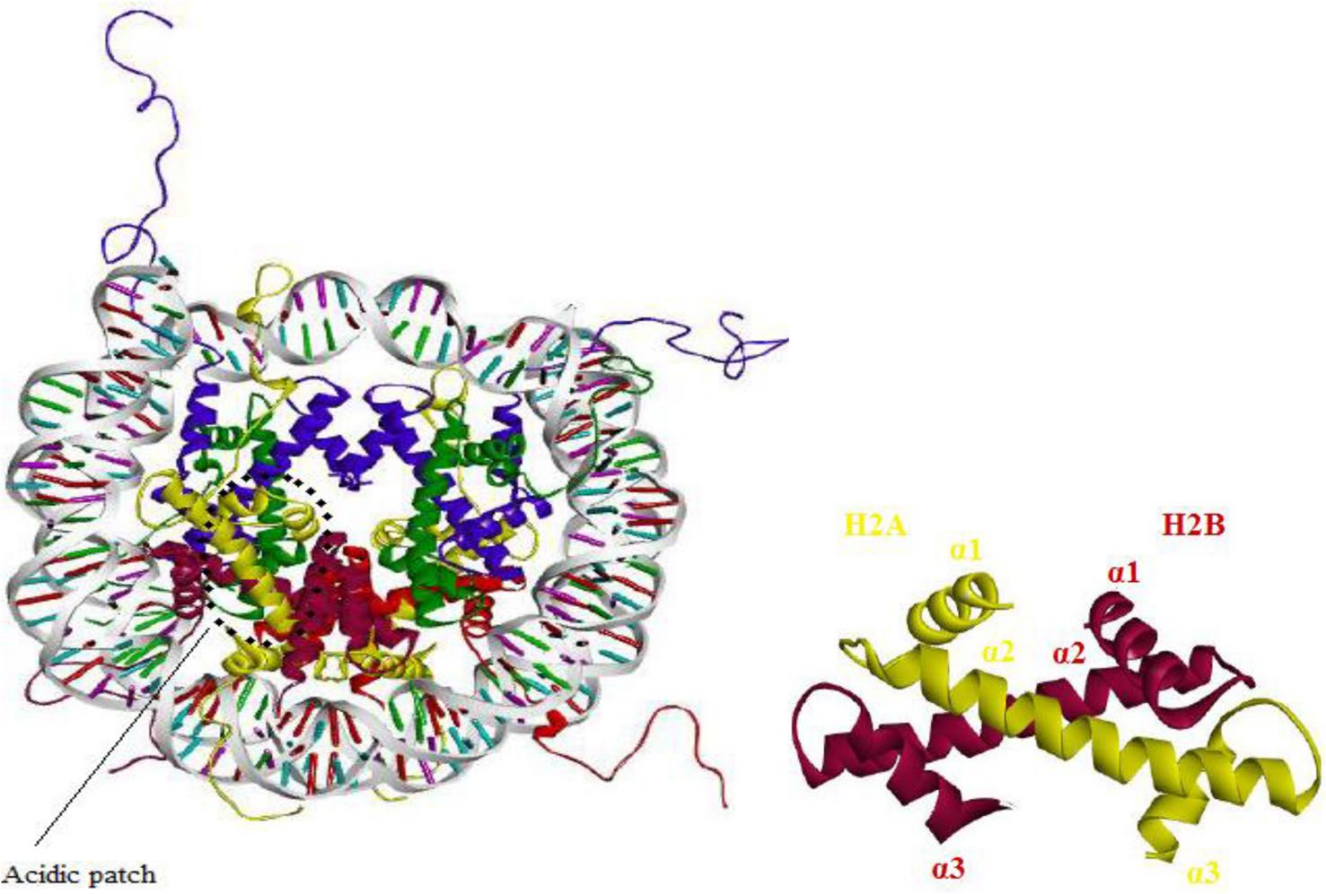
The crystal structure of the nucleosome and the histone-fold heterodimer. (left) Nucleosome crystal structure based on [[22] PDB ID: 1KX5]. H2A is shown in yellow, H2B in red, H3 in blue, H4 in green and DNA in light grey. Acidic Patch is shown with a dotted circle [23]. (right) Structure of H2A/H2B histone-fold heterodimer. Figures were generated using Discovery Studio Visualizer (DSV) 2019

In the nucleosomal structure, H2A-H2B dimers located at the outer turn of the nucleosome can be removed and exchanged more easily than the stable H3-H4 core [24, 25]. The *α*1–*α*2 helixes and acidic patch region on H2A-H2B have a high binding affinity for nuclear factors that influence DNA accessibility, see Fig. 1 [23, 26]. These regions include electronegative (glutamate and aspartate) and also sulphur-donor (methionine) residues that are prone to metal binding [27–29]. Reactivity studies of the model compound [PtCl(dien)]^+^ with peptides and proteins that have sulphur donor atoms show that the resulting Pt–thioether bond may be cleaved with the transfer of the platinum-fragment to the N7 of guanine of an oligonucleotide [30–33]. Moreover, methionine residues of H3 and H4 were seen to accumulate cisplatin and oxaliplatin adducts in crystallographic studies of platinum drug-treated nucleosome core particle [19, 21]. Consequently, platinum-protein adducts may serve as a type of reservoir, storing the platinum adduct and then later releasing it to bind to DNA. Such a mechanism might explain, at least in part, why platinum-based drugs form extensive adducts with the nucleosome.

Herein, we describe the binding of cisplatin to an oligonucleotide model of DNA and two peptides, P1 from the acidic patch region of H2A and P2 from the *α*2-helix of H2B human histone protein, using electrospray ionisation high-resolution mass spectrometry (ESI-HRMS). The web-based tools Aom^2^s [34] and Apm^2^s [35] were used to assign and select parent platinated ions for fragmentation. Thousands of theoretical fragments ions generated by the tools were matched with experimental MS/MS fragments and results, returned as free and platinated fragment ions with similarity scores, allowed the preferred binding sites of cisplatin on the oligonucleotide and peptides to be determined. In addition, competitive cisplatin binding between the oligonucleotide and the peptides and the possibility of platinum-adduct transfer from platinated P_2_ to the oligonucleotide were studied.

## Materials and methods

### Reagents

HPLC-purified double-stranded 13-mer oligonucleotide 5^'^-GTATTGGCACGTA-3' (S1)*,* 5^'^-TACGTGCCAATAC*-*3' (S1c) was obtained as 0.2 mM aqueous solutions from A/S Technology (Denmark). Ammonium acetate, ammonium bicarbonate (> 99.99%), HPLC-grade solvents (water, methanol and n-propanol) and cisplatin were purchased from Sigma-Aldrich (Buchs, Switzerland). HPLC-purified, desalted and TFA-absent peptide VLEYLTAEILE (P_1_) and peptide SKAMGIMNSFVNDIFERIAGEASRLAHY (P_2_) were purchased from LifeTein Company (USA).

### Oligonucleotide double-stranded binding studies

Cisplatin and oligonucleotide were incubated in a (3:1) molar ratio in MilliQ water (pH 7) at 37 °C in the dark for 50 h. After incubation, excess cisplatin was removed using a 3 kDa-cutoff Amicon ultra centrifugal filter. Prior to Electrospray ionization High-Resolution Mass Spectrometry analyses (ESI-HRMS), samples were diluted 1:10 with a solution of 1.1 mM ammonium acetate in water-n-propanol-methanol (30:5:65) to obtain a final DNA concentration of 2 μM. Dilution decreases the exposure of oligonucleotide to high concentrations of organic solvents required for suitable spraying of the samples [36]. To minimize alkali metal cation adducts of oligonucleotide, ammonium acetate was used to exchange Na^+^ and K^+^ with ammonium and samples diluted immediately prior to MS analyses.

### Peptide binding studies

Cisplatin and P_1_/P_2_ were incubated in a (2:1) molar ratio in MilliQ water (pH 7) containing 0.7%-ammonium bicarbonate (0.8 mM) at 37 °C in the dark for 48 h. Prior to injection into the mass spectrometer the samples were diluted 1:50 with an aqueous solution of 50% MeOH and 1% CH_3_COOH to obtain a final peptide concentration of 0.4 μM [37, 38].

### Competition studies

Cisplatin was incubated with the double-stranded oligonucleotide and P_1_ or P_2_ in a molar ratio of (3:1:1) in MilliQ water (pH 7) at 37 °C in the dark for 50 h. The ratio equals approximately 1 platinum per 4 base pairs of 13-mer oligonucleotide. To determine the platinated adducts of the oligonucleotide and peptides, each sample was analysed by ESI–MS in both negative and positive ion modes (see Instrumentation below). For negative mode ESI–MS analysis of platinated oligonucleotides, each sample was diluted 1:10 with a 1.1 mM solution of ammonium acetate in water-n-propanol-methanol (30:5:65) to obtain a final oligonucleotide concentration of 2 μM. For positive mode ESI–MS analysis of platinated peptides, an aqueous solution with 50% MeOH and 1% CH_3_COOH was used to dilute the samples to 0.4 μM final concentrations of peptides*.*

### Transfer studies

Cisplatin and P_2_ were incubated in a molar ratio of (3:1) in MilliQ water (pH 7) at 37 °C in the dark for 48 h. After incubation, excess cisplatin was removed using 3 kDa-cutoff Amicon ultra centrifugal filters and the absence of free cisplatin was checked by ESI–MS (Table S11). The filtered sample was injected into an C18 reverse-phase HPLC column to separate the main cisplatin-P_2_ adducted from the free P_2_. The HPLC purified cisplatin-P_2_ adduct and the oligonucleotide were incubated in a (1:1) molar ratio in MilliQ water (pH 7.5) at 37 °C in the dark for 50 h. The samples were analysed by ESI-HRMS in positive and negative ion modes using the same conditions applied in the competition studies [18].

### Mass spectrometry instrumentation

Mass spectra were recorded on an LTQ Orbitrap Elite FTMS instrument (LTQ Orbitrap Elite FTMS, Thermo Scientific, Bremen, Germany). For operation in negative mode, the orbitrap was interfaced with a HESI-II probe in an Ion Max ion Source. The ionization voltage was set at  − 1.2 kV and the ion transfer capillary temperature at 120 °C. For operation in positive ion mode, the orbitrap was interfaced with a robotic chip-based nano-ESI source (TriVersa Nanomate, Advion Biosciences, Ithaca, NY, USA). Standard data acquisition and instrument control system were used (Thermo Scientific), and the ion source was controlled by the Chipsoft 8.3.1 software (Advion BioScience). Samples were loaded onto a 96-well plate (Eppendorf, Hamburg, Germany) with an injection volume of 5 µl. The ionization voltage was set at + 1.4 kV, the gas pressure at 0.30 psi and the temperature of ion transfer capillary at 200 °C. For tandem MS data analysis, platinum-containing adducts were fragmented by collision-induced dissociation (CID) in the linear ion trap using an isolation window of 8 Da, with product ion detected in the Orbitrap with a resolution set to 120 K.

### Mass spectrometry data analysis using Aom^2^s and Apm^2^s

High-resolution fragmentation mass spectra of the platinum-adducted oligonucleotides were analysed by the automated tool termed Analysis of Oligonucleotide Modifications from Mass Spectra (Aom^2^s, https://mstools.epfl.ch/am2s/). This web-based tool written in pure JavaScript is also accessible from the ms.epfl.ch webpage and should be opened through the Google Chrome browser. Aom^2^s calculates theoretical MS and MS/MS ions from oligonucleotide sequences with desired adducts as well as any user-defined fixed and variable modification. Most important parameters to properly use the tool are described in the Aom^2^s web page, as well as in SI and in [34]. For tandem MS experiments, the user specifies a list of allowed fragments from cleavage at the 3′ and 5′ ends including base-losses and neutral losses, as well as internal fragments. The system combines all possible fragments and generates a list of theoretical ions. Subsequently, Aom^2^s automatically matches theoretical isotopic patterns to the experimental isotopically resolved mass spectrum loaded as txt file, yielding a list of matches ranked by 75% similarity scores. Aom^2^s automatically generates a final graphic representation of the oligonucleotide as a fragment map. All the calculations are performed locally in the browser, therefore with secured data handling. CID fragmentation of platinated peptides was also analyzed by online Apm^2^s tool (available on https://mstools.epfl.ch/am2s/) which facilitate metal-protein binding studies by identification of all types of internal and terminal fragments. Table S1 includes general input parameters and data interpretation restrictions applied for data processing using Aom^2^s and Apm^2^s.

## Results and discussion

Many bioanalytical, biophysical and hyphenated techniques have been used to study metallodrug-biomolecular interactions [39]. Mass spectrometry with different ionization conditions and mass analysers are particularly valuable for determining the binding sites of cisplatin and related compounds to oligonucleotides and proteins [36, 40]. Mass spectrometry data can, however, be difficult to interpret as thousands of peaks are often generated and isotopic patterns frequently overlap. To overcome this challenge we used the web-based tools Aom^2^s [34] and Apm^2^s [35] to automate exhaustive mass spectra matching for the oligonucleotide and peptide binding studies, respectively.

### Oligonucleotide binding studies

**Fig. 2:**
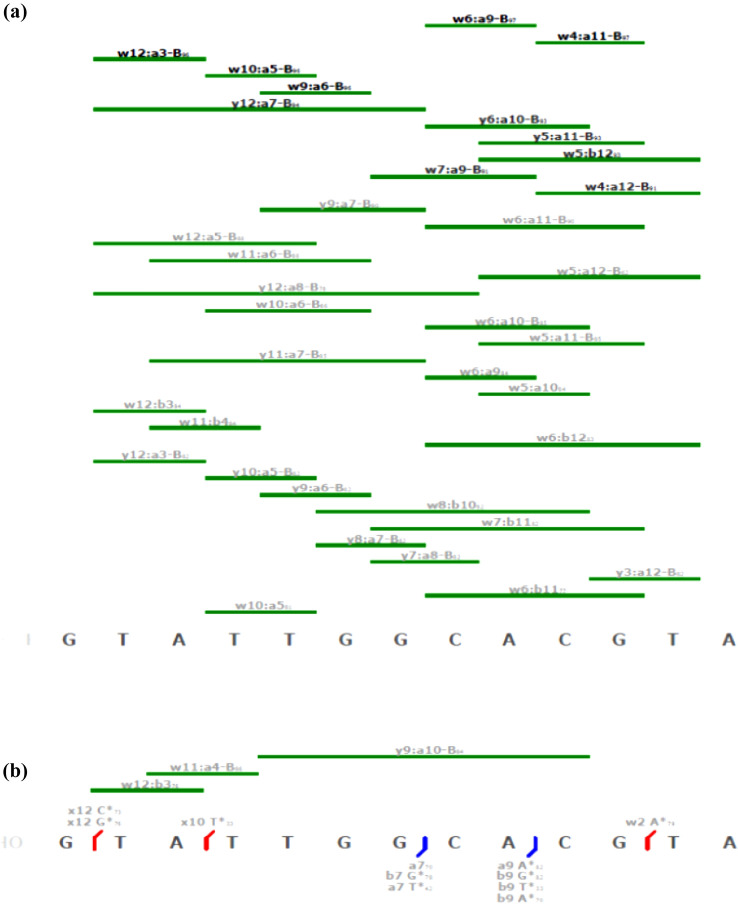
Fragment maps for the S1 oligonucleotide representing platinated fragment ions after CID fragmentation of [S1 + Pt(NH3)_2_-8H]^6−^ at *m/z* 701.4438. **a** Unplatinated internal fragments. **b** Platinated fragments. The maps show identified **a**-**b** in blue, *w*–*x* in red and internal fragment ions are displayed as green bars. Modified bases are marked in purple. Fragment types marked in black: average similarity > 90%; fragment types marked in gray fragments: average similarity < 90%

ESI-HRMS of the 13-mer double-stranded oligonucleotide, i.e. 5^'^- GTATTGGCACGTA-3' (S1)*,* 5^'^-TACGTGCCAATAC*-*3' (S1c), containing preferential GG and GTG binding sites, incubated with cisplatin, was recorded in negative ion mode. The Aom^2^S tool was first used to rapidly track platinated adducts of the double-stranded oligonucleotide in the full scan mass spectrum, and 23 platinated ion species with a similarity above 90% could be assigned (Figure S5). Singly platinated adducts corresponding to [S1 + Pt(NH_3_)_2_-8H]^6−^ and [S1c + Pt(NH_3_)_2_-8H]^6−^ were observed at *m/z* 701.4438 and 689.6103, respectively. These adducts were fragmented by CID and, as expected, the main fragments resulting from cleavage of the oligonucleotide were *a/w* and a-B internal fragments. Nevertheless, all fragment types except d-H_2_O and z-CH_2_ were used to determine the precise cisplatin binding sites on the oligonucleotide. For strand S1, a total of 100 fragment ions were identified with similarity scores above 75%, among them 22 were platinated. About 39 ions were identified as internal fragments of which 3 were platinated (Tables S2 and S3). Corresponding fragment maps for S1 are shown Fig. 2, including both unplatinated internal fragments (Fig. 2a) and platinated fragments (Fig. 2b). For S1, the smallest assignable Pt-containing fragment with a similarity > 80% was a9-B, which contains the expected GG binding site. The Pt-containing fragments a7-B(G) containing the expected GG site were found with a lower similarity score of 78%. Analysis of the fragmentation map of unplatinated internal fragments reveals that a majority of unplatinated internal fragments are more frequently located than GG. The platinated *w*2 fragment at *m/z* 846.0975 is isobaric with the platinated internal fragments W12:b3 and w11:b4. For S1c a total of 135 fragment ions were identified with similarity scores above 75% (Table S4), among them 58 were platinated (Table S5). Once platinated internal fragments were identified out of the 19 internal fragments ions assigned. The corresponding fragment maps for S1c are shown in Figure S6, for both unplatinated internal fragments (Figure S6a) and platinated fragments (Figure S6b). For S1c, the smallest assignable Pt-containing fragments a6 at *m/z* 670.7721 (3 charges, Pt(NH_3_)_2_) and a6-B(G) at *m/z* 913.1376 (2 charges, Pt(NH_3_)_2_) were assigned with similarities of 95% and 85%, respectively. They contain the expected GTG binding site reported elsewhere [36]. The fragmentation map of unplatinated internal fragments of S1c (Figure S6a) shows that the vast majority of unplatinated internal fragments are more frequently located after GTG, such as w7:a8-B (*m/z* 466.0422, similarity 94%), *w*7:a10-B (*m/z* 1068.1462, similarity 95%), or w7:a12-B (*m/z* 842.1213, similarity 90%). Around 72% of platinum-free internal fragments do not include the GTG subsequence. For both strands S1 and S1c, the platinated terminal fragments contain the preferential GG and GTG binding sites. Tandem mass experiments were conducted in parallel on unplatinated single-stranded species [S1-5H]^5−^ at 796.533 *m/z* and [S1c-6H]^5−^ at 782.3331 *m/z* and the list of assigned fragments are displayed in Tables S6 and S7. As shown in Figure S7, internal fragments obtained for [S1-5H]^5−^ and [S1c-6H]^6−^ all include GG and GTG such as [w10:a9-B-2H]^2−^ at *m/z* 865.6121 (similarity 89%) and [w10:a9-B-3H]^3−^ at *m/z* 576.7425 (similarity 81%) for S1 and [w10:a9-B-2H]^2−^ at *m/z* 858.1162 (similarity 93%), [w10:a10-B-3H]^2−^ at *m/z* 1014.6450 (similarity 96%), [W10:a6-B-2H]^1−^ at *m/z* 810.0944 (similarity 91%) and [W10:a7-B-2H]^1−^ at *m/z* 1139.1469 (similarity 96%) for S1c. Those internal fragments are not detected in the corresponding platinated strands, presumably due to platination of the GG and GTG regions. Platination of the internal part of the strands results in the formation of longer platinated internal fragments which span the entire length, such as y9:a-B10 for S1 and y11:a12-B for S1c.

**Fig. 3:**
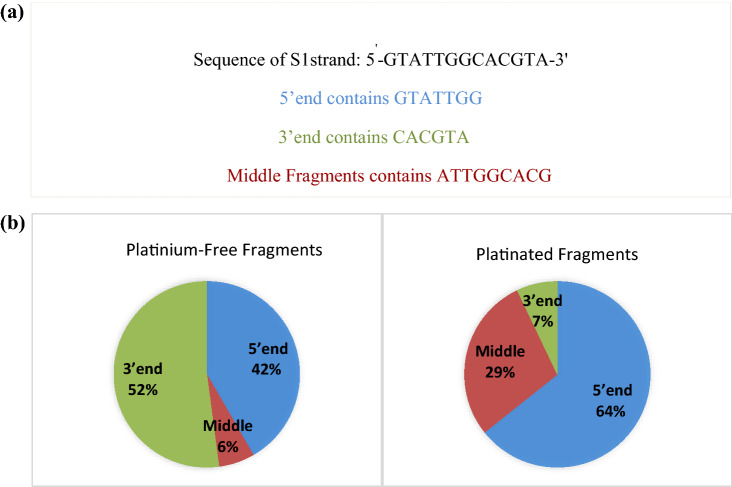
** a** Classification of 5^'^-end (in blue), 3'-end (in green) and Middle fragments (in brown) of S1 strand of double-stranded oligonucleotide. **b** Percentage abundance of 5^'^-end, 3'-end and Middle fragment ions from platinum-free fragmentation and platinated fragment from CID fragmentation of the peak at *m/z* 701.4438 for the S1 strand

Percentage values of nucleotide fragments observed for both the Pt-containing and Pt-free samples further confirm preferential binding of cisplatin at the GG and GTG regions due to the higher frequency of platinated fragments in these regions (Fig. 3, Figs. S8 and S9). From Fig. 3a and b, it is clear that the 3’ end participates more frequently in the formation of platinum-free internal fragments and less frequently comprises platinated fragments, i.e. this end is less favorable for metal binding, whereas the 5’ end including the GG subsequence has a higher percentage of platinated fragments. Cleavages leading to the formation of the platinated a/b/a-B/b-B fragments are located only on the 3’ position of the GTG and GG sequences. This contrasts with the fragmentation pattern of the free oligonucleotide, and may be attributed to local conformational changes of the strands at the platinated sites (i.e. B-DNA changes to A-DNA) [41]. This conformational change may result in a decrease of cleavages, leading to the formation of platinated internal fragments in the middle part of S1 and S1c strands. Hence, the platination percentage in the middle region is less than that of the 5’ end (Fig. 3b). In addition, the middle region includes the preferential GG metal-binding site and consequently leads to fewer platinum-free internal fragments (Fig. 3a).

### Peptide binding studies

**Fig. 4:**
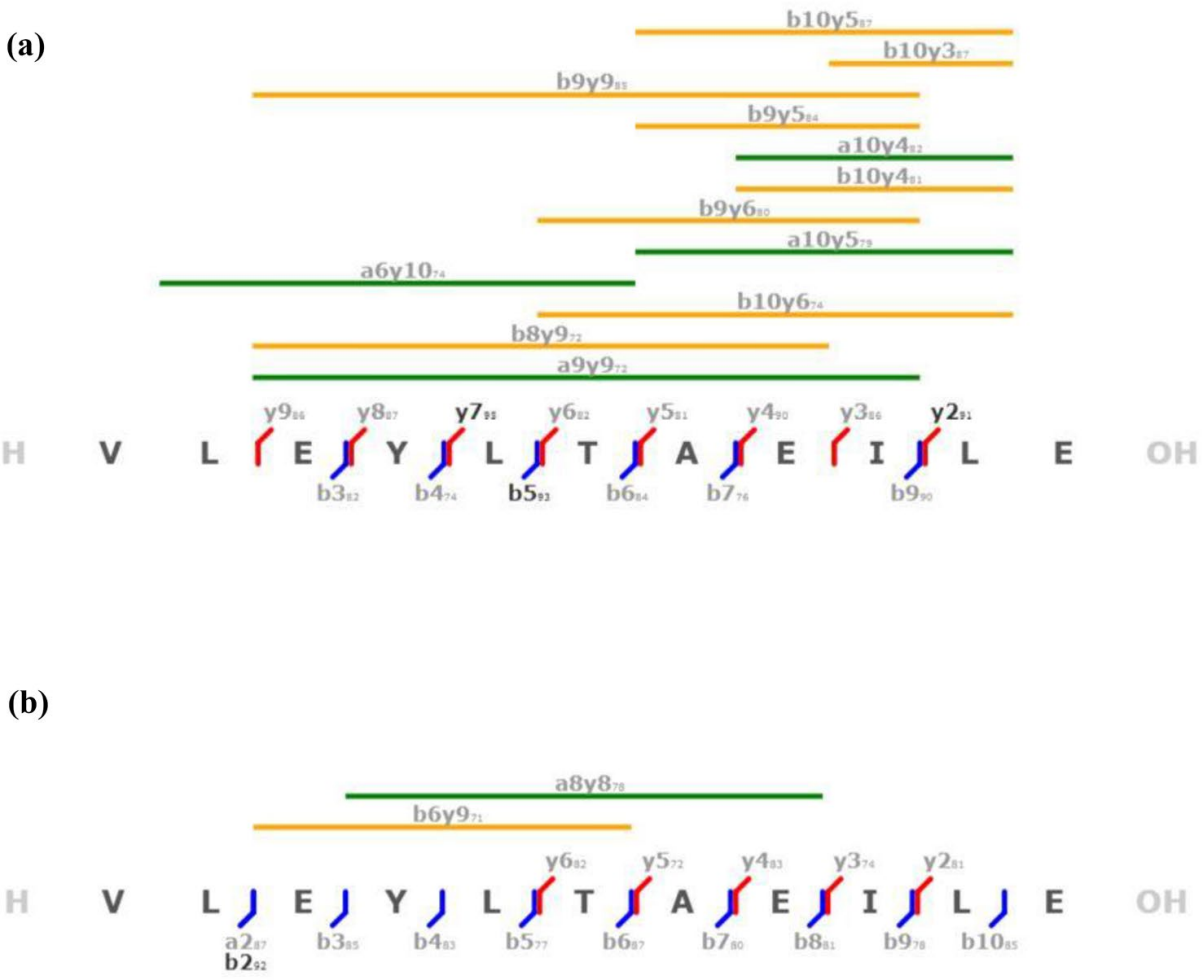
Fragmentation maps of peptide *P*_1_ after CID fragmentation of the [*P*_1_ + *P*t(NH_3_)_2_]^2+^ ion at *m/z* 760.3546. **a** Unplatinated fragments. **b** Platinated fragments. The maps show identified **a**/**b** type in blue, y type in red and internal fragment ions displayed as green and orange bars. Fragment types marked in black: average similarity > 90%; fragment types marked in gray fragments: similarity < 90%

**Fig. 5:**
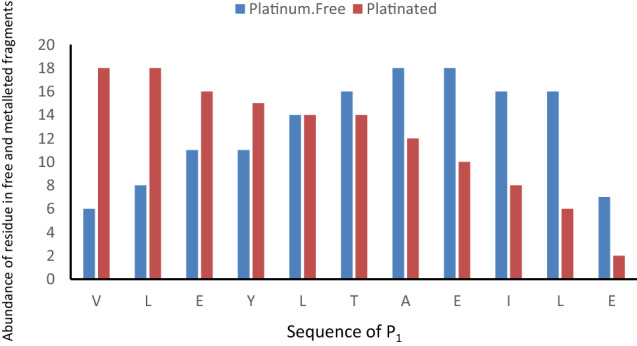
Location of cisplatin binding site in *P*_1_: Histogram showing the occurrence of all assignable amino acids for the unplatinated and platinated fragments resulting from CID fragmentation of the [*P*_1_ + *P*t(NH_3_)_2_]^2+^ ion at *m/z* 760.3546

Two peptides from the outer core of the nucleosome, P_1_, from the acidic patch region of H2A (VLEYLTAEILE), and P_2_, the α_2_-helix of H2B (SKAMGIMNSFVNDIFERIAGEASRLAHY), were used for the study as these regions were previously shown to interact with metallodrugs [27]. For P_1_ incubated with cisplatin, the ion at *m/z* 760.3546 corresponding to the [Pt(NH_3_)_2_]^2+^ adduct was selected for CID fragmentation. By analysing platinated and unplatinated *b*/*y*-type and internal fragments of the peptide [P1 + Pt(NH_3_)_2_]^2+^ ion (Table S8, Fig. 4), it was found that the platinum-free fragments are mainly y-type e.g. [y9 + H]^+^ at *m/z* 1080.5487 and fewer b-type are platinum-free e.g. [b5 + H]^+^ at *m/z* 1032.5617, whereas the platinated fragments are mostly b/a-type, e.g. the shortest platinated fragment [b2 + Pt(NH_3_)-H]^+^ at *m/z* 423.1354, [a + Pt(NH_3_)-H]^+^ at *m/z* 395.1405 and also some platinated y-type detectable such as [y4 + Pt(NH_3_)_2_-H]^+^ at *m/z* 730.2734. Most of unplatinated fragments are distributed of the C-terminal part of the peptide, after L5/T6. According to the abundance of residues in the platinum-free and platinated fragments as shown in the histogram Fig. 5, the N-terminal region has a greater tendency to bind to cisplatin fragments. Hence most of the internal fragments appear to be unplatinated and span the whole peptide sequence except the N-terminal end.

The full mass spectrum of P_2_ incubated with cisplatin is shown in Figure S10 with the main peaks assigned to platinated species, including both mono-adducts, i.e. [P_2_ + Pt + 3H]^5+^, [P_2_ + Pt + 2H]^4+^ and [P_2_ + Pt(NH_3_) + 3H]^5+^ at *m/z* 664.9087, 830.8790 and 668.3100, respectively, and bis-adducts, i.e. [P_2_ + 2Pt(NH_3_) + H]^5+^ at *m/z* 710.3051 and [P_2_ + 2Pt(NH_3_)]^4+^ at *m/z* 887.6296. [P_2_ + Pt + 2H]^4+^, indicates that tetradentate coordination with the P_2_ peptide from H2B histone protein can occur. Fragmentation of the [P_2_ + Pt + 3H]^5+^ ion was performed and the resulting map of platinated fragment ions is displayed in Figure S11 (Table S9 for fragment list), assigned platinated b/y-type fragments with high similarity, i.e. [y25 + Pt + 2H]^4+^ at *m/z* 759.3395 and [b17 + Pt + H]^3+^ at *m/z* 711.9680 covering the region between residues 4 and 17. This region includes two methionine, aspartate and glutamate residues, which are known to have a high binding affinity to metal ions [8, 35, 42]. Hence, although P_2_ has a more stable and less flexible structure than P_1_, these residues increase its propensity for binding cisplatin fragments as suggested by the presence of bis-platinated P_2_ adducts [43]. In particular, two methionine and a histidine residue distinguish P_2_ from P_1_, although both have multiple carboxylate residues. MS and crystallography studies indicate that methionine, cysteine, aspartate and histidine side chains in small proteins preferentially targeted by cisplatin [44–47]. Crystallographic studies of cisplatin-treated nucleosomes reveal pronounced platination of solvent-exposed methionine residues [19, 21], suggesting they are the primary sites of adduct formation in P_2_ and a recent crystallographic study shows that cisplatin-protein adducts retain a certain degree of flexibility and reactivity [48].

### Competition and platinum adduct transfer studies

To establish preferential reactions of cisplatin with DNA and histone proteins in the nucleosome, the double-stranded oligonucleotide and *P*_1_ or *P*_2_ were co-incubated with cisplatin in a molar ratio of (1:1:3) for up to 50 h. For the co-incubation with *P*_1_, after 50 h the highest relative abundance peak in the positive ion spectrum is observed at *m/z* 646.85 corresponding to free *P*_1_ and platinum adducts are not detected (Figure S12a). In the negative mode spectrum, a low intensity platinated *P*_1_ ion is detected (*m/z* 758.34, *z* = 2), but it represents < 1% of unplatinated *P*_1_. In contrast, in negative mode unplatinated oligo S1/S1c was not observed whereas significant amounts of platinated S1 and S1c ions were detected (Figure S12b). Several peaks assignable to [Pt(NH_3_)_2_], bis-[Pt(NH_3_)_2_] and tri-[Pt(NH_3_)_2_] adducts of the oligonucleotide are observed (Table 1 shows the assigned ions).

**Table 1:** List of adducts (free and platinated) obtained in negative ion mode using the Aom^2^s /Apm^2^s tool for incubation of peptide P1/Oligonucleotide/cisplatin in a ratio of (1:1:3) for up to 50 h

MF	Ionization	MF Mass	Th m/z	Exp m/z	ppm	Charge	Intensity	Similarity
C128H179N55O77P12Pt3(+6)	(H+)-9	4674.7577	1555.2296	1555.2323	1.68	− 3	1.91	0.99
C126H172N52O75P12Pt2(+4)	(H+)-7	4374.7391	1455.8953	1455.8983	2.07	− 3	4.85	0.99
C128H173N53O77P12Pt2(+4)	(H+)-7	4445.7398	1479.5622	1479.5655	2.22	− 3	2.47	0.98
C126H172N52O75P12Pt2(+4)	(H+)-8	4374.7391	1091.6697	1091.6712	1.40	− 4	1.10	0.98
C126H178N54O75P12Pt3(+6)	(H+)-9	4603.7570	1531.5627	1531.5658	2.02	− 3	1.66	0.96
C126H166N50O75P12Pt(+2)	(H+)-5	4145.7212	1380.2279	1380.2304	1.78	− 3	1.21	0.96
C128H173N53O77P12Pt2(+4)	(H+)-8	4445.7398	1109.4198	1109.4209	0.90	− 4	0.94	0.94
C128H179N55O77P12Pt3(+6)	(H+)-10	4674.7577	1166.1704	1166.1719	1.28	− 4	0.33	0.92
C126H178N54O75P12Pt3(+6)	(H+)-10	4603.7570	1148.4202	1148.4232	2.63	− 4	0.19	0.92
C126H166N50O75P12Pt(+2)	(H+)-6	4145.7212	1034.9191	1034.9207	1.55	− 4	0.32	0.90

In a similar co-incubation experiment using P_2_, only traces of platinated P_2_ adducts are detected, whereas platinated oligonucleotide ions such as [S1 + Pt(NH_3_)_2_-5H]^3−^ at *m/z* 1403.8948 and ([S_1_c + 3Pt(NH_3_)-9H]^3−^) at *m/z* 1531.5627 are abundant after 50 h of incubation (see Table S10 and Figure S13 in the SI for further details on the assigned ions). These competition experiments indicate that for both peptides studied, cisplatin has a considerably greater binding affinity for the oligonucleotide compared to the peptides, in agreement with published studies [32].

The possibility of transferring platinum adducts from platinated P_2_ to the oligonucleotide was then investigated. Cisplatin was incubated with P_2_ and the platinated-peptide was isolated following ultrafiltration and purification by preparative liquid chromatography (Figure S14). The purity of platinated P2 was confirmed by ESI–MS prior to transfer studies (Table S12). Platinated P_2_ was then incubated with the oligonucleotide in a 1:1 ratio and the mixture was analysed after 12, 24 and 50 h. The negative ion mass spectra recorded after 12 and 24 h shows the presence of platinated oligonucleotide such as [S1 + Pt + 2 K-7H]^3−^ at *m/z* 1417.8477 (similarity 90%) and [S1 + Pt + 2 K + Na-8H]^3−^ at *m/z* 1425.1750 (similarity 80%) (Table S13). The negative ion mode spectra are presented in Figures S15 to S17. Although platinated and free P_2_ were not detected in positive ion mode analyses, the free P_2_ ion species [P_2_-2H]^2−^ was detected at *m/z* 1562.2617 (similarity 96%) after 24 h in the negative ion mode. The HPLC purified platinated P_2_ did not contain free P_2_, suggesting that migration of platinum fragments from P_2_ to the oligonucleotide takes place. Hence, binding to the peptide is reversible and the most thermodynamically stable products consist of Pt-oligonucleotide species. These data are in agreement with crystallographic studies of the nucleosome core particle [20] and related transfer studies employing amino acids and nucleobases [30, 49, 50].

### Conclusions

ESI–MS was used to determine the preferential binding sites of cisplatin adducts on two peptides (P_1_ and P_2_) from H2A and H2B histone proteins and an oligonucleotide. The specific search for internal metallated fragment ions was very informative, bringing interesting structural information especially the localisation of drug binding sites. GG and GTG binding sites were found to be most favoured by cisplatin. In addition, methionine, aspartate and glutamate residues were identified as the dominant binding sites in the peptides. Subsequent competition studies between the oligonucleotide and the peptides confirmed that cisplatin reacts preferentially with the oligonucleotide. Moreover, platinum fragments bound to the peptides may dissociate and be transferred to the oligonucleotide. These studies have implications on the mechanism of action of cisplatin and potentially other platinum-based drugs in the treatment of cancer. Indeed, cisplatin may bind first to histone proteins directly, then the bound adducts could transfer to the DNA to form platinated oligonucleotide at the favored GG site. These processes are involved in damaging the DNA and inhibiting cellular proliferation. Our results suggest that the H2A and H2B histone proteins, in addition to H3 and H4, may play role in the mechanism of action of cisplatin and could be implicated in the prevalence of extensive platinum adducts observed in the nucleosome. The more freely exchanging H2A/H2B dimers may also provide a means for the long-range transfer of platinum drug adducts to distantly related chromatin sites.

## Supplementary Information

Below is the link to the electronic supplementary material.

Supplementary file1 (PDF 2328 KB)
