# Supplementary material for: Simultaneous mass spectrometry analysis of cisplatin with oligonucleotide-peptide mixtures: implications for the mechanism of action
Source: J Biol Inorg Chem. 2022 Jan 22;27(2):239–48. doi: 10.1007/s00775-022-01924-9 (PMC8907109; doi:10.1007/s00775-022-01924-9)
Supplement: Supplementary file 1 — Supplementary file1 (PDF 2328 KB) [file 775_2022_1924_MOESM1_ESM.pdf]

Supporting Information

Simultaneous mass spectrometry analysis of cisplatin with oligonucleotide-peptide mixtures: implications for the mechanism of action

Farangis Mansuri[a,b], Luc Patiny[a], Daniel Ortiz[a], Laure Menin[a], Curtis A. Davey[c,d], Fakhrossadat Mohammadi[b*], Paul J. Dyson[a,*]

[a] *Institute of Chemical Sciences and Engineering, Swiss Federal Institute of Technology Lausanne (EPFL), Lausanne CH-1015, Switzerland*

[b] *Department of Chemistry, Institute for Advanced Studies in Basic Sciences (IASBS), 444 Prof. Sobouti Blvd., Gava Zang, Zanjan 45137-66731, Iran*

[c] School of Biological Sciences, Nanyang Technological University, 60 Nanyang Drive, Singapore637551,Singapore

[d] NTU Institute of Structural Biology, Nanyang Technological University, 59 Nanyang Drive, Singapore 636921, Singapore

Aom^{2}s tool: layout, algorithm, input parameters and data interpretation S2-S6

Supplementary figures and tables for Oligonucleotide-Cisplatin studies S7-S26

Supplementary figures and tables for P_1-Cisplatin studies S27-S28

Supplementary figures and tables for P_2-Cisplatin studies S29-S34

Supplementary figure for competitional interaction of cisplatin with P_1/P_2 and oligonucleotide S35-S38

Supplementary figure for transfer study S39-S44

References S45

Aom^2s tool: layout, algorithm, input parameters and data interpretation

The Aom^2S tool as recently described elsewhere [1], [2] and a complete tutorial can be found at https://ms.epfl.ch/applications/oligonucleotides/. Aom^2S is made up of a webpage consisting of three main modules (*Home, Peak picking and recalibration, Report*). The *Home* module is shown Figure S1 for the oligonucleotide S1, whereas the *Settings window* is described in Figure S2.

The first step for processing HR-MS/MS data with Aom^2S involves the *Settings* window (Figure S1A) where calculation parameters need to be fine-tuned. For the stands S1 and S1c studied here, typical adducts allowed were the removal up to 11 protons and written (H^+)-1-11 and the 3 variable groups allowed were (Pt^{++}), $(Pt(NH_3)^{++}$, $(Pt(NH_3)_2{}^{++})$. Various MF filters can be added, if needed. Then spectrum filters should be set in the *Spectrum Filters* section (Figure S2II) to correct peak picking and isotopic profile matching between theoretical and experimental data. These filters include: relative peak intensity threshold (typically 0.01), allowed experimental mass error in ppm (typically 5 ppm), and minimum similarity for peak matching (typically 80 %). Zone parameter refers to the comparison zone to be taken for analysis based on the monoisotopic peak position (typical values -0.5 to 3.5). In our particular case with Pt containing fragments, it should be extended to -1.5 to 5.5 to provide the coverage of the whole isotopic pattern. The expected fragment ion types should be indicated in the *MS/MS fragment types* section and the fragment nomenclature used is the one established by McLuckey *et al* [3]. and shown Figure S3. The Table S1 summarizes the settings parameters used for the binding studies.

In a second step the experimental raw data is loaded by simply copying the data and directly pasting it as a txt file into the specially marked window (Figure 2IV). Once data are inserted, the experimental mass spectrum is reconstructed in red within the *Mass spectra* window. For matching, each individual isotopic peak in the theoretical spectrum is represented as an isosceles trapezoid with bottom and top widths related to the mass accuracy and resolution of the experimental spectra. As the top and bottom width values depend on the *m/z* and the type of mass analyzer used, these widths can be determined by automated peak picking of the experimental spectrum and generation of a regression curve of the peak width then applied to the simulated spectra (Figure S4). Typically, the bottom width of the trapezoid is set as 3 times the width obtained from regression, while bottom width is set at the top width. For the matching process, the

comparison zone is defined relative to the monoisotopic peak. Subsequently, the sum of intensities of all peaks in the defined zone is normalized to 1 and each individual peak in the experimental spectra is matched against its simulated peak and the similarity score is calculated as: $\boldsymbol{Similarity} = (\mathbf{1} - \boldsymbol{sum\ of\ non\ matched\ height\ of\ experimental\ peaks}) * \mathbf{100}\%$

In a third step the tool matches experimental to theoretical peaks and generates a list of matched fragment ions with similarity scores. An additional post calibration step available in the *Peak picking and recalibration* module can be performed to improve mass accuracy and, therefore, similarity scores of matched ions. This post-calibration step requires a minimum number of peaks spread over the *m/z* range to be taken, with a minimum similarity, parameters that need to be defined by the user. After recalibration, a new corrected distribution of errors (in Da and ppm) between theoretical and experimental ions as a function of *m/z* is displayed.

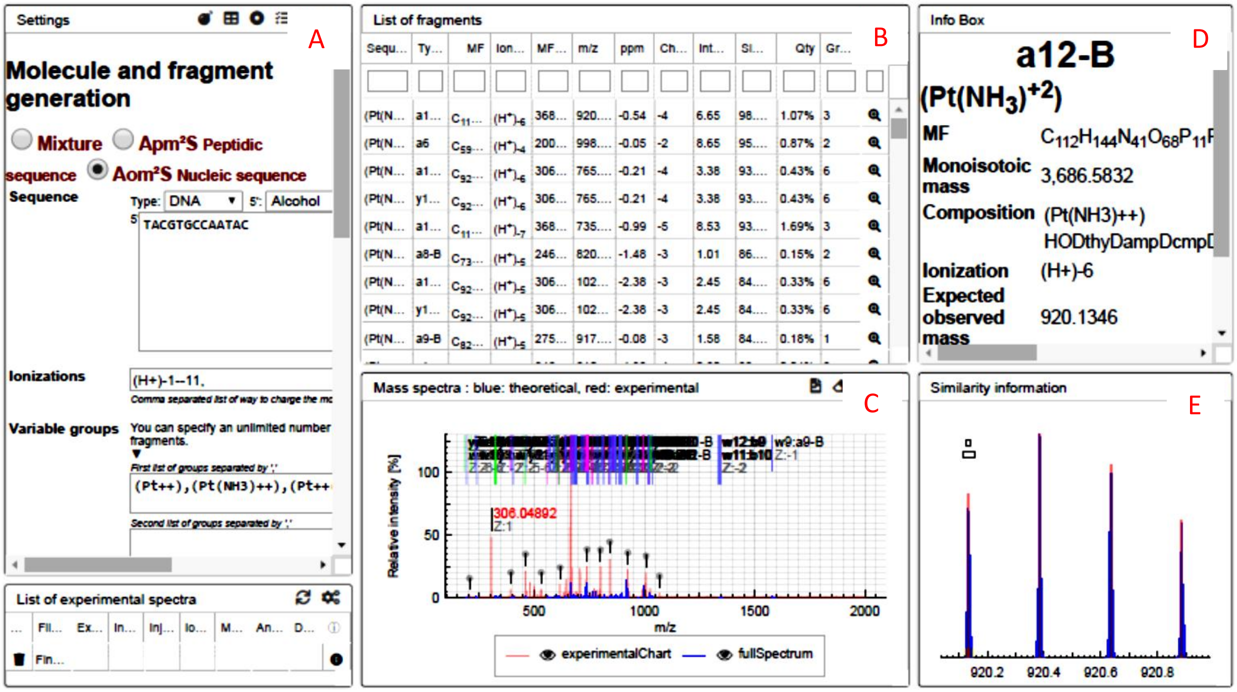

Figure S1. Main Aom²s *Home* module. Windows are labelled A to E corresponding to: (A) Settings (detailed description in Figure S2), (B) result as a list of matched fragments with variable groups, fragment sequence, fragment type, theoretical MF, experimental *m/z*, mass error (ppm), charge, intensity and percentage similarity (C) experimental MS spectra (red) overlaid with theoretical matched fragments (blue). Fragment ions assigned are displayed on the top of the spectra and color coded based on fragment ion type, (D) fragment details for the selected fragments in the list B, (E) *Similarity information* window with zoom on overlaid theoretical (blue) and experimental (red) isotopic patterns for the selected ion, with differences in the intensity of isotopologues highlighted in yellow.

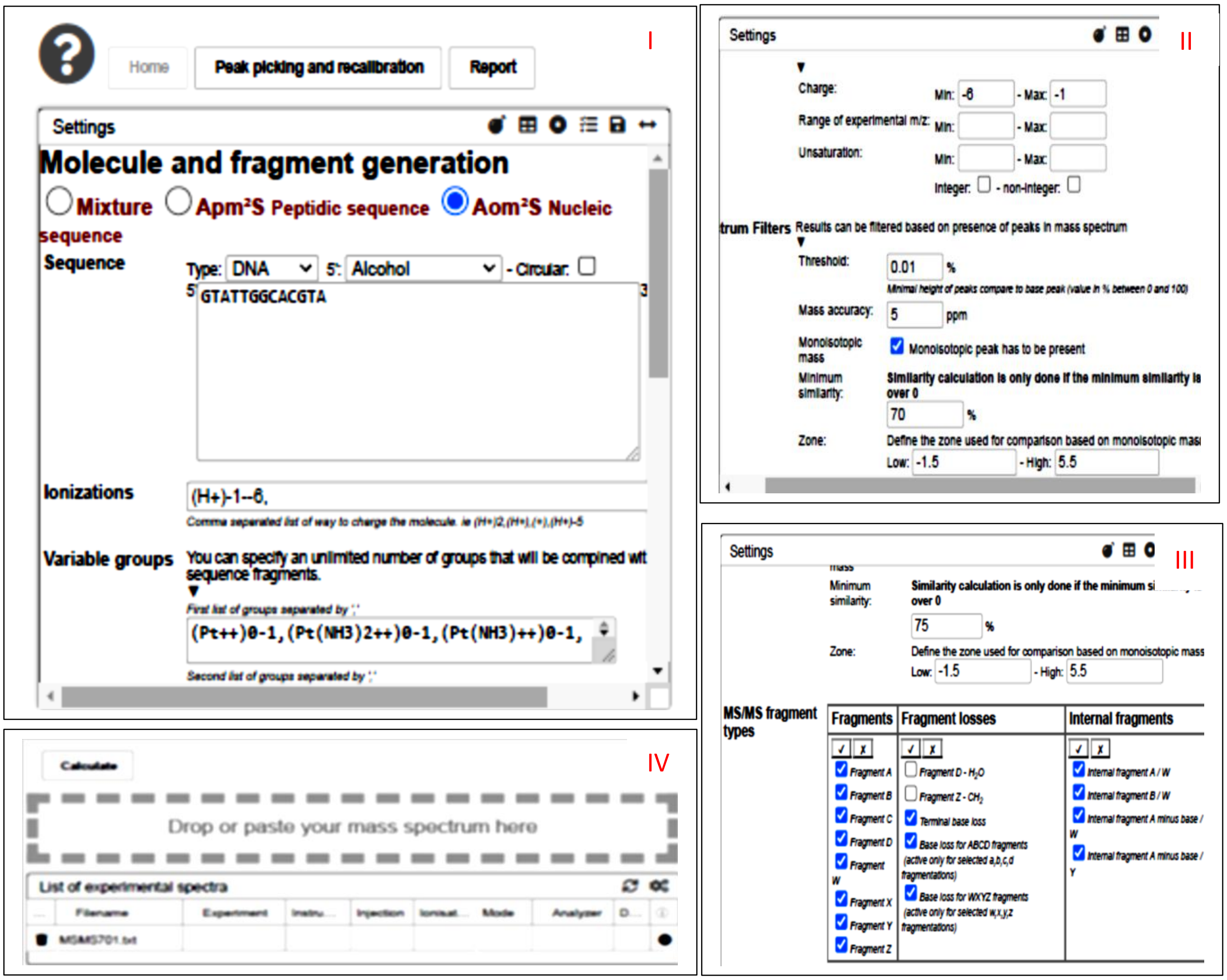

Figure S2. Aom²s *Settings* window (Figure S1, A), labelled I to IV: (I) oligonucleotide type and sequence, with any user-defined adduct and variable group, (II) bottom and top widths for of trapezoid for matching theoretical to experimental spectra, zone which specifies the mass range in m/z where theoretical spectra is overlapped with experimental spectra to calculate similarities (low and high specifies mass range in m/z before and after the monoisotopic mass of the peak of interest respectively), common zone (which specifies how similarity matching between theoretical and experimental spectra is performed), (III) MS/MS experiment parameters including fragment types, internal fragments and length of internal fragments, (IV) drag and drop spectra (accepts.txt files).

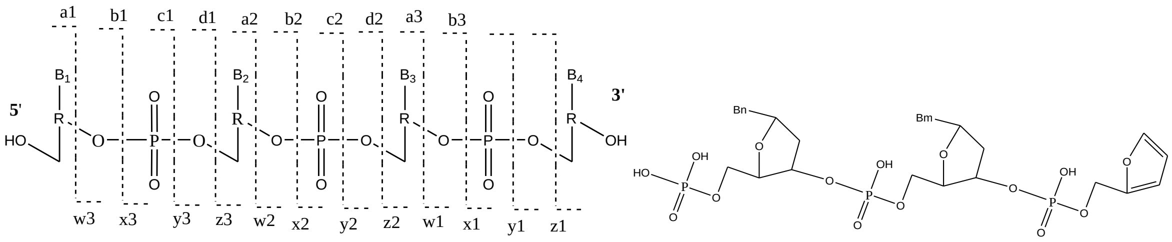

Figure S3. Nomenclature of oligonucleotide fragments observed by tandem MS. (a) a-d fragments correspond to fragments with an intact 5′ terminus and w-z-type fragments have an intact 3′ terminus. (b) Internal fragments resulting from double fragmentation usually occur at the a/w site.

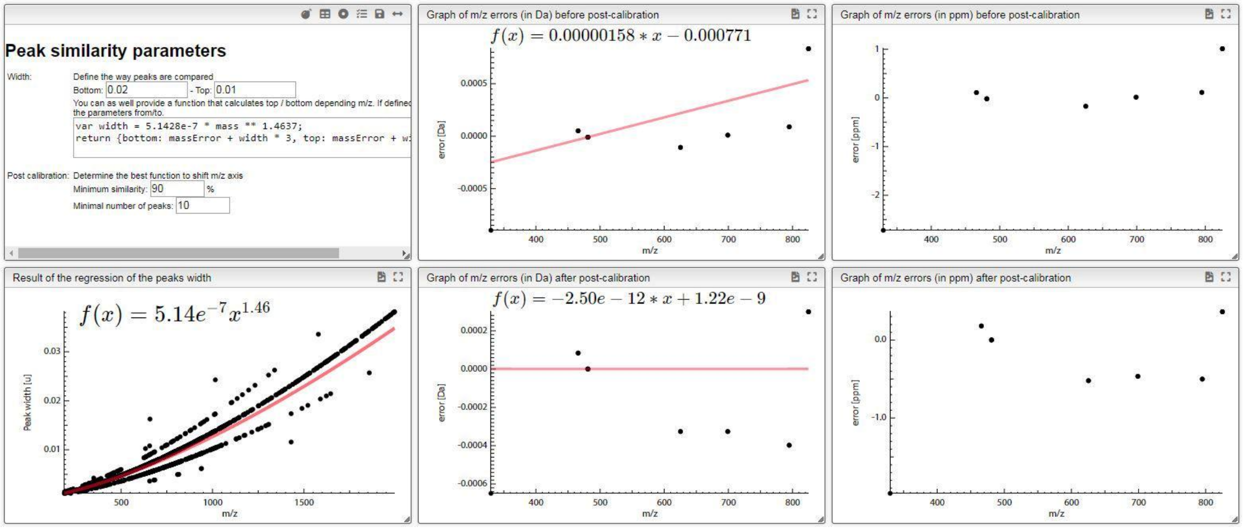

Figure S4. *Peak picking and recalibration* module, of Aom^{2}S for the S1 oligonucleotide tandem mass spectra. Regression curve for peak widths *vs m/z* and error distribution graphs (in Da and ppm) before and after the post calibration step are shown for HR-MS/MS data analysis the ions species $[S1+Pt(NH_3)_2-8H]^{6-}$ at *m/z* 701.4401.

Table S1. Settings parameters used for data processing using Aom^{2}s [1] and Apm^{2}s [4]

	Settings	S1 / S1C MS	S1 / S1C MSMS	Peptides MS	Peptides MSMS P1	Peptides MSMS P2
Sequence	Type	ds-DNA	DNA		peptide	
Sequence	5'	alcohol				
Spectrum filters	Ionization	(H+)-1--11	(H+)-1--6	(H+)1-12	(H+)1-6 (P1)	(H+)0-6 (P2)
Spectrum filters	Variable groups	Pt++, Pt(NH3)++, Pt(NH3)2++,(Pt(NH3)2	Pt++, Pt(NH3)++, Pt(NH3)2++,	pt++, pt(NH3)++, Pt(NH3)2++	Pt++, Pt(NH3)++, Pt(NH3)2++,Pt+,	Pt++,
Spectrum filters	Threshold	0.01	0.001	0.01	0.001	
Spectrum filters	Mass accuracy	5	5	5	5	5
Spectrum filters	Minimum similarity	80	75	80	70	80
Spectrum filters	Zone	-1.5-5.5	-1.5-5.5	-1.5-5.5	-1.5-5.5	-1.5-5.5
MS/MS fragment types	Fragments	none	All	none	a, b, y, immonium ions	a,b,y
MS/MS fragment types	fragment losses		D-H2O and Z-CH2 not included		yes	No
MS/MS fragment types	Internal fragments		All		YA and YB	
Peak similarity parameters	Minimum similarity		90		90	90
Peak similarity parameters	Minimal number of peaks		10		10	10
Peak similarity parameters	Var width		width = 5.1428e-7 * mass ** 1.4637 (S1)<br>width = 4.7802e-7 * mass ** 1.4774 (S1c)		width = 5.52e-7 * mass ** 1.45	width = 4.7475e-7 * mass ** 1.4766
Peak similarity parameters	bottom	massError+width*3				
Peak similarity parameters	top	massError+width				

Supplementary figures and tables for Oligonucleotide-Cisplatin studies

A

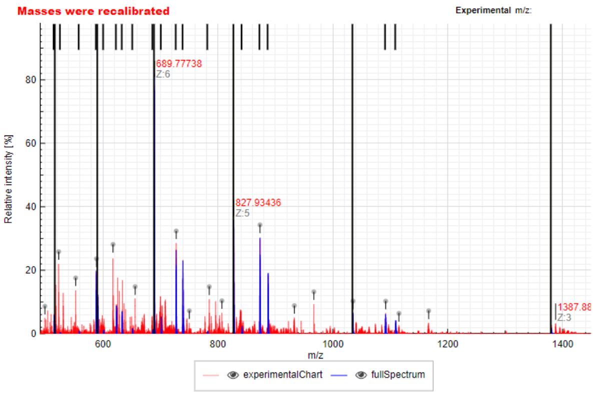

B

Strand	Variable group	MF	Ionization	MF Mass	m/z	ppm	Charge	Intensity	Similarity
S1	(Pt(NH3)2++)2	C128H173N53O77P12Pt2(+4)	(H+)-11	4445,7398	633,5225	-0,21	-7	5,3	0,82
S1	(Pt(NH3)2++)2	C128H173N53O77P12Pt2(+4)	(H+)-10	4445,7398	739,2775	0,00	-6	17,4	0,98
S1	(Pt(NH3)2++)2	C128H173N53O77P12Pt2(+4)	(H+)-9	4445,7398	887,3344	-0,01	-5	14,4	0,99
S1	(Pt(NH3)2++)2	C128H173N53O77P12Pt2(+4)	(H+)-8	4445,7398	1109,4198	0,14	-4	3,1	0,97
S1	(Pt(NH3)2++)2	C128H173N53O77P12Pt2(+4)	(H+)-7	4445,7398	1479,5622	-1,83	-3	0,9	0,90
S1	Pt(NH3)2++	C128H167N51O77P12Pt(+2)	(H+)-10	4216,7219	525,8310	0,87	-8	0,3	0,80
S1	Pt(NH3)2++	C128H167N51O77P12Pt(+2)	(H+)-9	4216,7219	601,0936	-0,27	-7	0,8	0,89
**S1***	**Pt(NH3)2++**	**C128H167N51O77P12Pt(+2)**	**(H+)-8**	**4216,7219**	**701,4438**	**-0,15**	**-6**	**3,6**	**0,98**
S1	Pt(NH3)2++	C128H167N51O77P12Pt(+2)	(H+)-7	4216,7219	841,9340	0,41	-5	1,6	0,94
S1c	(Pt(NH3)2++)2	C126H172N52O75P12Pt2(+4)	(H+)-11	4374,7391	623,3795	-0,11	-7	6,7	0,98
S1c	(Pt(NH3)2++)2	C126H172N52O75P12Pt2(+4)	(H+)-10	4374,7391	727,4440	-0,28	-6	20,2	0,99
S1c	(Pt(NH3)2++)2	C126H172N52O75P12Pt2(+4)	(H+)-9	4374,7391	873,1343	0,00	-5	23,2	0,99
S1c	(Pt(NH3)2++)2	C126H172N52O75P12Pt2(+4)	(H+)-8	4374,7391	1091,6697	0,27	-4	4,7	0,99
S1c	(Pt(NH3)2++)2	C126H172N52O75P12Pt2(+4)	(H+)-7	4374,7391	1455,8953	-0,44	-3	1,2	0,95
S1c	Pt(NH3)++	C126H163N49O75P12Pt(+2)	(H+)-10	4128,6946	514,8276	0,28	-8	4,0	0,97
S1c	Pt(NH3)++	C126H163N49O75P12Pt(+2)	(H+)-9	4128,6946	588,5183	0,17	-7	13,6	0,98
S1c	Pt(NH3)++	C126H163N49O75P12Pt(+2)	(H+)-8	4128,6946	686,7255	0,45	-6	0,9	0,89
S1c	Pt(NH3)2++	C126H166N50O75P12Pt(+2)	(H+)-10	4145,7212	516,9559	-0,10	-8	2,0	0,94
S1c	Pt(NH3)2++	C126H166N50O75P12Pt(+2)	(H+)-9	4145,7212	590,9507	-0,02	-7	8,5	0,98
**S1c***	**Pt(NH3)2++**	**C126H166N50O75P12Pt(+2)**	**(H+)-8**	**4145,7212**	**689,6103**	**0,18**	**-6**	**56,5**	**0,99**
S1c	Pt(NH3)2++	C126H166N50O75P12Pt(+2)	(H+)-7	4145,7212	827,7338	0,24	-5	24,7	0,99
S1c	Pt(NH3)2++	C126H166N50O75P12Pt(+2)	(H+)-6	4145,7212	1034,9191	0,05	-4	4,5	0,98
S1c	Pt(NH3)2++	C126H166N50O75P12Pt(+2)	(H+)-5	4145,7212	1380,2279	-0,15	-3	1,2	0,93
S1c		C126H160N48O75P12	(H+)-7	3916,7033	558,5218	-0,39	-7	0,6	0,94
S1c		C126H160N48O75P12	(H+)-6	3916,7033	651,7766	-0,14	-6	1,1	0,90
S1c		C126H160N48O75P12	(H+)-5	3916,7033	782,3334	-0,41	-5	0,5	0,87

Figure S5. Oligonucleotide binding study to cisplatin: Aom^{2}s results from analysis of the full scan HRMS spectrum. A) overlay of experimental (ref) and assigned theoretical (blue) mass spectra after recalibration B) List of fragments assigned by the tool for each strand S1 and S1c. *: ions selected for further CID fragmentation.

Table S2. List of CID fragments assigned by Aom^{2}s for the parent ion $[S1+Pt(NH_3)_2-8H]^{6-}$ (*m/z* 701.4438) including fragment type, molecular formula (MF), theoretical and experimental *m/z*, error (in ppm), intensity (expressed as percentage of the base peak in the spectra) and similarity scores in percentage.

Type	MF	Ionization	Variable group	MF Mass	Th m/z	Exp m/z	ppm	Charge	Intensity (%)	Similarity
w6:a9-B	C14H19N3O11P2	(H+)-1		467.0495	466.0422	466.0423	0.11	-1	37.79	0.966
w4:a11-B	C14H19N3O11P2	(H+)-1		467.0495	466.0422	466.0423	0.11	-1	37.79	0.966
x2-B2	C15H20N2O12P2	(H+)-1		482.0491	481.0419	481.0419	-0.02	-1	51.57	0.963
w12:a3-B	C15H20N2O12P2	(H+)-1		482.0491	481.0419	481.0419	-0.02	-1	51.57	0.963
w10:a5-B	C15H20N2O12P2	(H+)-1		482.0491	481.0419	481.0419	-0.02	-1	51.57	0.963
w9:a6-B	C15H20N2O12P2	(H+)-1		482.0491	481.0419	481.0419	-0.02	-1	51.57	0.963
a6-B	C55H69N16O34P5	(H+)-2		1652.2850	825.1352	825.1361	1.01	-2	25.16	0.937
a6-B5	C55H69N16O34P5	(H+)-2		1652.2850	825.1352	825.1361	1.01	-2	25.16	0.937
y12:a7-B	C55H69N16O34P5	(H+)-2		1652.2850	825.1352	825.1361	1.01	-2	25.16	0.937
y6:a10-B	C24H30N8O13P2	(H+)-1		700.1408	699.1335	699.1335	0.01	-1	46.68	0.929
y5:a11-B	C24H30N8O13P2	(H+)-1		700.1408	699.1335	699.1335	0.01	-1	46.68	0.929
w4	C39H51N15O25P4	(H+)-2		1253.2131	625.5993	625.5992	-0.17	-2	56.42	0.929
w5:b12	C39H51N15O25P4	(H+)-2		1253.2131	625.5993	625.5992	-0.17	-2	56.42	0.929
w1	C10H14N5O6P	(H+)-1		331.0682	330.0609	330.0600	-2.72	-1	6.18	0.921
w7:a9-B	C24H31N8O17P3	(H+)-1		796.1020	795.0947	795.0948	0.11	-1	11.84	0.912
w4:a12-B	C24H31N8O17P3	(H+)-1		796.1020	795.0947	795.0948	0.11	-1	11.84	0.912
w7	C68H87N28O42P7	(H+)-3		2184.3696	727.1159	727.1157	-0.29	-3	14.06	0.897
a3-B	C25H31N7O15P2	(H+)-1		731.1353	730.1281	730.1279	-0.20	-1	75.32	0.897
a3-B2	C25H31N7O15P2	(H+)-1		731.1353	730.1281	730.1279	-0.20	-1	75.32	0.897
y9:a7-B	C25H31N7O15P2	(H+)-1		731.1353	730.1281	730.1279	-0.20	-1	75.32	0.897
w6:a11-B	C33H43N11O22P4	(H+)-2		1069.1535	533.5695	533.5693	-0.33	-2	5.11	0.896
x6-B4	C53H68N18O34P6	(H+)-2		1686.2571	842.1213	842.1219	0.68	-2	8.92	0.893
w12:a5-B	C35H45N9O24P4	(H+)-2		1099.1528	548.5691	548.5690	-0.22	-2	3.01	0.882
w11:a6-B	C35H45N9O24P4	(H+)-2		1099.1528	548.5691	548.5690	-0.22	-2	3.01	0.882
x1	C10H12N5O5P	(H+)-1		313.0576	312.0503	312.0499	-1.51	-1	1.17	0.870
w4	C39H51N15O25P4	(H+)-3		1253.2131	416.7304	416.7300	-1.07	-3	12.56	0.870
w5:b12	C39H51N15O25P4	(H+)-3		1253.2131	416.7304	416.7300	-1.07	-3	12.56	0.870
a7-B	C65H81N21O40P6	(H+)-3		1981.3375	659.4386	659.4387	0.23	-3	3.56	0.867
a7-B5	C65H81N21O40P6	(H+)-3		1981.3375	659.4386	659.4387	0.23	-3	3.56	0.867
a7-B6	C65H81N21O40P6	(H+)-3		1981.3375	659.4386	659.4387	0.23	-3	3.56	0.867
y12:a8-B	C65H81N21O40P6	(H+)-3		1981.3375	659.4386	659.4387	0.23	-3	3.56	0.867
b3	C30H38N12O16P2	(H+)-1		884.2004	883.1931	883.1937	0.65	-1	2.66	0.866
y3	C30H38N12O16P2	(H+)-1		884.2004	883.1931	883.1937	0.65	-1	2.66	0.866
w5	C49H63N20O30P5	(H+)-3		1566.2707	521.0830	521.0827	-0.57	-3	13.97	0.863
w6	C58H75N23O36P6	(H+)-3		1855.3171	617.4317	617.4316	-0.24	-3	18.76	0.861
w10:a6-B	C25H33N4O19P3	(H+)-1		786.0952	785.0879	785.0875	-0.53	-1	28.42	0.860
y6	C58H74N23O33P5	(H+)-2		1775.3507	886.6681	886.6696	1.74	-2	6.21	0.858
a5-B	C45H56N14O27P4	(H+)-2		1348.2390	673.1122	673.1126	0.51	-2	0.99	0.846
a5-B1	C45H56N14O27P4	(H+)-2		1348.2390	673.1122	673.1126	0.51	-2	0.99	0.846
a5-B3	C45H56N14O27P4	(H+)-2		1348.2390	673.1122	673.1126	0.51	-2	0.99	0.846
a5-B4	C45H56N14O27P4	(H+)-2		1348.2390	673.1122	673.1126	0.51	-2	0.99	0.846
y11:a7-B	C45H56N14O27P4	(H+)-2		1348.2390	673.1122	673.1126	0.51	-2	0.99	0.846
w6:a10-B	C24H31N8O16P3	(H+)-1		780.1071	779.0998	779.1003	0.64	-1	10.08	0.846

Type	MF	Ionization	variable group	MF Mass	Th m/z	Exp m/z	ppm	Charge	Intensity %	Similarity
w5:a11-B	C24H31N8O16P3	(H+)-1		780.1071	779.0998	779.1003	0.64	-1	10.08	0.846
w6:a9	C19H24N8O11P2	(H+)-1		602.1040	601.0967	601.0969	0.36	-1	12.71	0.844
w5-B3	C44H58N15O29P5	(H+)-3		1415.2213	470.7332	470.7324	-1.53	-3	4.55	0.842
w2	C20H27N7O13P2	(H+)-1		635.1142	634.1069	634.1069	-0.04	-1	17.91	0.837
w12:b3	C20H27N7O13P2	(H+)-1		635.1142	634.1069	634.1069	-0.04	-1	17.91	0.837
w11:b4	C20H27N7O13P2	(H+)-1		635.1142	634.1069	634.1069	-0.04	-1	17.91	0.837
w2	C20H30N8o13P2Pt(+2)	(H+)-1	Pt(NH3)++	847.1055	846.0972	846.0975	0.35	-1	3.02	0.830
w12:b3	C20H30N8O13P2Pt(+2)	(H+)-1	Pt(NH3)++	847.1055	847.1055	846.0975	0.35	-1	3.02	0.830
w11:b4	C20H30N8O13P2Pt(+2)	(H+)-1	Pt(NH3)++	847.1055	847.1055	847.0975	0.35	-1	3.02	0.830
w6:b12	C48H63N18O31P5	(H+)-2		1542.2595	770.1225	770.1234	1.19	-2	1.79	0.826
y12:a3-B	C15H19N2O9P	(H+)-1		402.0828	401.0755	401.0750	-1.44	-1	9.79	0.825
y10:a5-B	C15H19N2O9P	(H+)-1		402.0828	401.0755	401.0750	-1.44	-1	9.79	0.825
y9:a6-B	C15H19N2O9P	(H+)-1		402.0828	401.0755	401.0750	-1.44	-1	9.79	0.825
w8:b10	C48H62N21O30P5	(H+)-3		1567.2660	521.4147	521.4168	3.94	-3	5.34	0.820
w7:b11	C48H62N21O30P5	(H+)-3		1567.2660	521.4147	521.4168	3.94	-3	5.34	0.820
a2-B	C15H18N5O8P	(H+)-1		427.0893	426.0820	426.0812	-1.83	-1	3.76	0.818
a2-B1	C15H18N5O8P	(H+)-1		427.0893	426.0820	426.0812	-1.83	-1	3.76	0.818
y8:a7-B	C15H18N5O8P	(H+)-1		427.0893	426.0820	426.0812	-1.83	-1	3.76	0.818
y7:a8-B	C15H18N5O8P	(H+)-1		427.0893	426.0820	426.0812	-1.83	-1	3.76	0.818
y3:a12-B	C15H18N5O8P	(H+)-1		427.0893	426.0820	426.0812	-1.83	-1	3.76	0.818
a9-B	C84H108N30O52P8Pt(+2)	(H+)-5	Pt(NH3)++	2811.4278	935.4634	935.4645	1.18	-3	4.64	0.815
a9-B2	C84H108N30O52P8Pt(+2)	(H+)-5	Pt(NH3)++	2811.4278	935.4634	935.4645	1.18	-3	4.64	0.815
b9-B5	C84H108N30O52P8Pt(+2)	(H+)-5	Pt(NH3)++	2811.4278	935.4634	935.4645	1.18	-3	4.64	0.815
b9-B6	C84H108N30O52P8Pt(+2)	(H+)-5	Pt(NH3)++	2811.4278	935.4634	935.4645	1.18	-3	4.64	0.815
a9-B8	C84H108N30O52P8Pt(+2)	(H+)-5	Pt(NH3)++	2811.4278	935.4634	935.4645	1.18	-3	4.64	0.815
w6:a11-B	C33H43N11O22P4	(H+)-1		1069.1535	1068.1462	1068.1473	1.01	-3	3.27	0.813
w10:a5	C20H26N4O14P2	(H+)-2		608.0921	303.0388	303.0381	-2.33	-3	7.35	0.809
y4	C39H50N15O22P3	(H+)-1		1173.2468	1172.2395	1172.2415	1.74	-3	4.17	0.807
w5	C49H63N20O30P5	(H+)-2		1566.2707	782.1281	782.1289	1.05	-3	7.28	0.804
w6	C58H75N23O36P6	(H+)-2		1855.3171	926.6513	926.6519	0.67	-3	7.89	0.802
x12-B5	C113H143N39O72P12Pt(+2)	(H+)-6	(Pt++)	3764.5227	939.6195	939.6227	3.36	-3	3.82	0.801
x12-B6	C113H143N39O72P12Pt(+2)	(H+)-6	(Pt++)	3764.5227	939.6195	939.6227	3.36	-3	3.82	0.801
x12-B10	C113H143N39O72P12Pt(+2)	(H+)-6	(Pt++)	3764.5227	939.6195	939.6227	3.36	-3	3.82	0.801
c10	C98H129N39O61P10Pt(+2)	(H+)-7	Pt(NH3)2++	3332.5215	665.0939	665.0908	-4.66	-3	6.45	0.792
x10	C98H129N39O61P10Pt(+2)	(H+)-7	Pt(NH3)2++	3332.5215	665.0939	665.0908	-4.66	-3	6.45	0.792
a7-B	C65H87N23O40P6Pt(+2)	(H+)-5	Pt(NH3)2++	2210.3554	735.1060	735.1085	3.34	-3	2.18	0.783
a7-B5	C65H87N23O40P6Pt(+2)	(H+)-5	Pt(NH3)2++	2210.3554	735.1060	735.1085	3.34	-3	2.18	0.783
a7-B6	C65H87N23O40P6Pt(+2)	(H+)-5	Pt(NH3)2++	2210.3554	735.1060	735.1085	5.43	-3	2.04	0.774
z4-B2	C34H44N10O20P3(+1)	(H+)-3		1005.1946	501.0861	501.0865	0.69	-2	19.19	0.774
w6	C58H75N23O36P6	(H+)-3		1855.3171	617.4317	617.4316	-0.24	-3	18.76	0.773
z4-B2	C34H44N10O20P3(+1)	(H+)-2		1005.1946	1003.1795	1003.1784	-1.09	-1	23.59	0.773
z3-B3	C25H32N7O15P2(+1)	(H+)-2		732.1432	730.1281	730.1277	-0.52	-1	75.32	0.773
w10:a5	C20H26N4O14P2	(H+)-2		608.0921	303.0388	303.0395	-2.33		7.35	0.772
c8-B	C74H97N25O49P8Pt(+2)	(H+)-5		2562.3416	852.4347	852.4363	1.84	-3	0.97	0.772
z2-B2	C15H20N2O9P(+1)	(H+)-2		403.0906	401.0755	401.0755	0.00	-1	9.79	0.772
c4-B3	C35H45N9O25P4	(H+)-1		1115.1477	1114.1404	1114.1399	-0.48	-1	4.05	0.770
d3	C30H39N12O19P3	(H+)-2		964.1667	481.0761	481.0759	-0.44	-2	5.17	0.770
w3	C30H39N12O19P3	(H+)-2		964.1667	481.0761	481.0759	-0.44	-2	5.17	0.770
y9:a10-B	C54H70N21O32P5Pt(+2)	(H+)-4	Pt(NH3)++	1874.2832	935.1265	935.1316	5.43	-3	2.04	0.770
a9-B	C84H111N31O52P8Pt(+2)	(H+)-5	Pt(NH3)2++	2828.4543	941.1389	941.1410	2.25	-3	1.77	0.764
a9-B2	C84H111N31O52P8Pt(+2)	(H+)-5	Pt(NH3)2++	2828.4543	941.1389	941.1410	2.25	-3	1.77	0.764
b9-B5	C84H111N31O52P8Pt(+2)	(H+)-5	Pt(NH3)2++	2828.4543	941.1389	941.1410	2.25	-3	1.77	0.764
b9-B6	C84H111N31O52P8Pt(+2)	(H+)-5	Pt(NH3)2++	2828.4543	941.1389	941.1410	2.25	-3	1.77	0.764
a9-B8	C84H111N31O52P8Pt(+2)	(H+)-5	Pt(NH3)2++	2828.4543	941.1389	941.1410	2.25	-3	1.77	0.764
a7 -B	C65H81N21O40P6	(H+)-2		1981.3375	989.6615	989.6616	0.15	-2	3.98	0.763
y4	C39H50N15O22P3	(H+)-2		1173.2468	585.6161	585.6164	0.57	-2	2.91	0.760
w2	C20H27N7O13P2	(H+)-2		635.1142	316.5498	316.5498	-0.03	-2	2.43	0.760

Table S3. List of all platinated CID fragments assigned by Aom²s for the parent ion $[S1+Pt(NH_3)_2-8H]^{6-}$ (*m/z* 701.4438) including fragment type, molecular formula (MF), theoretical and experimental *m/z*, error (in ppm), intensity (expressed as percentage of the base peak in the spectra) and similarity scores in percentage.

Type	MF	Ionization	Variable group	MF Mass	Th m/z	ppm	Charge	Intensity (%)	Similarity
w2	C20H30N8O13P2Pt(+2)	(H+)-1	Pt(NH3)++	847.1055	846.0972	0.35	1	3.02	0.83
w12:b3	C20H30N8O13P2Pt(+2)	(H+)-1	Pt(NH3)++	847.1055	846.0972	0.35	1	3.02	0.83
w11:b4	C20H30N8O13P2Pt(+2)	(H+)-1	Pt(NH3)++	847.1055	846.0972	0.35	1	3.02	0.83
a9-B	C84H108N30O52P8Pt(+2)	(H+)-5	Pt(NH3)++	2811.4278	935.4634	1.18	-3	4.64	0.81
a9-B2	C84H108N30O52P8Pt(+2)	(H+)-5	Pt(NH3)++	2811.4278	935.4634	1.18	-3	4.64	0.81
b9-B5	C84H108N30O52P8Pt(+2)	(H+)-5	Pt(NH3)++	2811.4278	935.4634	1.18	-3	4.64	0.81
b9-B6	C84H108N30O52P8Pt(+2)	(H+)-5	Pt(NH3)++	2811.4278	935.4634	1.18	-3	4.64	0.81
a9-B8	C84H108N30O52P8Pt(+2)	(H+)-5	Pt(NH3)++	2811.4278	935.4634	1.18	-3	4.64	0.81
x12-B5	C113H143N39O72P12Pt(+2)	(H+)-7	(Pt++)	3764.5227	751.4941	3.36	-5	3.82	0.80
x12-B6	C113H143N39O72P12Pt(+2)	(H+)-7	(Pt++)	3764.5227	751.4941	3.36	-5	3.82	0.80
x12-B10	C113H143N39O72P12Pt(+2)	(H+)-7	(Pt++)	3764.5227	751.4941	3.36	-5	3.82	0.80
c10	C98H129N39O61P10Pt(+2)	(H+)-7	Pt(NH3)2++	3332.5215	665.0939	-4.66	-5	6.45	0.79
x10	C98H129N39O61P10Pt(+2)	(H+)-7	Pt(NH3)2++	3332.5215	665.0939	-4.66	-5	6.45	0.79
a7-B	C65H87N23O40P6Pt(+2)	(H+)-5	Pt(NH3)2++	2210.3554	735.1060	3.34	-3	2.18	0.78
a7-B5	C65H87N23O40P6Pt(+2)	(H+)-5	Pt(NH3)2++	2210.3554	735.1060	3.34	-3	2.18	0.78
a7-B6	C65H87N23O40P6Pt(+2)	(H+)-5	Pt(NH3)2++	2210.3554	735.1060	3.34	-3	2.18	0.77
y9:a10-B	C54H70N21O32P5Pt(+2)	(H+)-4	Pt(NH3)++	1874.2832	935.1265	5.43	-2	2.04	0.77
a9-B	C84H111N31O52P8Pt(+2)	(H+)-5	Pt(NH3)2++	2828.4543	941.1389	2.25	-3	1.77	0.76
a9-B2	C84H111N31O52P8Pt(+2)	(H+)-5	Pt(NH3)2++	2828.4543	941.1389	2.25	-3	1.77	0.76
b9-B5	C84H111N31O52P8Pt(+2)	(H+)-5	Pt(NH3)2++	2828.4543	941.1389	2.25	-3	1.77	0.76
b9-B6	C84H111N31O52P8Pt(+2)	(H+)-5	Pt(NH3)2++	2828.4543	941.1389	2.25	-3	1.77	0.76
a9-B8	C84H111N31O52P8Pt(+2)	(H+)-5	Pt(NH3)2++	2828.4543	941.1389	2.25	-3	1.77	0.76

Table S4. List of CID fragments assigned by Aom[2]s for the parent ion $[S1c+Pt(NH_3)_2\text{-}8H]^{6-}$ (*m/z* 689.6103) including fragment type, molecular formula (MF), theoretical and experimental *m/z*, error (in ppm), intensity (expressed as percentage of the base peak in the spectra) and similarity scores in percentage.

Type	MF	Ionization	Variable group	MF Mass	Th m/z	Exp m/z	ppm	Charge	Intensity (%)	Similarity
w1	C9H14N3O7P	(H+)-1		307.0569	306.0497	306.0495	-0.56	-1	48.9	0.98
a6	C59H79N24O34P5Pt(+2)	(H+)-5	Pt(NH3)2++	2017.3527	670.7717	670.7721	0.51	-3	20.0	0.95
a12-B	C112H144N41O68P11Pt(+2)	(H+)-6	Pt(NH3)2++	3686.5832	920.1346	920.1346	0.00	-4	6.6	0.95
a12-B1	C112H144N41O68P11Pt(+2)	(H+)-6	Pt(NH3)2++	3686.5832	920.1346	920.1346	0.00	-4	6.6	0.95
b12-B3	C112H144N41O68P11Pt(+2)	(H+)-6	Pt(NH3)2++	3686.5832	920.1346	920.1346	0.00	-4	6.6	0.95
b12-B5	C112H144N41O68P11Pt(+2)	(H+)-6	Pt(NH3)2++	3686.5832	920.1346	920.1346	0.00	-4	6.6	0.95
a12-B8	C112H144N41O68P11Pt(+2)	(H+)-6	Pt(NH3)2++	3686.5832	920.1346	920.1346	0.00	-4	6.6	0.95
a12-B9	C112H144N41O68P11Pt(+2)	(H+)-6	Pt(NH3)2++	3686.5832	920.1346	920.1346	0.00	-4	6.6	0.95
a12-B11	C112H144N41O68P11Pt(+2)	(H+)-6	Pt(NH3)2++	3686.5832	920.1346	920.1346	0.00	-4	6.6	0.95
a12-B	C112H147N42O68P11Pt(+2)	(H+)-6	Pt(NH3)2++	3703.6098	924.3912	924.3916	0.33	-4	15.8	0.95
a12-B1	C112H147N42O68P11Pt(+2)	(H+)-6	Pt(NH3)2++	3703.6098	924.3912	924.3916	0.33	-4	15.8	0.95
b12-B3	C112H147N42O68P11Pt(+2)	(H+)-6	Pt(NH3)2++	3703.6098	924.3912	924.3916	0.33	-4	15.8	0.95
b12-B5	C112H147N42O68P11Pt(+2)	(H+)-6	Pt(NH3)2++	3703.6098	924.3912	924.3916	0.33	-4	15.8	0.95
a12-B8	C112H147N42O68P11Pt(+2)	(H+)-6	Pt(NH3)2++	3703.6098	924.3912	924.3916	0.33	-4	15.8	0.95
a12-B9	C112H147N42O68P11Pt(+2)	(H+)-6	Pt(NH3)2++	3703.6098	924.3912	924.3916	0.33	-4	15.8	0.95
a12-B11	C112H147N42O68P11Pt(+2)	(H+)-6	Pt(NH3)2++	3703.6098	924.3912	924.3916	0.33	-4	15.8	0.95
w7	C67H87N26O41P7	(H+)-4		2128.3685	531.0849	531.0852	0.65	-4	6.7	0.94
x6-B2	C53H68N18O34P6	(H+)-2		1686.2571	842.1213	842.1212	-0.05	-2	31.1	0.94
x6-B3	C53H68N18O34P6	(H+)-2		1686.2571	842.1213	842.1212	-0.05	-2	31.1	0.94
x6-B5	C53H68N18O34P6	(H+)-2		1686.2571	842.1213	842.1212	-0.05	-2	31.1	0.94
w7:a12-B	C53H68N18O34P6	(H+)-2		1686.2571	842.1213	842.1212	-0.05	-2	31.1	0.94
a6	C59H76N23O34P5Pt(+2)	(H+)-4	Pt(NH3)++	2000.3261	998.1480	998.1484	0.44	-2	8.6	0.94
a4-B	C34H43N10O20P3	(H+)-2		1004.1868	501.0861	501.0864	0.54	-2	9.0	0.93
a4-B3	C34H43N10O20P3	(H+)-2		1004.1868	501.0861	501.0864	0.54	-2	9.0	0.93
a10-B	C92H122N35O56P9Pt(+2)	(H+)-6	Pt(NH3)2++	3086.5061	770.1153	770.1152	-0.15	-4	5.6	0.93
a10-B1	C92H122N35O56P9Pt(+2)	(H+)-6	Pt(NH3)2++	3086.5061	770.1153	770.1152	-0.15	-4	5.6	0.93
b10-B3	C92H122N35O56P9Pt(+2)	(H+)-6	Pt(NH3)2++	3086.5061	770.1153	770.1152	-0.15	-4	5.6	0.93
b10-B5	C92H122N35O56P9Pt(+2)	(H+)-6	Pt(NH3)2++	3086.5061	770.1153	770.1152	-0.15	-4	5.6	0.93
a10-B8	C92H122N35O56P9Pt(+2)	(H+)-6	Pt(NH3)2++	3086.5061	770.1153	770.1152	-0.15	-4	5.6	0.93
a10-B9	C92H122N35O56P9Pt(+2)	(H+)-6	Pt(NH3)2++	3086.5061	770.1153	770.1152	-0.15	-4	5.6	0.93
y11:a12-B	C92H122N35O56P9Pt(+2)	(H+)-6	Pt(NH3)2++	3086.5061	770.1153	770.1152	-0.15	-4	5.6	0.93
d3-B1	C24H34N5O19P3	(H+)-1		789.1061	788.0988	788.0988	-0.06	-1	2.6	0.93
w3-B2	C24H34N5O19P3	(H+)-1		789.1061	788.0988	788.0988	-0.06	-1	2.6	0.93
c2-B1	C15H20N2O12P2	(H+)-1		482.0491	481.0419	481.0423	0.91	-1	12.1	0.92
w9:a6-B	C15H20N2O12P2	(H+)-1		482.0491	481.0419	481.0423	0.91	-1	12.1	0.92
w3:a12-B	C15H20N2O12P2	(H+)-1		482.0491	481.0419	481.0423	0.91	-1	12.1	0.92
a6-B	C54H68N17O33P5	(H+)-2		1637.2854	817.6354	817.6349	-0.64	-2	2.9	0.92
a6-B3	C54H68N17O33P5	(H+)-2		1637.2854	817.6354	817.6349	-0.64	-2	2.9	0.92
a6-B5	C54H68N17O33P5	(H+)-2		1637.2854	817.6354	817.6349	-0.64	-2	2.9	0.92
x2-B1	C14H19N3O11P2	(H+)-1		467.0495	466.0422	466.0425	0.62	-1	5.4	0.91
w11:a4-B	C14H19N3O11P2	(H+)-1		467.0495	466.0422	466.0425	0.62	-1	5.4	0.91
w7:a8-B	C14H19N3O11P2	(H+)-1		467.0495	466.0422	466.0425	0.62	-1	5.4	0.91
w6:a9-B	C14H19N3O11P2	(H+)-1		467.0495	466.0422	466.0425	0.62	-1	5.4	0.91
w7	C67H87N26O41P7	(H+)-3		2128.3685	708.4489	708.4491	0.27	-3	23.7	0.91

Type	MF	Ionization	Variable group	MF Mass	Th m/z	Exp m/z	ppm	Charge	Intensity (%)	Similarity
y7	C67H86N26O38P6	(H+)-2		2048.4022	1023.1938	1023.1955	1.65	-2	1.0	0.91
w7:a10-B	C33H43N11O22P4	(H+)-2		1069.1535	533.5695	533.5698	0.73	-2	6.5	0.91
x7-B1	C63H80N23O39P7	(H+)-2		1999.3147	998.6501	998.6482	-1.88	-2	9.4	0.90
x7-B2	C63H80N23O39P7	(H+)-2		1999.3147	998.6501	998.6482	-1.88	-2	9.4	0.90
x7-B7	C63H80N23O39P7	(H+)-2		1999.3147	998.6501	998.6482	-1.88	-2	9.4	0.90
w9:a12-B	C73H93N25O47P8	(H+)-3		2319.3557	772.1113	772.1113	0.01	-3	5.8	0.90
a6	C59H79N24O34P5Pt(+2)	(H+)-4	Pt(NH3)2++	2017.3527	1006.6612	1006.6610	-0.18	-2	19.2	0.89
w9:a9-B	C43H56N13O30P5	(H+)-2		1389.1944	693.5899	693.5902	0.41	-2	3.3	0.89
w6	C58H75N23O35P6	(H+)-3		1839.3222	612.1001	612.1000	-0.21	-3	1.4	0.89
w7:b12	C58H75N23O35P6	(H+)-3		1839.3222	612.1001	612.1000	-0.21	-3	1.4	0.89
b3	C29H38N10O16P2	(H+)-1		844.1942	843.1870	843.1874	0.46	-1	1.7	0.89
y3	C29H38N10O16P2	(H+)-1		844.1942	843.1870	843.1874	0.46	-1	1.7	0.89
d3-B1	C24H34N5O19P3	(H+)-2		789.1061	393.5458	393.5458	0.06	-2	6.7	0.89
w3-B2	C24H34N5O19P3	(H+)-2		789.1061	393.5458	393.5458	0.06	-2	6.7	0.89
a10-B	C92H122N35O56P9Pt(+2)	(H+)-5	Pt(NH3)2++	3086.5061	1027.1562	1027.1565	0.29	-3	7.6	0.89
a10-B1	C92H122N35O56P9Pt(+2)	(H+)-5	Pt(NH3)2++	3086.5061	1027.1562	1027.1565	0.29	-3	7.6	0.89
b10-B3	C92H122N35O56P9Pt(+2)	(H+)-5	Pt(NH3)2++	3086.5061	1027.1562	1027.1565	0.29	-3	7.6	0.89
b10-B5	C92H122N35O56P9Pt(+2)	(H+)-5	Pt(NH3)2++	3086.5061	1027.1562	1027.1565	0.29	-3	7.6	0.89
a10-B8	C92H122N35O56P9Pt(+2)	(H+)-5	Pt(NH3)2++	3086.5061	1027.1562	1027.1565	0.29	-3	7.6	0.89
a10-B9	C92H122N35O56P9Pt(+2)	(H+)-5	Pt(NH3)2++	3086.5061	1027.1562	1027.1565	0.29	-3	7.6	0.89
y11:a12-B	C92H122N35O56P9Pt(+2)	(H+)-5	Pt(NH3)2++	3086.5061	1027.1562	1027.1565	0.29	-3	7.6	0.89
	C92H120B3N34O55P9Pt(+2)	(H+)-6	Pt(NH3)++	3087.5204	770.3689	770.3660	-3.83	-4	6.5	0.88
a10-B	C92H119N34O56P9Pt(+2)	(H+)-6	Pt(NH3)++	3069.4796	765.8587	765.8591	0.47	-4	3.4	0.88
a10-B1	C92H119N34O56P9Pt(+2)	(H+)-6	Pt(NH3)++	3069.4796	765.8587	765.8591	0.47	-4	3.4	0.88
b10-B3	C92H119N34O56P9Pt(+2)	(H+)-6	Pt(NH3)++	3069.4796	765.8587	765.8591	0.47	-4	3.4	0.88
b10-B5	C92H119N34O56P9Pt(+2)	(H+)-6	Pt(NH3)++	3069.4796	765.8587	765.8591	0.47	-4	3.4	0.88
a10-B8	C92H119N34O56P9Pt(+2)	(H+)-6	Pt(NH3)++	3069.4796	765.8587	765.8591	0.47	-4	3.4	0.88
a10-B9	C92H119N34O56P9Pt(+2)	(H+)-6	Pt(NH3)++	3069.4796	765.8587	765.8591	0.47	-4	3.4	0.88
y11:a12-B	C92H119N34O56P9Pt(+2)	(H+)-6	Pt(NH3)++	3069.4796	765.8587	765.8591	0.47	-4	3.4	0.88
d3	C29H39N10O19P3	(H+)-2		924.1606	461.0730	461.0732	0.47	-2	21.4	0.88
w3	C29H39N10O19P3	(H+)-2		924.1606	461.0730	461.0732	0.47	-2	21.4	0.88
w7:a10-B	C33H43N11O22P4	(H+)-1		1069.1535	1068.1462	1068.1461	-0.10	-1	3.9	0.87
x6-B2	C53H68N18O34P6	(H+)-3		1686.2571	561.0784	561.0782	-0.35	-3	2.3	0.86
x6-B3	C53H68N18O34P6	(H+)-3		1686.2571	561.0784	561.0782	-0.35	-3	2.3	0.86
x6-B5	C53H68N18O34P6	(H+)-3		1686.2571	561.0784	561.0782	-0.35	-3	2.3	0.86
w7:a12-B	C53H68N18O34P6	(H+)-3		1686.2571	561.0784	561.0782	-0.35	-3	2.3	0.86
a6-B	C54H74N19O33P5Pt(+2)	(H+)-4	Pt(NH3)2++	1866.3032	931.1365	931.1376	1.12	-2	3.0	0.85
a6-B3	C54H74N19O33P5Pt(+2)	(H+)-4	Pt(NH3)2++	1866.3032	931.1365	931.1376	1.12	-2	3.0	0.85
a6-B5	C54H74N19O33P5Pt(+2)	(H+)-4	Pt(NH3)2++	1866.3032	931.1365	931.1376	1.12	-2	3.0	0.85
a8-B	C73H95N26O45P7Pt(+2)	(H+)-5	Pt(NH3)++	2467.3756	820.7794	820.7787	-0.86	-3	1.0	0.85
a8-B2	C73H95N26O45P7Pt(+2)	(H+)-5	Pt(NH3)++	2467.3756	820.7794	820.7787	-0.86	-3	1.0	0.85
a8-B6	C73H95N26O45P7Pt(+2)	(H+)-5	Pt(NH3)++	2467.3756	820.7794	820.7787	-0.86	-3	1.0	0.85
a8-B7	C73H95N26O45P7Pt(+2)	(H+)-5	Pt(NH3)++	2467.3756	820.7794	820.7787	-0.86	-3	1.0	0.85

Type	MF	Ionization	variable group	MF Mass	Th m/z	Ex m/z	ppm	Charge	Intensity(%)	Similarity
w4	C39H51N15O24P4	(H+)-2		1237.2182	617.6018	617.6021	0.42	-2	10.71	0.85
w6:b11	C39H51N15O24P4	(H+)-2		1237.2182	617.6018	617.6021	0.42	-2	10.71	0.85
w7:a9-B	C23H31N6O17P3	(H+)-1		756.0959	755.0886	755.0890	0.50	-1	2.86	0.85
a11	C107H140N42O63P10Pt(+2)	(H+)-6	Pt(NH3)2++	3525.6066	879.8905	879.8915	1.12	-4	1.30	0.84
w9:a10-B	C53H68N18O35P6	(H+)-2		1702.2520	850.1187	850.1190	0.26	-2	2.96	0.83
	C92H120B3N34O55P9Pt(+2)	(H+)-4	Pt(NH3)++	3087.5204	1027.4943	1027.4894	-4.80	-3	4.90	0.83
x7-B5	C62H79N24O38P7	(H+)-2		1984.3150	991.1502	991.1542	3.96	-2	5.99	0.82
a2-B	C15H19N2O9P	(H+)-1		402.0828	401.0755	401.0762	1.68	-1	2.32	0.82
a2-B1	C15H19N2O9P	(H+)-1		402.0828	401.0755	401.0762	1.68	-1	2.32	0.82
y9:a6-B	C15H19N2O9P	(H+)-1		402.0828	401.0755	401.0762	1.68	-1	2.32	0.82
y3:a12-B	C15H19N2O9P	(H+)-1		402.0828	401.0755	401.0762	1.68	-1	2.32	0.82
w2-B1	C14H21N3O12P2	(H+)-2		485.0600	241.5227	241.5229	0.69	-2	0.44	0.82
x5-B1	C44H56N15O28P5	(H+)-2		1397.2107	697.5981	697.5982	0.20	-2	3.30	0.82
x5-B2	C44H56N15O28P5	(H+)-2		1397.2107	697.5981	697.5982	0.20	-2	3.30	0.82
x5-B4	C44H56N15O28P5	(H+)-2		1397.2107	697.5981	697.5982	0.20	-2	3.30	0.82
w6:a12-B	C44H56N15O28P5	(H+)-2		1397.2107	697.5981	697.5982	0.20	-2	3.30	0.82
x4-B4	C35H44N12O22P4	(H+)-2		1108.1644	553.0749	553.0753	0.77	-2	1.08	0.82
w5:a12-B	C35H44N12O22P4	(H+)-2		1108.1644	553.0749	553.0753	0.77	-2	1.08	0.82
a3-B	C25H31N7O14P2	(H+)-1		715.1404	714.1331	714.1331	1.40	-1	1.45	0.82
a3-B2	C25H31N7O14P2	(H+)-1		715.1404	714.1331	714.1331	1.40	-1	1.45	0.82
y4:a12-B	C25H31N7O14P2	(H+)-1		715.1404	714.1331	714.1331	1.40	-1	1.45	0.82
	C92H117B3N33O55P9Pt(+2)	(H+)-6	(Pt++)	3070.4938	766.1123	766.1091	-4.20	-4	3.30	0.82
d4-B3	C34H46N10O24P4	(H+)-2		1102.1637	550.0746	550.0744	-0.37	-2	0.57	0.82
w4-B1	C34H46N10O24P4	(H+)-2		1102.1637	550.0746	550.0744	-0.37	-2	0.57	0.82
w4-B3	C34H46N10O24P4	(H+)-2		1102.1637	550.0746	550.0744	-0.37	-2	0.57	0.82
a9-B	C82H107N29O51P8Pt(+2)	(H+)-5	Pt(NH3)++	2756.4220	917.1282	917.1286	0.46	-3	1.58	0.82
a9-B1	C82H107N29O51P8Pt(+2)	(H+)-5	Pt(NH3)++	2756.4220	917.1282	917.1286	0.46	-3	1.58	0.82
b9-B3	C82H107N29O51P8Pt(+2)	(H+)-5	Pt(NH3)++	2756.4220	917.1282	917.1286	0.46	-3	1.58	0.82
b9-B5	C82H107N29O51P8Pt(+2)	(H+)-5	Pt(NH3)++	2756.4220	917.1282	917.1286	0.46	-3	1.58	0.82
a9-B8	C82H107N29O51P8Pt(+2)	(H+)-5	Pt(NH3)++	2756.4220	917.1282	917.1286	0.46	-3	1.58	0.82
d2-B1	C15H22N2O13P2	(H+)-1		500.0597	499.0524	499.0522	-0.48	-1	0.40	0.82
w6-B2	C53H70N18O35P6	(H+)-3		1704.2677	567.0819	567.0802	-3.14	-3	0.48	0.82
w6-B3	C53H70N18O35P6	(H+)-3		1704.2677	567.0819	567.0802	-3.14	-3	0.48	0.82
w6-B5	C53H70N18O35P6	(H+)-3		1704.2677	567.0819	567.0802	-3.14	-3	0.48	0.82
a3-B1	C24H31N5O15P2	(H+)-1		691.1292	690.1219	690.1225	0.81	-1	1.83	0.80
z3-B2	C24H32N5O15P2(+1)	(H+)-2		692.1370	690.1219	690.1222	0.38	-1	1.8	0.80
y6-B2	C53H75N20O32P5Pt(+2)	(H+)-4	Pt(NH3)2++	1853.3192	924.6445	924.6413	-3.46	-2	22.56	0.80
y6-B3	C53H75N20O32P5Pt(+2)	(H+)-4	Pt(NH3)2++	1853.3192	924.6445	924.6413	-3.46	-2	22.56	0.80
Y10-B1	C92H124N35O56P9Pt(+2)	(H+)-6	Pt(NH3)2++	3088.5218	770.6193	770.6156	-4.78	-4	5.82	0.78
w5 -B	C44H58N15O29P5	(H+)-3		1415.2213	470.7332	470.7329	-0.61	-3	0.5	0.78
x3-B1	C24H31N8O16P3	(H+)-4		780.1071	194.0195	194.0191	-2.16	-4	0.4	0.78
w7 -B	C62H82N21O41P7	(H+)-4		1993.3140	497.3212	497.3210	-0.46	-4	1.8	0.77
d9 -B	C82H107N28O54P9	(H+)-4		2626.4126	655.5959	655.5963	0.65	-4	4.6	0.77
w4	C39H51N15O24P4	(H+)-3		1237.2182	411.3988	411.3981	-1.65	-3	2.2	0.77
w2	C19H26N8O12P2	(H+)-2		620.1145	309.0500	309.0499	-0.46	-2	0.2	0.77
w2	C19H26N8O12P2	(H+)-2		620.1145	309.0500	309.0499	-0.46	-2	0.2	0.77
d2	C20H27N7O13P2	(H+)-1		635.1142	634.1069	634.1068	-0.18	-1	3.9	0.76
b4-B2	C35H49N14O21P3Pt(+2)	(H+)-4	Pt(NH3)2++	1289.2006	642.5878	642.5903	3.85	-2	2.08	0.76

Table S5. List of platinated CID fragments assigned by Aom^{2}s for the parent ion $[S1c+Pt(NH_3)_2-8H]^{6-}$ (*m/z* 689.6103) including fragment type, molecular formula (MF), theoretical and experimental *m/z*, error (in ppm), intensity (expressed as percentage of the base peak in the spectra) and similarity scores in percentage.

Type	MF	Ionization	Variable group	MF Mass	Th m/z	ppm	Charge	Intensity (%)	Similarity
a6	C59H79N24O34P5Pt(+2)	(H+)-5	(Pt(NH3)2++)	2017.3527	670.7717	0.51	-3	20.00	0.95
a12-B	C112H144N41O68P11Pt(+2)	(H+)-6	(Pt(NH3)++)	3686.5832	920.1346	0.00	-4	6.65	0.95
a12-B1	C112H144N41O68P11Pt(+2)	(H+)-6	(Pt(NH3)++)	3686.5832	920.1346	0.00	-4	6.65	0.95
b12-B3	C112H144N41O68P11Pt(+2)	(H+)-6	(Pt(NH3)++)	3686.5832	920.1346	0.00	-4	6.65	0.95
b12-B5	C112H144N41O68P11Pt(+2)	(H+)-6	(Pt(NH3)++)	3686.5832	920.1346	0.00	-4	6.65	0.95
a12-B8	C112H144N41O68P11Pt(+2)	(H+)-6	(Pt(NH3)++)	3686.5832	920.1346	0.00	-4	6.65	0.95
a12-B9	C112H144N41O68P11Pt(+2)	(H+)-6	(Pt(NH3)++)	3686.5832	920.1346	0.00	-4	6.65	0.95
a12-B11	C112H144N41O68P11Pt(+2)	(H+)-6	(Pt(NH3)++)	3686.5832	920.1346	0.00	-4	6.65	0.95
a12-B	C112H147N42O68P11Pt(+2)	(H+)-6	(Pt(NH3)2++)	3703.6098	924.3912	0.33	-4	15.80	0.95
a12-B1	C112H147N42O68P11Pt(+2)	(H+)-6	(Pt(NH3)2++)	3703.6098	924.3912	0.33	-4	15.80	0.95
b12-B3	C112H147N42O68P11Pt(+2)	(H+)-6	(Pt(NH3)2++)	3703.6098	924.3912	0.33	-4	15.80	0.95
b12-B5	C112H147N42O68P11Pt(+2)	(H+)-6	(Pt(NH3)2++)	3703.6098	924.3912	0.33	-4	15.80	0.95
a12-B8	C112H147N42O68P11Pt(+2)	(H+)-6	(Pt(NH3)2++)	3703.6098	924.3912	0.33	-4	15.80	0.95
a12-B9	C112H147N42O68P11Pt(+2)	(H+)-6	(Pt(NH3)2++)	3703.6098	924.3912	0.33	-4	15.80	0.95
a12-B11	C112H147N42O68P11Pt(+2)	(H+)-6	(Pt(NH3)2++)	3703.6098	924.3912	0.33	-4	15.80	0.95
a6	C59H76N23O34P5Pt(+2)	(H+)-4	(Pt(NH3)++)	2000.3261	998.1480	-0.05	-2	8.65	0.94
a10-B	C92H122N35O56P9Pt(+2)	(H+)-6	(Pt(NH3)2++)	3086.5061	770.1153	0.15	-4	5.63	0.93
a10-B1	C92H122N35O56P9Pt(+2)	(H+)-6	(Pt(NH3)2++)	3086.5061	770.1153	0.15	-4	5.63	0.93
b10-B3	C92H122N35O56P9Pt(+2)	(H+)-6	(Pt(NH3)2++)	3086.5061	770.1153	0.15	-4	5.63	0.93
b10-B5	C92H122N35O56P9Pt(+2)	(H+)-6	(Pt(NH3)2++)	3086.5061	770.1153	0.15	-4	5.63	0.93
a10-B8	C92H122N35O56P9Pt(+2)	(H+)-6	(Pt(NH3)2++)	3086.5061	770.1153	0.15	-4	5.63	0.93
a10-B9	C92H122N35O56P9Pt(+2)	(H+)-6	(Pt(NH3)2++)	3086.5061	770.1153	0.15	-4	5.63	0.93
y11:a12-B	C92H122N35O56P9Pt(+2)	(H+)-6	(Pt(NH3)2++)	3086.5061	770.1153	0.15	-4	5.63	0.93
a6	C59H79N24O34P5Pt(+2)	(H+)-4	(Pt(NH3)2++)	2017.3527	1006.6612	-0.18	-2	19.21	0.89
a10-B	C92H122N35O56P9Pt(+2)	(H+)-5	(Pt(NH3)2++)	3086.5061	1027.1562	-0.29	-3	7.64	0.89
a10-B1	C92H122N35O56P9Pt(+2)	(H+)-5	(Pt(NH3)2++)	3086.5061	1027.1562	-0.29	-3	7.64	0.89
b10-B3	C92H122N35O56P9Pt(+2)	(H+)-5	(Pt(NH3)2++)	3086.5061	1027.1562	-0.29	-3	7.64	0.89
b10-B5	C92H122N35O56P9Pt(+2)	(H+)-5	(Pt(NH3)2++)	3086.5061	1027.1562	-0.29	-3	7.64	0.89
a10-B8	C92H122N35O56P9Pt(+2)	(H+)-5	(Pt(NH3)2++)	3086.5061	1027.1562	-0.29	-3	7.64	0.89
a10-B9	C92H122N35O56P9Pt(+2)	(H+)-5	(Pt(NH3)2++)	3086.5061	1027.1562	-0.29	-3	7.64	0.89
y11:a12-B	C92H122N35O56P9Pt(+2)	(H+)-5	(Pt(NH3)2++)	3086.5061	1027.1562	-0.29	-3	7.64	0.89
	C92H117B3N33O55P9Pt(+2)	(H+)-6	(Pt(NH3)++)	3087.5204	770.3689	-3.83	-4	6.5	0.88
a10-B	C92H119N34O56P9Pt(+2)	(H+)-6	(Pt(NH3)++)	3069.4796	765.8587	0.47	-4	3.38	0.88
a10-B1	C92H119N34O56P9Pt(+2)	(H+)-6	(Pt(NH3)++)	3069.4796	765.8587	0.47	-4	3.38	0.88
b10-B3	C92H119N34O56P9Pt(+2)	(H+)-6	(Pt(NH3)++)	3069.4796	765.8587	0.47	-4	3.38	0.88
b10-B5	C92H119N34O56P9Pt(+2)	(H+)-6	(Pt(NH3)++)	3069.4796	765.8587	0.47	-4	3.38	0.88
a10-B8	C92H119N34O56P9Pt(+2)	(H+)-6	(Pt(NH3)++)	3069.4796	765.8587	0.47	-4	3.38	0.88
a10-B9	C92H119N34O56P9Pt(+2)	(H+)-6	(Pt(NH3)++)	3069.4796	765.8587	0.47	-4	3.38	0.88
y11:a12-B	C92H119N34O56P9Pt(+2)	(H+)-6	(Pt(NH3)++)	3069.4796	765.8587	0.47	-4	3.38	0.88
a6-B	C54H74N19O33P5Pt(+2)	(H+)-4	(Pt(NH3)2++)	1866.3032	931.1365	1.12	-2	3.01	0.85
a6-B3	C54H74N19O33P5Pt(+2)	(H+)-4	(Pt(NH3)2++)	1866.3032	931.1365	1.12	-2	3.01	0.85
a6-B5	C54H74N19O33P5Pt(+2)	(H+)-4	(Pt(NH3)2++)	1866.3032	931.1365	1.12	-2	3.01	0.85

Type	MF	Ionization	Variable group	MF Mass	Th m/z	ppm	Charge	Intensity (%)	Similarity
a8-B	C73H95N26O45P7Pt(+2)	(H+)-5	(Pt(NH3)++)	2467.3756	820.7794	-0.86	-3	1.01	0.85
a8-B2	C73H95N26O45P7Pt(+2)	(H+)-5	(Pt(NH3)++)	2467.3756	820.7794	-0.86	-3	1.01	0.85
a8-B6	C73H95N26O45P7Pt(+2)	(H+)-5	(Pt(NH3)++)	2467.3756	820.7794	-0.86	-3	1.01	0.85
a8-B7	C73H95N26O45P7Pt(+2)	(H+)-5	(Pt(NH3)++)	2467.3756	820.7794	-0.86	-3	1.01	0.85
a11	C107H140N42O63P10Pt(+2)	(H+)-6	(Pt(NH3)2++)	3525.6066	879.8905	1.12	-4	1.32	0.84
	C44H58B3N19O23P4Pt(+2)	(H+)-4	(Pt(NH3)++)	3087.5204	1027.4943	-4.80	-3	4.92	0.83
	C92H117B3N33O55P9Pt(+2)	(H+)-6	(Pt++)	3070.4938	766.1123	-4.20	-4	3.31	0.82
a9-B	C82H107N29O51P8Pt(+2)	(H+)-5	(Pt(NH3)++)	2756.4220	917.1282	0.46	-3	1.58	0.82
a9-B1	C82H107N29O51P8Pt(+2)	(H+)-5	(Pt(NH3)++)	2756.4220	917.1282	0.46	-3	1.58	0.82
b9-B3	C82H107N29O51P8Pt(+2)	(H+)-5	(Pt(NH3)++)	2756.4220	917.1282	0.46	-3	1.58	0.82
b9-B5	C82H107N29O51P8Pt(+2)	(H+)-5	(Pt(NH3)++)	2756.4220	917.1282	0.46	-3	1.58	0.82
a9-B8	C82H107N29O51P8Pt(+2)	(H+)-5	(Pt(NH3)++)	2756.4220	917.1282	0.46	-3	1.58	0.82
y6-B2	C53H75N20O32P5Pt(+2)	(H+)-4	(Pt(NH3)2++)	1853.3192	924.6445	-3.46	-2	22.56	0.80
y6-B3	C53H75N20O32P5Pt(+2)	(H+)-4	(Pt(NH3)2++)	1853.3192	924.6445	-3.46	-2	22.56	0.80
y10-B1	C92H124N35O56P9Pt(+2)	(H+)-6	(Pt(NH3)2++)	3088.5218	770.6193	-4.78	-4	5.82	0.78
b4-B2	C35H49N14O21P3Pt(+2)	(H+)-4	(Pt(NH3)2++)	1289.2057	642.5878	3.85	-2	2.08	0.76

Table S6. List of CID fragments assigned by Aom²s for the parent ion $[S1-5H]^{5-}$ (*m/z* 796.533) including fragment type, molecular formula (MF), experimental *m/z*, error (in ppm), intensity (expressed as percentage of the most abundant peak in the spectra) and similarity scores in percentage

Type	MF	Ionization	MF Mass	Th m/z	Exp m/z	ppm	Charge	Intensity (%)	Similarity
	C123H156N44O77P12	(H+)-5	3852.6495	769.5226	769.5227	0.11	-5	12.80	0.97
w4	C39H51N15O25P4	(H+)-2	1253.2131	625.5993	625.5991	-0.32	-2	65.20	0.97
w5:b12	C39H51N15O25P4	(H+)-2	1253.2131	625.5993	625.5991	-0.32	-2	65.20	0.97
a3-B	C25H31N7O15P2	(H+)-1	731.1353	730.1281	730.1282	0.15	-1	100.07	0.96
a3-B2	C25H31N7O15P2	(H+)-1	731.1353	730.1281	730.1282	0.15	-1	100.07	0.96
y9:a7-B	C25H31N7O15P2	(H+)-1	731.1353	730.1281	730.1282	0.15	-1	100.07	0.96
w5	C49H63N20O30P5	(H+)-2	1566.2707	782.1281	782.1281	0.00	-2	14.13	0.95
a7-B	C65H81N21O40P6	(H+)-2	1981.3375	989.6615	989.6612	-0.28	-2	15.94	0.94
a7-B5	C65H81N21O40P6	(H+)-2	1981.3375	989.6615	989.6612	-0.28	-2	15.94	0.94
a7-B6	C65H81N21O40P6	(H+)-2	1981.3375	989.6615	989.6612	-0.28	-2	15.94	0.94
y12:a8-B	C65H81N21O40P6	(H+)-2	1981.3375	989.6615	989.6612	-0.28	-2	15.94	0.94
a6-B	C55H69N16O34P5	(H+)-2	1652.2850	825.1352	825.1351	-0.12	-2	22.82	0.94
a6-B5	C55H69N16O34P5	(H+)-2	1652.2850	825.1352	825.1351	-0.12	-2	22.82	0.94
y12:a7-B	C55H69N16O34P5	(H+)-2	1652.2850	825.1352	825.1351	-0.12	-2	22.82	0.94
w6-B2	C53H70N18O36P6	(H+)-2	1720.2626	859.1240	859.1257	1.95	-2	0.93	0.94
w6-B6	C53H70N18O36P6	(H+)-2	1720.2626	859.1240	859.1257	1.95	-2	0.93	0.94
a9-B	C84H105N29O52P8	(H+)-3	2599.4364	865.4715	865.4713	-0.27	-3	17.91	0.94
a9-B2	C84H105N29O52P8	(H+)-3	2599.4364	865.4715	865.4713	-0.27	-3	17.91	0.94
b9-B5	C84H105N29O52P8	(H+)-3	2599.4364	865.4715	865.4713	-0.27	-3	17.91	0.94
b9-B6	C84H105N29O52P8	(H+)-3	2599.4364	865.4715	865.4713	-0.27	-3	17.91	0.94
a9-B8	C84H105N29O52P8	(H+)-3	2599.4364	865.4715	865.4713	-0.27	-3	17.91	0.94
w4-B4	C34H46N10O25P4	(H+)-2	1118.1586	558.0720	558.0723	0.43	-2	3.18	0.93
y6	C58H74N23O33P5	(H+)-2	1775.3507	886.6681	886.6696	1.70	-2	4.03	0.92
d10	C98H125N37O62P10	(H+)-4	3121.5142	779.3713	779.3709	-0.42	-4	11.90	0.92
w10	C98H125N37O62P10	(H+)-4	3121.5142	779.3713	779.3709	-0.42	-4	11.90	0.92
w12:b11	C98H125N37O62P10	(H+)-4	3121.5142	779.3713	779.3709	-0.42	-4	11.90	0.92
w11:b12	C98H125N37O62P10	(H+)-4	3121.5142	779.3713	779.3709	-0.42	-4	11.90	0.92
a8-B	C75H93N26O46P7	(H+)-2	2310.3901	1154.1878	1154.1889	0.97	-2	1.46	0.91
a8-B7	C75H93N26O46P7	(H+)-2	2310.3901	1154.1878	1154.1889	0.97	-2	1.46	0.91
w6:a9-B	C14H19N3O11P2	(H+)-1	467.0495	466.0422	466.0421	-0.22	-1	10.18	0.91
w4:a11-B	C14H19N3O11P2	(H+)-1	467.0495	466.0422	466.0421	-0.22	-1	10.18	0.91
w7	C68H87N28O42P7	(H+)-3	2184.3696	727.1159	727.1164	0.65	-3	5.09	0.91
c1	C10H12N5O6P	(H+)-1	329.0525	328.0452	328.0450	-0.85	-1	2.41	0.91
	C124H156N46O76P12	(H+)-5	3876.6608	774.3249	774.3258	1.18	-5	2.37	0.91
	C124H156N46O76P12	(H+)-5	3876.6608	774.3249	774.3258	1.18	-5	2.37	0.91
d10-B2	C93H120N32O62P10	(H+)-4	2986.4597	745.6076	745.6084	1.01	-4	4.40	0.91
d10-B8	C93H120N32O62P10	(H+)-4	2986.4597	745.6076	745.6084	1.01	-4	4.40	0.91
w10-B6	C93H120N32O62P10	(H+)-4	2986.4597	745.6076	745.6084	1.01	-4	4.40	0.91
w10-B10	C93H120N32O62P10	(H+)-4	2986.4597	745.6076	745.6084	1.01	-4	4.40	0.91

Type	MF	Ionization	MF Mass	Th m/z	Exp m/z	ppm	Charge	Intensity (%)	Similarity
w11:a9-B	C64H81N22O42P7	(H+)-2	2046.3042	1022.1448	1022.1458	0.96	-2	1.54	0.90
w10:a10-B	C64H81N22O42P7	(H+)-2	2046.3042	1022.1448	1022.1458	0.96	-2	1.54	0.90
a7-B	C65H81N21O40P6	(H+)-3	1981.3375	659.4386	659.4393	1.14	-3	4.60	0.90
a7-B5	C65H81N21O40P6	(H+)-3	1981.3375	659.4386	659.4393	1.14	-3	4.60	0.90
a7-B6	C65H81N21O40P6	(H+)-3	1981.3375	659.4386	659.4393	1.14	-3	4.60	0.90
y12:a8-B	C65H81N21O40P6	(H+)-3	1981.3375	659.4386	659.4393	1.14	-3	4.60	0.90
y4	C39H50N15O22P3	(H+)-1	1173.2468	1172.2395	1172.2397	0.21	-1	11.59	0.89
w10:a9-B	C54H69N17O37P6	(H+)-2	1733.2466	865.6160	865.6161	0.04	-2	22.87	0.89
c2-B1	C15H19N5O11P2	(H+)-1	507.0556	506.0484	506.0482	-0.34	-1	10.92	0.89
w8:a7-B	C15H19N5O11P2	(H+)-1	507.0556	506.0484	506.0482	-0.34	-1	10.92	0.89
w7:a8-B	C15H19N5O11P2	(H+)-1	507.0556	506.0484	506.0482	-0.34	-1	10.92	0.89
w3:a12-B	C15H19N5O11P2	(H+)-1	507.0556	506.0484	506.0482	-0.34	-1	10.92	0.89
w9:a8-B	C35H44N12O24P4	(H+)-2	1140.1542	569.0698	569.0704	0.97	-2	0.32	0.89
w10:a8-B	C45H57N14O31P5	(H+)-2	1444.2002	721.0928	721.0917	-1.62	-2	11.09	0.88
	C123H156N44O76P12	(H+)-5	3836.6546	766.3236	766.3231	-0.76	-5	1.96	0.88
	C123H156N44O76P12	(H+)-5	3836.6546	766.3236	766.3231	-0.76	-5	1.96	0.88
	C123H156N44O76P12	(H+)-5	3836.6546	766.3236	766.3231	-0.76	-5	1.96	0.88
w6	C58H75N23O36P6	(H+)-2	1855.3171	926.6513	926.6522	0.99	-2	7.37	0.88
x1	C10H12N5O5P	(H+)-1	313.0576	312.0503	312.0502	-0.41	-1	1.23	0.87
a2-B	C15H18N5O8P	(H+)-1	427.0893	426.0820	426.0819	-0.25	-1	7.19	0.87
a2-B1	C15H18N5O8P	(H+)-1	427.0893	426.0820	426.0819	-0.25	-1	7.19	0.87
y8:a7-B	C15H18N5O8P	(H+)-1	427.0893	426.0820	426.0819	-0.25	-1	7.19	0.87
y7:a8-B	C15H18N5O8P	(H+)-1	427.0893	426.0820	426.0819	-0.25	-1	7.19	0.87
y3:a12-B	C15H18N5O8P	(H+)-1	427.0893	426.0820	426.0819	-0.25	-1	7.19	0.87
w1	C10H14N5O6P	(H+)-1	331.0682	330.0609	330.0602	-1.98	-1	0.47	0.87
w10:a6-B	C25H33N4O19P3	(H+)-1	786.0952	785.0879	785.0891	1.45	-1	7.47	0.86
c3	C30H37N12O18P3	(H+)-1	946.1562	945.1489	945.1500	1.15	-1	0.47	0.86
x3	C30H37N12O18P3	(H+)-1	946.1562	945.1489	945.1500	1.15	-1	0.47	0.86
x2-B2	C15H20N2O12P2	(H+)-1	482.0491	481.0419	481.0420	0.37	-1	7.72	0.86
w12:a3-B	C15H20N2O12P2	(H+)-1	482.0491	481.0419	481.0420	0.37	-1	7.72	0.86
w10:a5-B	C15H20N2O12P2	(H+)-1	482.0491	481.0419	481.0420	0.37	-1	7.72	0.86
w9:a6-B	C15H20N2O12P2	(H+)-1	482.0491	481.0419	481.0420	0.37	-1	7.72	0.86
d3	C30H39N12O19P3	(H+)-2	964.1667	481.0761	481.0762	0.23	-2	4.99	0.85
w3	C30H39N12O19P3	(H+)-2	964.1667	481.0761	481.0762	0.23	-2	4.99	0.85
b3	C30H38N12O16P2	(H+)-1	884.2004	883.1931	883.1948	1.95	-1	7.53	0.85
y3	C30H38N12O16P2	(H+)-1	884.2004	883.1931	883.1948	1.95	-1	7.53	0.85
w2	C20H27N7O13P2	(H+)-1	635.1142	634.1069	634.1075	0.91	-1	7.45	0.85
w12:b3	C20H27N7O13P2	(H+)-1	635.1142	634.1069	634.1075	0.91	-1	7.45	0.85
w11:b4	C20H27N7O13P2	(H+)-1	635.1142	634.1069	634.1075	0.91	-1	7.45	0.85
d3	C30H39N12O19P3	(H+)-1	964.1667	963.1595	963.1603	0.85	-1	8.67	0.85
w3	C30H39N12O19P3	(H+)-1	964.1667	963.1595	963.1603	0.85	-1	8.67	0.85
c3-B2	C25H32N7O18P3	(H+)-1	811.1017	810.0944	810.0947	0.38	-1	9.14	0.84
x3-B3	C25H32N7O18P3	(H+)-1	811.1017	810.0944	810.0947	0.38	-1	9.14	0.84
w9:a7-B	C25H32N7O18P3	(H+)-1	811.1017	810.0944	810.0947	0.38	-1	9.14	0.84
a2	C20H24N7O10P	(H+)-1	553.1322	552.1250	552.1255	1.01	-1	4.98	0.84
w6	C58H75N23O36P6	(H+)-3	1855.3171	617.4317	617.4317	-0.06	-3	10.75	0.84
c8-B5	C74H94N24O49P8	(H+)-3	2350.3502	782.4428	782.4438	1.27	-3	6.24	0.83
c8-B6	C74H94N24O49P8	(H+)-3	2350.3502	782.4428	782.4438	1.27	-3	6.24	0.83

Type	MF	Ionization	MF Mass	Th m/z	Exp m/z	ppm	Charge	Intensity (%)	Similarity
w12:a9-B	C74H94N24O49P8	(H+)-3	2350.3502	782.4428	782.4438	1.27	-3	6.24	0.83
y12:a3-B	C15H19N2O9P	(H+)-1	402.0828	401.0755	401.0751	-1.17	-1	0.38	0.82
y10:a5-B	C15H19N2O9P	(H+)-1	402.0828	401.0755	401.0751	-1.17	-1	0.38	0.82
y9:a6-B	C15H19N2O9P	(H+)-1	402.0828	401.0755	401.0751	-1.17	-1	0.38	0.82
w8:b10	C48H62N21O30P5	(H+)-2	1567.2660	782.6257	782.6299	5.32	-2	8.60	0.82
w7:b11	C48H62N21O30P5	(H+)-2	1567.2660	782.6257	782.6299	5.32	-2	8.60	0.82
x9-B5	C83H105N30O54P9	(H+)-3	2664.4031	887.1271	887.1313	4.72	-3	0.40	0.82
x9-B9	C83H105N30O54P9	(H+)-3	2664.4031	887.1271	887.1313	4.72	-3	0.40	0.82
w10:a12-B	C83H105N30O54P9	(H+)-3	2664.4031	887.1271	887.1313	4.72	-3	0.40	0.82
w5-B1	C44H58N15O30P5	(H+)-2	1431.2162	714.6008	714.6020	1.59	-2	1.15	0.82
w5-B5	C44H58N15O30P5	(H+)-2	1431.2162	714.6008	714.6020	1.59	-2	1.15	0.82
w2-B2	C15H22N2O13P2	(H+)-1	500.0597	499.0524	499.0527	0.60	-1	0.27	0.82
x2-B1	C15H19N5O10P2	(H+)-1	491.0607	490.0534	490.0535	0.19	-1	1.37	0.81
w11:a4-B	C15H19N5O10P2	(H+)-1	491.0607	490.0534	490.0535	0.19	-1	1.37	0.81
w5:a10-B	C15H19N5O10P2	(H+)-1	491.0607	490.0534	490.0535	0.19	-1	1.37	0.81
w4	C39H51N15O25P4	(H+)-1	1253.2131	1252.2058	1252.2069	0.89	-1	7.74	0.81
w5:b12	C39H51N15O25P4	(H+)-1	1253.2131	1252.2058	1252.2069	0.89	-1	7.74	0.81
w7-B3	C63H82N23O42P7	(H+)-3	2049.3151	682.0978	682.0978	0.03	-3	0.61	0.81
w7-B7	C63H82N23O42P7	(H+)-3	2049.3151	682.0978	682.0978	0.03	-3	0.61	0.81
w10:a9-B	C54H69N17O37P6	(H+)-3	1733.2466	576.7416	576.7425	1.51	-3	1.51	0.81
d12-B5	C113H145N39O74P12	(H+)-5	3603.5633	719.7054	719.7071	2.32	-5	1.09	0.80
d12-B6	C113H145N39O74P12	(H+)-5	3603.5633	719.7054	719.7071	2.32	-5	1.09	0.80
d12-B10	C113H145N39O74P12	(H+)-5	3603.5633	719.7054	719.7071	2.32	-5	1.09	0.80
w12-B2	C113H145N39O74P12	(H+)-5	3603.5633	719.7054	719.7071	2.32	-5	1.09	0.80
w12-B8	C113H145N39O74P12	(H+)-5	3603.5633	719.7054	719.7071	2.32	-5	1.09	0.80
w12-B12	C113H145N39O74P12	(H+)-5	3603.5633	719.7054	719.7071	2.32	-5	1.09	0.80
a3	C30H36N12O15P2	(H+)-1	866.1898	865.1826	865.1842	1.88	-1	2.83	0.80
z3	C30H36N12O15P2	(H+)-1	866.1898	865.1826	865.1842	1.88	-1	2.83	0.80
y7	C68H86N28O39P6	(H+)-2	2104.4033	1051.1944	1051.1971	2.64	-2	4.08	0.80
a6-B	C55H69N16O34P5	(H+)-3	1652.2850	549.7544	549.7544	-0.06	-3	0.53	0.80
a6-B5	C55H69N16O34P5	(H+)-3	1652.2850	549.7544	549.7544	-0.06	-3	0.53	0.80
y12:a7-B	C55H69N16O34P5	(H+)-3	1652.2850	549.7544	549.7544	-0.06	-3	0.53	0.80
w9	C88H112N35O55P9	(H+)-4	2817.4682	703.3598	703.3606	1.13	-4	0.82	0.79
w11:b11	C88H112N35O55P9	(H+)-4	2817.4682	703.3598	703.3606	1.13	-4	0.82	0.79
w8:a7	C20H24N10O12P2	(H+)-2	658.1050	328.0452	328.0450	-0.85	-2	2.41	0.79
c9-B5	C84H106N29O54P9	(H+)-3	2663.4078	886.7953	886.7954	0.10	-3	0.13	0.79
c9-B6	C84H106N29O54P9	(H+)-3	2663.4078	886.7953	886.7954	0.10	-3	0.13	0.79
w12:a10-B	C84H106N29O54P9	(H+)-3	2663.4078	886.7953	886.7954	0.10	-3	0.13	0.79
a11-B	C103H129N37O63P10	(H+)-4	3201.5404	799.3778	799.3791	1.60	-4	0.46	0.79
a11-B5	C103H129N37O63P10	(H+)-4	3201.5404	799.3778	799.3791	1.60	-4	0.46	0.79
a11-B6	C103H129N37O63P10	(H+)-4	3201.5404	799.3778	799.3791	1.60	-4	0.46	0.79
a11-B10	C103H129N37O63P10	(H+)-4	3201.5404	799.3778	799.3791	1.60	-4	0.46	0.79
y12:a12-B	C103H129N37O63P10	(H+)-4	3201.5404	799.3778	799.3791	1.60	-4	0.46	0.79
c4-B2	C35H45N9O25P4	(H+)-1	1115.1477	1114.1404	1114.1427	2.01	-1	5.07	0.78
w10:a7-B	C35H45N9O25P4	(H+)-1	1115.1477	1114.1404	1114.1427	2.01	-1	5.07	0.78
a10-B	C94H117N34O57P9	(H+)-4	2912.4940	727.1162	727.1164	0.22	-4	5.09	0.77

Type	MF	Ionization	MF Mass	Th m/z	Exp m/z	ppm	Charge	Intensity (%)	Similarity
a10-B7	C94H117N34O57P9	(H+)-4	2912.4940	727.1162	727.1164	0.22	-4	5.09	0.77
a10-B9	C94H117N34O57P9	(H+)-4	2912.4940	727.1162	727.1164	0.22	-4	5.09	0.77
y10:a9-B	C54H68N17O34P5	(H+)-2	1653.2803	825.6329	825.6366	4.52	-2	17.78	0.77
y5	C49H62N20O27P4	(H+)-2	1486.3044	742.1449	742.1465	2.21	-2	1.00	0.77
z2	C20H24N7O9P	(H+)-1	537.1373	536.1300	536.1301	0.05	-1	0.12	0.77
w6:b9	C19H26N8O12P2	(H+)-1	620.1145	619.1073	619.1074	0.28	-1	0.26	0.77
w5:b10	C19H26N8O12P2	(H+)-1	620.1145	619.1073	619.1074	0.28	-1	0.26	0.77
w10:a5	C20H26N4O14P2	(H+)-2	608.0921	303.0388	303.0383	-1.47	-2	5.12	0.77
w10:a5	C20H26N4O14P2	(H+)-1	608.0921	607.0848	607.0861	2.18	-1	0.50	0.77
b10	C98H124N37O59P9	(H+)-3	3041.5479	1012.8420	1012.8432	1.18	-3	0.73	0.76
y10	C98H124N37O59P9	(H+)-3	3041.5479	1012.8420	1012.8432	1.18	-3	0.73	0.76
w9-B2	C83H107N30O54P9	(H+)-3	2666.4187	887.7990	887.7987	-0.30	-3	0.13	0.76
w9-B3	C83H107N30O54P9	(H+)-3	2666.4187	887.7990	887.7987	-0.30	-3	0.13	0.76
w9-B7	C83H107N30O54P9	(H+)-3	2666.4187	887.7990	887.7987	-0.30	-3	0.13	0.76
d2	C20H27N7O14P2	(H+)-3	651.1091	216.0291	216.0293	1.03	-3	0.10	0.76
w9:b6	C20H27N7O14P2	(H+)-3	651.1091	216.0291	216.0293	1.03	-3	0.10	0.76
w3:b12	C20H27N7O14P2	(H+)-3	651.1091	216.0291	216.0293	1.03	-3	0.10	0.76
d2	C20H27N7O14P2	(H+)-1	651.1091	650.1018	650.1029	1.67	-1	3.38	0.76
w9:b6	C20H27N7O14P2	(H+)-1	651.1091	650.1018	650.1029	1.67	-1	3.38	0.76
w3:b12	C20H27N7O14P2	(H+)-1	651.1091	650.1018	650.1029	1.67	-1	3.38	0.76
w5	C49H63N20O30P5	(H+)-3	1566.2707	521.0830	521.0829	-0.07	-3	2.75	0.76
w7-B1	C63H82N23O41P7	(H+)-3	2033.3202	676.7661	676.7677	2.38	-3	0.14	0.75
w7-B5	C63H82N23O41P7	(H+)-3	2033.3202	676.7661	676.7677	2.38	-3	0.14	0.75
b10-B1	C93H116N35O57P9	(H+)-4	2913.4893	727.3650	727.3680	4.07	-4	2.71	0.75
b10-B3	C93H116N35O57P9	(H+)-4	2913.4893	727.3650	727.3680	4.07	-4	2.71	0.75
b10-B4	C93H116N35O57P9	(H+)-4	2913.4893	727.3650	727.3680	4.07	-4	2.71	0.75
w12:a6-B	C45H58N11O31P5	(H+)-2	1403.1988	700.5921	700.5929	1.05	-2	1.72	0.73
w10:a7	C40H50N14O26P4	(H+)-1	1266.1971	1265.1898	1265.1860	-3.00	-1	6.04	0.73
x4-B3	C34H43N13O22P4	(H+)-1	1109.1596	1108.1523	1108.1546	2.08	-1	1.18	0.73
w7:a10-B	C34H43N13O22P4	(H+)-1	1109.1596	1108.1523	1108.1546	2.08	-1	1.18	0.73
w5:a12-B	C34H43N13O22P4	(H+)-1	1109.1596	1108.1523	1108.1546	2.08	-1	1.18	0.73
b8-B1	C74H92N27O46P7	(H+)-2	2311.3853	1154.6854	1154.6912	5.09	-2	2.00	0.73
b8-B3	C74H92N27O46P7	(H+)-2	2311.3853	1154.6854	1154.6912	5.09	-2	2.00	0.73
b8-B4	C74H92N27O46P7	(H+)-2	2311.3853	1154.6854	1154.6912	5.09	-2	2.00	0.73
y6:a10-B	C24H30N8O13P2	(H+)-1	700.1408	699.1335	699.1356	2.98	-1	0.14	0.72
y5:a11-B	C24H30N8O13P2	(H+)-1	700.1408	699.1335	699.1356	2.98	-1	0.14	0.72
c2	C20H25N7O13P2	(H+)-1	633.0986	632.0913	632.0895	-2.87	-1	10.30	0.72
w9:a6	C20H25N7O13P2	(H+)-1	633.0986	632.0913	632.0895	-2.87	-1	10.30	0.72
w3:a12	C20H25N7O13P2	(H+)-1	633.0986	632.0913	632.0895	-2.87	-1	10.30	0.72
w10:a6-B	C25H33N4O19P3	(H+)-2	786.0952	392.0403	392.0397	-1.61	-2	0.20	0.72
a10-B	C94H117N34O57P9	(H+)-3	2912.4940	969.8241	969.8267	2.72	-3	0.52	0.72
a10-B7	C94H117N34O57P9	(H+)-3	2912.4940	969.8241	969.8267	2.72	-3	0.52	0.72
a10-B9	C94H117N34O57P9	(H+)-3	2912.4940	969.8241	969.8267	2.72	-3	0.52	0.72
a6	C60H74N21O35P5	(H+)-2	1803.3344	900.6599	900.6620	2.34	-2	0.44	0.72
w12	C118H150N44O74P12	(H+)-5	3738.6178	746.7163	746.7178	1.99	-5	1.64	0.71
w8:b8	C29H38N13O19P3	(H+)-1	965.1620	964.1547	964.1642	9.88	-1	2.13	0.71
d9-B1	C84H107N32O54P9	(H+)-4	2706.4249	675.5989	675.6014	3.65	-4	0.12	0.71
d9-B3	C84H107N32O54P9	(H+)-4	2706.4249	675.5989	675.6014	3.65	-4	0.12	0.71
d9-B4	C84H107N32O54P9	(H+)-4	2706.4249	675.5989	675.6014	3.65	-4	0.12	0.71
w9-B4	C84H107N32O54P9	(H+)-4	2706.4249	675.5989	675.6014	3.65	-4	0.12	0.71
w9-B6	C84H107N32O54P9	(H+)-4	2706.4249	675.5989	675.6014	3.65	-4	0.12	0.71

Table S7. List of CID fragments assigned by Aom²s for the parent ion $[S1c\text{-}5H]^{5-}$ (*m/z* 782.3331) including fragment type, molecular formula (MF), experimental *m/z*, error (in ppm), intensity (expressed as percentage of the most abundant peak in the spectra) similarity scores in percentage

Type	MF	Ionization	MF Mass	Th m/z	Exp m/z	ppm	Charge	Intensity (%)	Similarity
w1	C9H14N3O7P	(H+)-1	307.0569	306.0497	306.0498	0.61	-1	81.00	0.98
w6	C58H75N23O35P6	(H+)-3	1839.3222	612.1001	612.1001	-0.06	-3	21.17	0.97
w7:b12	C58H75N23O35P6	(H+)-3	1839.3222	612.1001	612.1001	-0.06	-3	21.17	0.97
w3	C29H39N10O19P3	(H+)-2	924.1606	461.0730	461.0729	-0.14	-2	34.87	0.97
w9:a6-B	C15H20N2O12P2	(H+)-1	482.0491	481.0419	481.0420	0.22	-1	41.92	0.97
w3:a12-B	C15H20N2O12P2	(H+)-1	482.0491	481.0419	481.0420	0.22	-1	41.92	0.97
w5:a12-B	C35H44N12O22P4	(H+)-1	1108.1644	1107.1571	1107.1569	-0.21	-1	12.12	0.97
w12:a8-B	C63H80N23O41P7	(H+)-2	2031.3045	1014.6450	1014.6451	0.09	-2	8.71	0.96
w10:a10-B	C63H80N23O41P7	(H+)-2	2031.3045	1014.6450	1014.6451	0.09	-2	8.71	0.96
w7	C67H87N26O41P7	(H+)-3	2128.3685	708.4489	708.4488	-0.08	-3	13.00	0.96
w10:a7-B	C35H44N12O24P4	(H+)-1	1140.1542	1139.1469	1139.1462	-0.59	-1	8.40	0.96
w4:a12-B	C25H32N7O17P3	(H+)-1	795.1068	794.0995	794.0997	0.27	-1	31.94	0.96
w9	C87H112N33O54P9	(H+)-4	2761.4671	689.3595	689.3595	0.02	-4	6.28	0.96
w6	C58H75N23O35P6	(H+)-2	1839.3222	918.6538	918.6537	-0.09	-2	25.83	0.96
w7:b12	C58H75N23O35P6	(H+)-2	1839.3222	918.6538	918.6537	-0.09	-2	25.83	0.96
w4	C39H51N15O24P4	(H+)-2	1237.2182	617.6018	617.6018	-0.08	-2	101.03	0.96
w6:b11	C39H51N15O24P4	(H+)-2	1237.2182	617.6018	617.6018	-0.08	-2	101.03	0.96
w12:a6-B	C44H56N15O29P5	(H+)-1	1413.2056	1412.1984	1412.1993	0.66	-1	2.94	0.96
w7:a10-B	C33H43N11O22P4	(H+)-1	1069.1535	1068.1462	1068.1461	-0.08	-1	20.33	0.96
w11:a9-B	C62H80N21O42P7	(H+)-2	2007.2933	1002.6394	1002.6392	-0.14	-2	7.63	0.95
w3	C29H39N10O19P3	(H+)-1	924.1606	923.1533	923.1533	0.03	-1	73.67	0.95
y9:a6-B	C15H19N2O9P	(H+)-1	402.0828	401.0755	401.0755	0.01	-1	13.76	0.95
y3:a12-B	C15H19N2O9P	(H+)-1	402.0828	401.0755	401.0755	0.01	-1	13.76	0.95
w6:a12-B	C44H56N15O28P5	(H+)-2	1397.2107	697.5981	697.5981	0.05	-2	27.22	0.95
w9:a10-B	C53H68N18O35P6	(H+)-2	1702.2520	850.1187	850.1186	-0.18	-2	26.10	0.95
y11:a12-B	C92H116N33O56P9	(H+)-3	2857.4882	951.4888	951.4886	-0.26	-3	34.17	0.95
y11:a12-B	C92H116N33O56P9	(H+)-4	2857.4882	713.3648	713.3648	0.00	-4	33.97	0.95
w12:a4-B	C24H31N8O16P3	(H+)-1	780.1071	779.0998	779.0999	0.16	-1	43.49	0.95
w6:a10-B	C24H31N8O16P3	(H+)-1	780.1071	779.0998	779.0999	0.16	-1	43.49	0.95
w9:a9-B	C43H56N13O30P5	(H+)-2	1389.1944	693.5899	693.5899	-0.04	-2	27.04	0.95
y12:a9-B	C72H91N26O44P7	(H+)-2	2240.3846	1119.1850	1119.1859	0.82	-2	3.57	0.95
y11:a10-B	C72H91N26O44P7	(H+)-2	2240.3846	1119.1850	1119.1859	0.82	-2	3.57	0.95
y10:a6-B	C25H31N7O15P2	(H+)-1	731.1353	730.1281	730.1281	0.11	-1	9.77	0.95
y9:a7-B	C25H31N7O15P2	(H+)-1	731.1353	730.1281	730.1281	0.11	-1	9.77	0.95
w11:a7-B	C44H56N15O30P5	(H+)-1	1429.2006	1428.1933	1428.1938	0.39	-1	5.72	0.94
w10:a8-B	C44H56N15O30P5	(H+)-1	1429.2006	1428.1933	1428.1938	0.39	-1	5.72	0.94
w4	C39H51N15O24P4	(H+)-1	1237.2182	1236.2109	1236.2108	-0.05	-1	18.76	0.94
w6:b11	C39H51N15O24P4	(H+)-1	1237.2182	1236.2109	1236.2108	-0.05	-1	18.76	0.94
w12:a3-B	C15H19N5O10P2	(H+)-1	491.0607	490.0534	490.0534	-0.06	-1	19.15	0.94

Type	MF	Ionization	MF Mass	Th m/z	Exp m/z	ppm	Charge	Intensity (%)	Similarity
w5:a10-B	C15H19N5O10P2	(H+)-1	491.0607	490.0534	490.0534	-0.06	-1	19.15	0.94
w4:a11-B	C15H19N5O10P2	(H+)-1	491.0607	490.0534	490.0534	-0.06	-1	19.15	0.94
w6:a12-B	C44H56N15O28P5	(H+)-1	1397.2107	1396.2035	1396.2030	-0.35	-1	6.21	0.94
y4:a12-B	C25H31N7O14P2	(H+)-1	715.1404	714.1331	714.1333	0.17	-1	45.45	0.94
y12:a4-B	C24H30N8O13P2	(H+)-1	700.1408	699.1335	699.1336	0.19	-1	3.07	0.94
y6:a10-B	C24H30N8O13P2	(H+)-1	700.1408	699.1335	699.1336	0.19	-1	3.07	0.94
w11:a4-B	C14H19N3O11P2	(H+)-1	467.0495	466.0422	466.0422	-0.06	-1	23.19	0.94
w7:a8-B	C14H19N3O11P2	(H+)-1	467.0495	466.0422	466.0422	-0.06	-1	23.19	0.94
w6:a9-B	C14H19N3O11P2	(H+)-1	467.0495	466.0422	466.0422	-0.06	-1	23.19	0.94
a7	C68H85N25O40P6	(H+)-2	2077.3811	1037.6833	1037.6834	0.09	-2	8.99	0.94
w7	C67H87N26O41P7	(H+)-2	2128.3685	1063.1770	1063.1775	0.52	-2	6.19	0.93
w11:a8-B	C53H68N18O36P6	(H+)-2	1718.2469	858.1162	858.1162	0.02	-2	19.93	0.93
w10:a9-B	C53H68N18O36P6	(H+)-2	1718.2469	858.1162	858.1162	0.02	-2	19.93	0.93
w12:a9-B	C72H92N26O47P8	(H+)-2	2320.3509	1159.1682	1159.1686	0.40	-2	10.07	0.93
w11:a10-B	C72H92N26O47P8	(H+)-2	2320.3509	1159.1682	1159.1686	0.40	-2	10.07	0.93
w12:a10-B	C82H104N31O52P9	(H+)-3	2633.4085	876.7956	876.7949	-0.78	-3	6.14	0.93
w11:a11-B	C82H104N31O52P9	(H+)-3	2633.4085	876.7956	876.7949	-0.78	-3	6.14	0.93
w5:a12-B	C35H44N12O22P4	(H+)-2	1108.1644	553.0749	553.0750	0.19	-2	10.24	0.92
w10:a6-B	C25H32N7O18P3	(H+)-1	811.1017	810.0944	810.0943	-0.06	-1	17.91	0.92
w9:a7-B	C25H32N7O18P3	(H+)-1	811.1017	810.0944	810.0943	-0.06	-1	17.91	0.92
w12:a7-B	C54H68N20O35P6	(H+)-2	1742.2582	870.1218	870.1217	-0.13	-2	9.84	0.91
w12:b5	C39H51N15O25P4	(H+)-2	1253.2131	625.5993	625.5993	0.04	-2	9.24	0.91
w5:a11	C30H37N12O17P3	(H+)-1	930.1612	929.1540	929.1542	0.23	-1	1.12	0.91
w4:a12	C30H37N12O17P3	(H+)-1	930.1612	929.1540	929.1542	0.23	-1	1.12	0.91
w11:a6-B	C34H44N10O24P4	(H+)-1	1100.1480	1099.1408	1099.1406	-0.19	-1	11.21	0.91
w9:a8-B	C34H44N10O24P4	(H+)-1	1100.1480	1099.1408	1099.1406	-0.19	-1	11.21	0.91
w11:a9-B	C62H80N21O42P7	(H+)-3	2007.2933	668.0905	668.0905	0.00	-3	3.17	0.91
w9	C87H112N33O54P9	(H+)-3	2761.4671	919.4818	919.4816	-0.12	-3	4.46	0.90
a10	C97H121N38O56P9	(H+)-3	2992.5427	996.5070	996.5066	-0.38	-3	2.45	0.90
w12:a3	C19H24N8O11P2	(H+)-1	602.1040	601.0967	601.0967	-0.05	-1	2.67	0.90
w6:a9	C19H24N8O11P2	(H+)-1	602.1040	601.0967	601.0967	-0.05	-1	2.67	0.90
w7:a10-B	C33H43N11O22P4	(H+)-2	1069.1535	533.5695	533.5694	-0.16	-2	2.91	0.90
w7:a12-B	C53H68N18O34P6	(H+)-2	1686.2571	842.1213	842.1213	0.07	-2	15.73	0.89
w11	C106H136N41O66P11	(H+)-4	3379.5660	843.8842	843.8839	-0.40	-4	1.92	0.88
a8	C77H97N28O46P7	(H+)-2	2366.4275	1182.2065	1182.2045	-1.71	-2	1.59	0.88
w11:a12-B	C92H117N33O59P10	(H+)-3	2937.4546	978.1442	978.1434	-0.88	-3	1.48	0.88
w12:a8-B	C63H80N23O41P7	(H+)-3	2031.3045	676.0942	676.0942	-0.08	-3	1.77	0.88
w10:a10-B	C63H80N23O41P7	(H+)-3	2031.3045	676.0942	676.0942	-0.08	-3	1.77	0.88
a3	C29H36N10O15P2	(H+)-1	826.1837	825.1764	825.1761	-0.32	-1	1.82	0.87
w12:b5	C39H51N15O25P4	(H+)-1	1253.2131	1252.2058	1252.2078	1.54	-1	1.63	0.87
w8:a9-B	C33H43N11O23P4	(H+)-1	1085.1484	1084.1411	1084.1431	1.82	-1	2.23	0.86
w2	C19H26N8O12P2	(H+)-1	620.1145	619.1073	619.1070	-0.37	-1	8.52	0.86
w12:b3	C19H26N8O12P2	(H+)-1	620.1145	619.1073	619.1070	-0.37	-1	8.52	0.86
w6:b9	C19H26N8O12P2	(H+)-1	620.1145	619.1073	619.1070	-0.37	-1	8.52	0.86
y6:a12-B	C44H55N15O25P4	(H+)-1	1317.2444	1316.2371	1316.2368	-0.24	-1	2.26	0.85
y12:a6-B	C44H55N15O26P4	(H+)-2	1333.2393	665.6124	665.6121	-0.38	-2	3.23	0.85
y12:a6-B	C44H55N15O26P4	(H+)-1	1333.2393	1332.2320	1332.2320	-0.05	-1	2.81	0.85
w9:a9-B	C43H56N13O30P5	(H+)-1	1389.1944	1388.1871	1388.1863	-0.63	-1	3.97	0.85

Type	MF	Ionization	MF Mass	Th m/z	Exp m/z	ppm	Charge	Intensity (%)	Similarity
w7:a9-B	C23H31N6O17P3	(H+)-1	756.0959	755.0886	755.0887	0.17	-1	21.24	0.85
w12:a6-B	C44H56N15O29P5	(H+)-2	1413.2056	705.5955	705.5956	0.13	-2	9.36	0.84
y11:a8-B	C53H67N18O33P5	(H+)-2	1638.2806	818.1330	818.1370	4.86	-2	40.42	0.84
y10:a9-B	C53H67N18O33P5	(H+)-2	1638.2806	818.1330	818.1370	4.86	-2	40.42	0.84
y5:a12-B	C35H43N12O19P3	(H+)-1	1028.1980	1027.1908	1027.1901	-0.66	-1	1.76	0.83
a5	C49H61N17O28P4	(H+)-1	1459.2822	1458.2750	1458.2725	-1.69	-1	1.35	0.83
w10:a12-B	C83H105N30O53P9	(H+)-3	2648.4082	881.7955	881.7951	-0.44	-3	2.07	0.83
y12:a3-B	C15H18N5O7P	(H+)-1	411.0944	410.0871	410.0869	-0.51	-1	0.77	0.82
y5:a10-B	C15H18N5O7P	(H+)-1	411.0944	410.0871	410.0869	-0.51	-1	0.77	0.82
y4:a11-B	C15H18N5O7P	(H+)-1	411.0944	410.0871	410.0869	-0.51	-1	0.77	0.82
w10	C97H124N38O60P10	(H+)-4	3090.5196	771.6226	771.6234	1.05	-4	1.49	0.81
w12:b11	C97H124N38O60P10	(H+)-4	3090.5196	771.6226	771.6234	1.05	-4	1.49	0.81
w11:b12	C97H124N38O60P10	(H+)-4	3090.5196	771.6226	771.6234	1.05	-4	1.49	0.81
a6	C59H73N22O34P5	(H+)-2	1788.3348	893.1601	893.1601	0.01	-2	6.86	0.81
y12:a12-B	C102H128N38O61P10	(H+)-4	3170.5458	791.6292	791.6297	0.63	-4	0.65	0.80
w11:a8-B	C53H68N18O36P6	(H+)-1	1718.2469	1717.2397	1717.2383	-0.80	-1	1.21	0.80
w10:a9-B	C53H68N18O36P6	(H+)-1	1718.2469	1717.2397	1717.2383	-0.80	-1	1.21	0.80
w12	C116H148N46O71P12	(H+)-5	3692.6236	737.5174	737.5175	0.03	-5	0.70	0.80
w12:a12-B	C102H129N38O64P11	(H+)-4	3250.5122	811.6208	811.6217	1.14	-4	0.96	0.80
w8:a10-B	C43H55N16O28P5	(H+)-1	1398.2060	1397.1987	1397.2054	4.79	-1	3.08	0.79
y9:a12-B	C73H92N25O44P7	(H+)-2	2239.3893	1118.6874	1118.6863	-0.96	-2	1.71	0.79
w9:a12-B	C73H93N25O47P8	(H+)-3	2319.3557	772.1113	772.1113	0.04	-3	2.83	0.79
w7:a8	C18H24N6O12P2	(H+)-2	578.0927	288.0391	288.0392	0.47	-2	2.07	0.78
w10	C97H124N38O60P10	(H+)-3	3090.5196	1029.1659	1029.1652	-0.66	-3	0.43	0.77
w12:b11	C97H124N38O60P10	(H+)-3	3090.5196	1029.1659	1029.1652	-0.66	-3	0.43	0.77
w11:b12	C97H124N38O60P10	(H+)-3	3090.5196	1029.1659	1029.1652	-0.66	-3	0.43	0.77
w7:a12-B	C53H68N18O34P6	(H+)-1	1686.2571	1685.2498	1685.2510	0.70	-1	1.78	0.77
w11:b4	C19H26N8O13P2	(H+)-1	636.1095	635.1022	635.1003	-2.97	-1	6.15	0.76
w8:b7	C19H26N8O13P2	(H+)-1	636.1095	635.1022	635.1003	-2.97	-1	6.15	0.76
w5:a10	C20H24N10O10P2	(H+)-2	626.1152	312.0503	312.0504	0.17	-2	2.77	0.76
y12:a8-B	C63H79N23O38P6	(H+)-2	1951.3382	974.6618	974.6601	-1.77	-2	1.15	0.76
y10:a10-B	C63H79N23O38P6	(H+)-2	1951.3382	974.6618	974.6601	-1.77	-2	1.15	0.76

a)

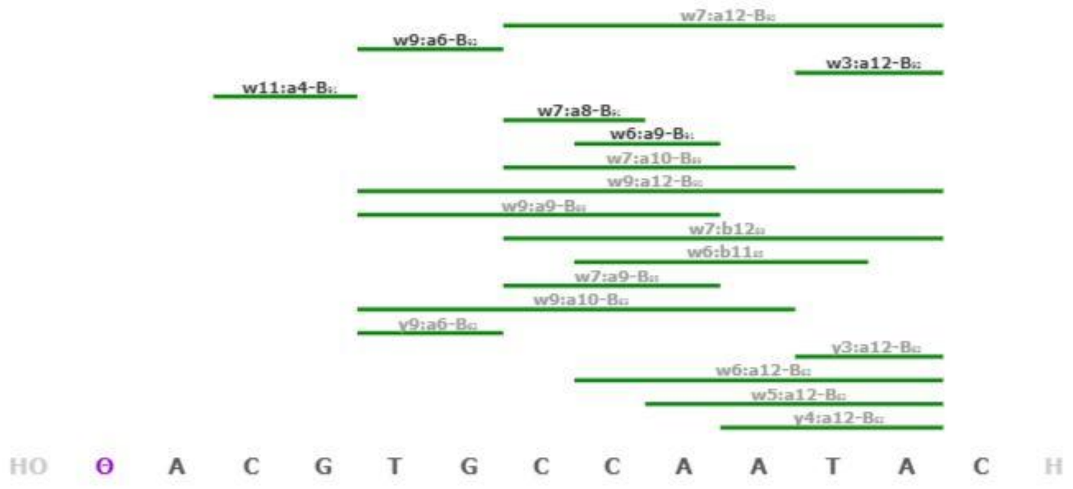

b)

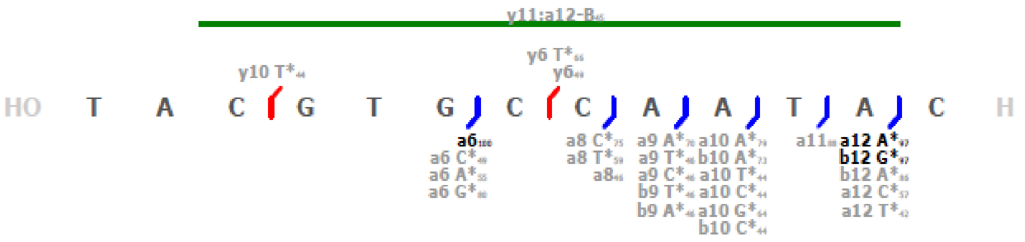

Figure S6. Fragment maps for the platinated S1c strand after CID fragmentation of $[S1c+Pt(NH3)_2-8H]^{6-}$ at *m/z* 689.6103. a) Unplatinated internal fragments. b) Platinated fragments. The maps show identified a-b in blue, y in red and internal fragment ions as green bars. Modified bases are marked in purple. Fragment types marked in black: average similarity > 90%; fragment types marked in gray fragments: average similarity < 90%.

a)

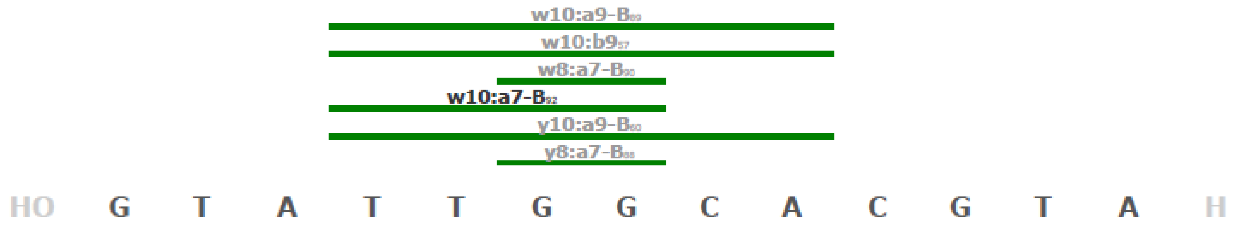

b)

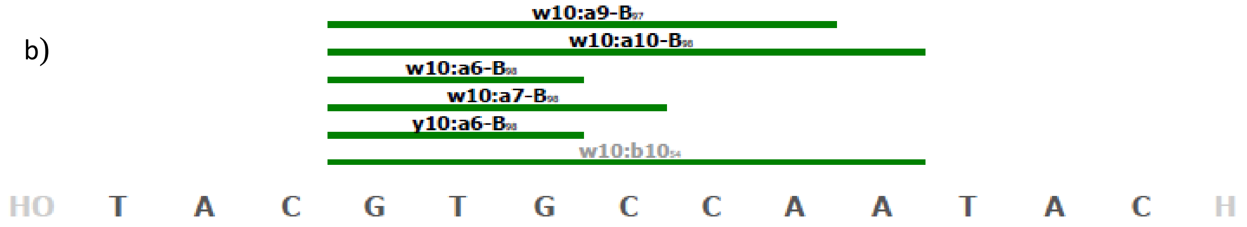

Figure S7. Fragmentation map of the free oligonucleotide S1: (a) the $[S1\text{-}5H]^{5-}$ ion, (b) the $[S1c\text{-}5H]^{5-}$ ion showing internal fragments only.

a)

Sequence of S1c strand 5'-TACGTGCCAATAC-3'

5’end contains TACGTG

3’end contains CCAATAC

Middle Fragments contains CGTGCCAAT

b)

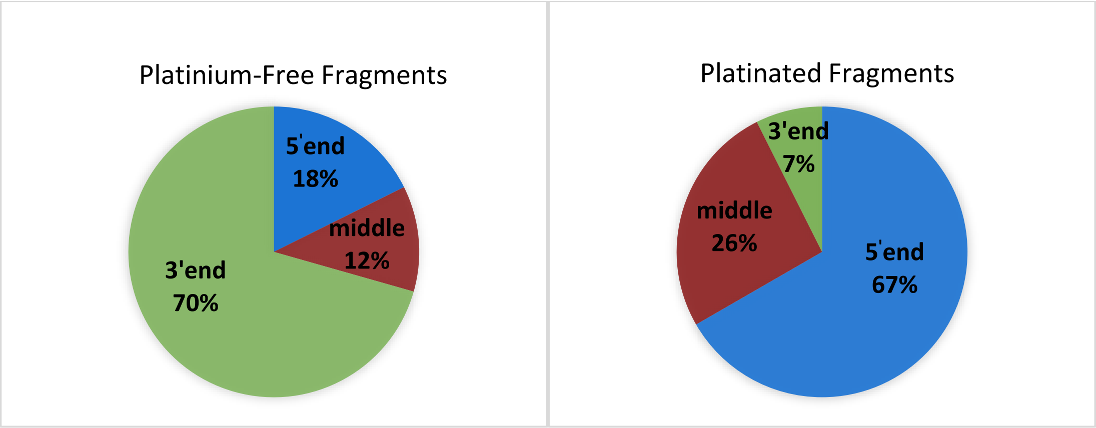

Figure S8. a) Classification of 5'-end (in blue), 3'-end (in green) and Middle fragments (in brown) of S1c strand of double-stranded oligonucleotide. b) Percentage abundance of 5'-end, 3'-end and Middle fragment ions from platinum-free fragmentation and platinated fragment from CID fragmentation of the peak at m/z 689.6103 for the strand S1c.

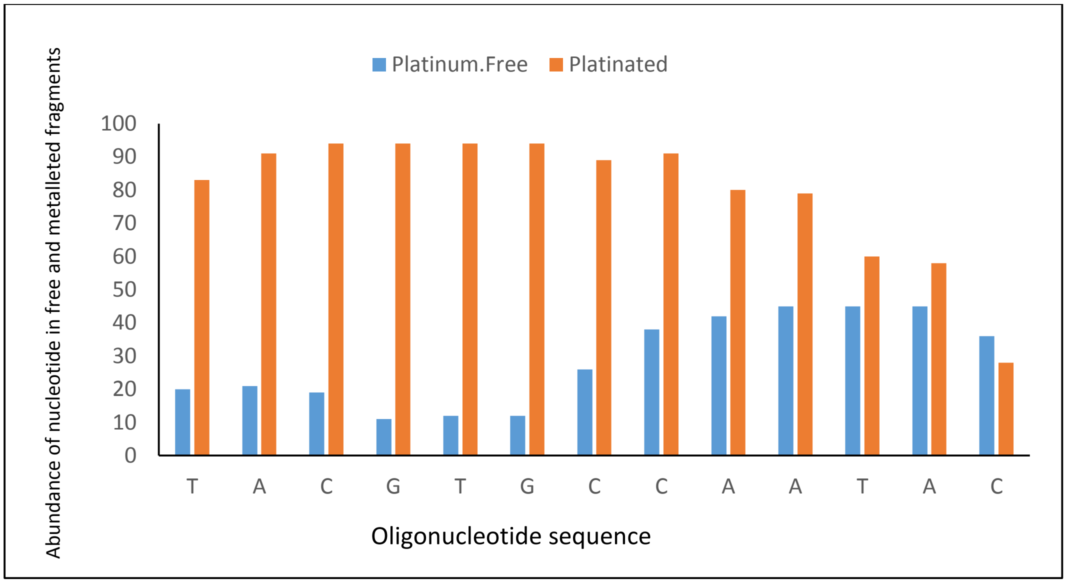

Figure S9. Location of cisplatin binding site in S1c: histogram with the occurrence of nucleotide bases for all assignable platinum-free as well as platinated fragments (all fragments) following CID fragmentation of the peak at m/z 689.6103 for the strand S1c

Supplementary figures and tables for Peptide-Cisplatin studies

Table S8. List of CID fragments from Apm[2]s tool analysis of the parent ion $[P_1+Pt(NH_3)_2]^{2+}$(*m/z*=760.35) including fragment type, molecular formula, ionization and variable groups, MS mass, theoretical and experimental masses with PPM error, charge, intensity and similarity scores in percentage.

Type	MF	Ionization	Variable group	MF Mass	Th m/z	Exp m/z	ppm	Charge	Intensity (%)	Similarity
y9	C49H78N9O18(+1)			1080.5465	1080.5459	1080.5459	-0.04	1	103.25	0.97
a2	C10H24N3OPt(+3)	(H+)-2	Pt(NH3)++	397.1567	395.1405	395.1406	0.16	1	27.92	0.96
y7	C35H62N7O13(+1)			788.4406	788.4400	788.4400	0.00	1	5.67	0.95
a2	C10H21N2OPt(+3)	(H+)-2	Pt++	380.1302	378.1140	378.1140	-0.01	1	2.67	0.94
b5	C31H48N5O8(+1)			618.3503	618.3497	618.3494	-0.51	1	2.99	0.93
b10	C55H92N11O16Pt(+3)	(H+)-1	Pt(NH3)++	1357.6371	678.3141	678.3145	0.58	2	3.37	0.93
b9	C49H78N9O15(+1)			1032.5617	1032.5612	1032.5616	0.42	1	5.87	0.93
y6	C29H51N6O12(+1)			675.3565	675.3559	675.3559	-0.13	1	6.19	0.92
b2	C11H24N3O2Pt(+3)	(H+)-2	Pt(NH3)++	425.1516	423.1354	423.1356	0.46	1	13.45	0.92
b2	C11H27N4O2Pt(+3)	(H+)-2	Pt(NH3)2++	442.1782	440.1620	440.1621	0.26	1	11.03	0.91
y2	C11H21N2O5(+1)			261.1450	261.1445	261.1446	0.23	1	3.22	0.91
y4	C22H39N4O9(+1)			503.2717	503.2712	503.2711	-0.16	1	4.00	0.90
y5	C25H44N5O10(+1)			574.3088	574.3083	574.3081	-0.36	1	7.67	0.88
y8	C44H71N8O15(+1)			951.5039	951.5033	951.5037	0.36	1	3.45	0.88
b10y5	C20H35N4O6(+1)			427.2557	427.2551	427.2550	-0.28	1	0.47	0.87
b4	C25H40N5O7Pt(+3)	(H+)-2	Pt(NH3)++	717.2576	715.2414	715.2416	0.33	1	1.96	0.87
b6	C35H58N7O10Pt(+3)	(H+)-2	Pt(NH3)++	931.3893	929.3731	929.3737	0.67	1	0.97	0.87
b10y3	C12H23N2O2(+1)			227.1760	227.1754	227.1755	0.45	1	0.19	0.87
y3	C17H32N3O6(+1)			374.2291	374.2286	374.2287	0.38	1	2.96	0.86
b9	C49H76N9O14(+1)			1014.5512	1014.5506	1014.5511	0.52	1	0.73	0.86
b6	C35H61N8O10Pt(+3)	(H+)-2	Pt(NH3)2++	948.4159	946.3997	946.4002	0.56	1	2.20	0.86
y8	C44H69N8O14(+1)			933.4933	933.4928	933.4935	0.76	1	4.35	0.86
b9y9	C38H58N7O13(+1)			820.4093	820.4087	820.4091	0.48	1	1.53	0.85
b3	C16H31N4O5Pt(+3)	(H+)-2	Pt(NH3)++	554.1942	552.1780	552.1782	0.33	1	1.21	0.85
b9	C49H81N10O15Pt(+3)	(H+)-2	Pt(NH3)++	1244.5531	1242.5369	1242.5381	1.02	1	3.55	0.84
b9y5	C14H24N3O5(+1)			314.1716	314.1710	314.1715	1.38	1	0.04	0.84
	C60H97N11O20Pt(+2)		Pt++	1486.6559	743.3274	743.3264	-1.35	2	2.53	0.84
b6	C35H55N6O10(+1)			719.3980	719.3974	719.3974	-0.08	1	1.24	0.84
y4	C22H45N6O9Pt(+3)	(H+)-2	Pt(NH3)2++	732.2896	730.2734	730.2719	-2.00	1	0.63	0.83
b9	C49H78N9O15Pt(+3)	(H+)-2	Pt++	1227.5265	1225.5103	1225.5122	1.55	1	0.21	0.83
y6	C29H54N7O12Pt(+3)	(H+)-2	Pt(NH3)++	887.3478	885.3316	885.3318	0.17	1	0.06	0.82
b5	C31H51N6O8Pt(+3)	(H+)-2	Pt(NH3)++	830.3416	828.3254	828.3257	0.38	1	1.55	0.82
b10	C55H95N12O16Pt(+3)	(H+)-1	Pt(NH3)2++	1374.6637	686.8274	686.8272	-0.32	2	2.15	0.82
a10y4	C16H30N3O4(+1)	(H+)-2		328.2236	326.2085	326.2088	0.82	-1	0.08	0.82
b3	C16H28N3O5(+1)			342.2029	342.2023	342.2023	-0.18	1	0.41	0.82
b8	C43H70N9O14Pt(+3)	(H+)-2	Pt(NH3)++	1131.4690	1129.4528	1129.4535	0.65	1	1.66	0.81
b7	C38H63N8O11Pt(+3)	(H+)-2	Pt(NH3)++	1002.4264	1000.4102	1000.4108	0.56	1	0.98	0.81
b10y4	C17H30N3O5(+1)			356.2185	356.2180	356.2182	0.66	1	0.04	0.81
b10	C55H92N11O16Pt(+3)	(H+)-2	Pt(NH3)++	1357.6371	1355.6209	1355.6212	0.22	1	0.88	0.81

Type	MF	Ionization	Variable group	MF Mass	Th m/z	Exp m/z	ppm	Charge	Intensity (%)	Similarity
y2	C11H27N4O5Pt(+3)	(H+)-2	Pt(NH3)2++	490.1629	488.1467	488.1468	0.13	1	2.14	0.81
y2	C11H24N3O5Pt(+3)	(H+)-2	Pt(NH3)++	473.1364	471.1202	471.1201	-0.11	1	1.26	0.80
b8	C43H73N10O14Pt(+3)	(H+)-2	Pt(NH3)2++	1148.4956	1146.4794	1146.4793	-0.08	1	1.29	0.80
b9y6	C18H31N4O7(+1)			415.2193	415.2187	415.2188	0.18	1	0.03	0.80
b7	C38H58N7O10(+1)			772.4245	772.4240	772.4238	-0.20	1	0.36	0.79
a10y5	C19H33N4O4(+1)	(H+)-2		381.2502	379.2351	379.2338	-3.39	-1	0.03	0.79
b4	C25H37N4O7Pt(+3)	(H+)-2	Pt++	700.2310	698.2148	698.2150	0.22	1	0.25	0.79
b7	C38H66N9O11Pt(+3)	(H+)-2	Pt(NH3)2++	1019.4530	1017.4368	1017.4377	0.96	1	1.03	0.79
a8y8	C26H42N7O6Pt(+3)	(H+)-2	Pt(NH3)2++	743.2844	741.2683	741.2700	2.40	1	0.32	0.78
y9	C49H76N9O17(+1)			1062.5359	1062.5354	1062.5355	0.11	1	0.82	0.75
a6y10	C29H42N5O6(+1)	(H+)-2		556.3135	554.2984	554.3004	3.63	-1	0.09	0.74
b10y6	C24H42N5O8(+1)			528.3033	528.3028	528.3024	-0.70	1	0.67	0.74
y3	C17H35N4O6Pt(+3)	(H+)-2	Pt(NH3)++	586.2205	584.2043	584.2043	0.14	1	0.58	0.74
b9	C49H81N10O15Pt(+3)	(H+)-1	Pt(NH3)++	1244.5531	621.7721	621.7718	-0.37	2	0.61	0.74
b4	C25H37N4O7(+1)			505.2662	505.2657	505.2652	-0.95	1	0.23	0.74
y3	C17H38N5O6Pt(+3)	(H+)-2	Pt(NH3)2++	603.2470	601.2308	601.2301	-1.19	1	0.15	0.73
y5	C25H42N5O9(+1)			556.2983	556.2977	556.2978	0.11	1	0.04	0.73
b7	C38H60N7O11(+1)			790.4351	790.4345	790.4341	-0.59	1	0.78	0.73
y5	C25H42N5O9Pt(+3)	(H+)-2	Pt++	751.2630	749.2468	749.2498	3.92	1	0.34	0.72
a2	C10H27N4OPt(+3)	(H+)-2	Pt(NH3)2++	414.1833	412.1671	412.1670	-0.24	1	0.06	0.72
b8y9	C32H47N6O12(+1)			707.3252	707.3246	707.3248	0.21	1	0.63	0.72
a9y9	C37H52N7O9(+1)			738.3827	738.3821	738.3788	-4.52	1	0.25	0.72
b9	C49H84N11O15Pt(+3)	(H+)-2	Pt(NH3)2++	1261.5796	1259.5634	1259.5648	1.05	1	0.85	0.72
y6	C29H47N6O10(+1)			639.3354	639.3348	639.3359	1.72	1	0.04	0.71
b5	C31H54N7O8Pt(+3)	(H+)-2	Pt(NH3)2++	847.3682	845.3520	845.3526	0.72	1	0.19	0.71
b6y9	C24H37N6O6Pt(+3)	(H+)-2	Pt(NH3)2++	700.2422	698.2261	698.2258	-0.37	1	0.11	0.71

a)

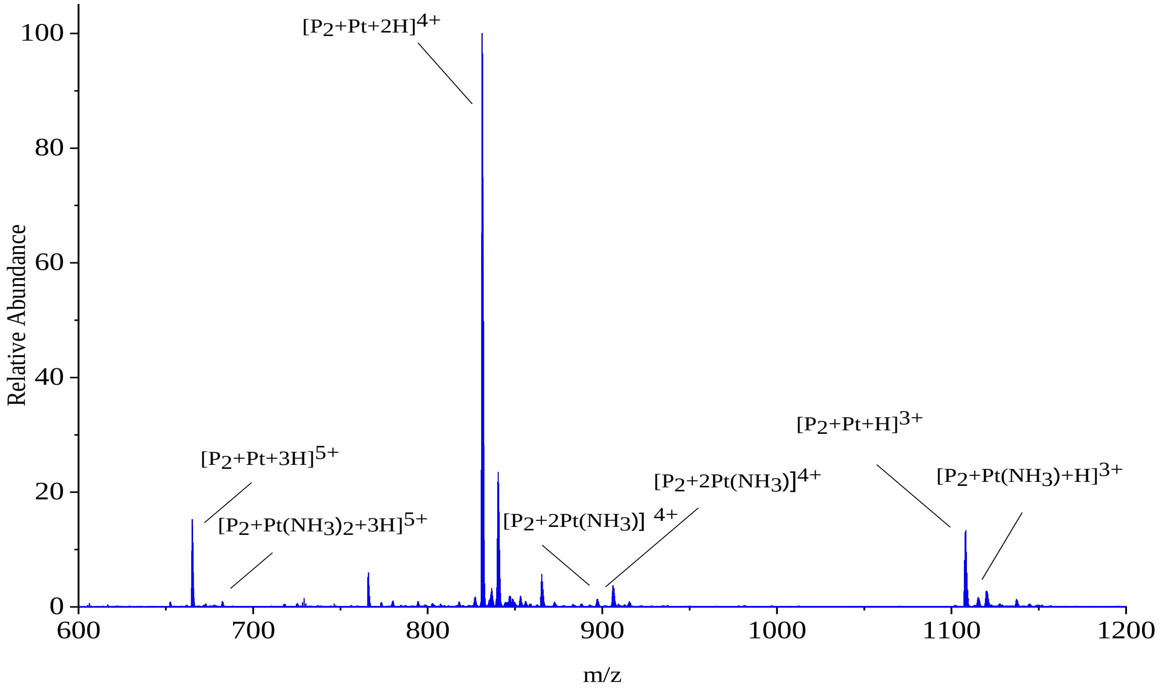

[P2+Pt+2H]4+
[P2+Pt+3H]5+
[P2+Pt(NH3)2+3H]5+
[P2+2Pt(NH3)] 4+
[P2+2Pt(NH3)]4+
[P2+Pt+H]3+
[P2+Pt(NH3)+H]3+
Relative Abundance
100
80
60
40
20
0
600
700
800
900
1000
1100
1200
m/z

b)

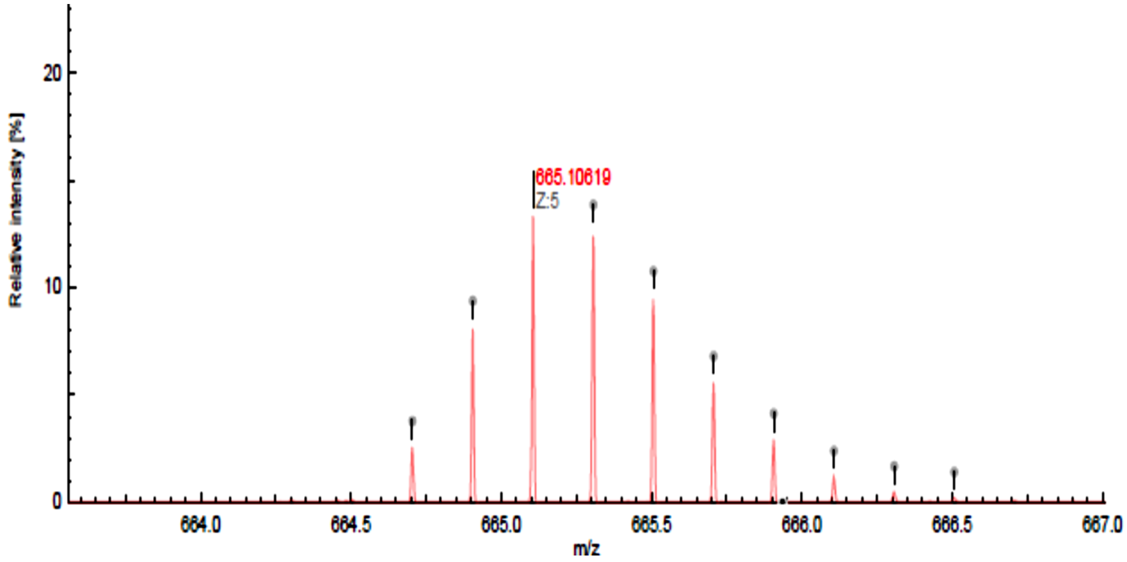

665.10619
Z:5
Relative Intensity [%]
20
10
0
664.0
664.5
665.0
665.5
666.0
666.5
667.0
m/z

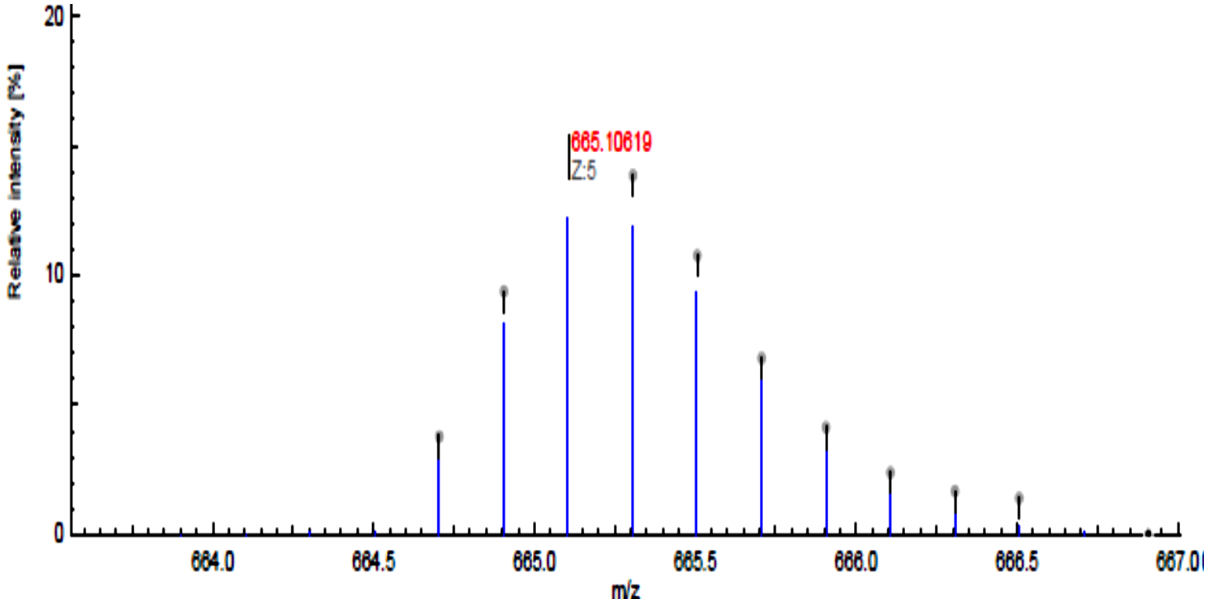

Figure S10. a) Full scan mass spectra of P_2:Cisplatin (1:2) incubated at 37°C for 48 h in the range *m/z* 600-1200. b) Comparison of experimental (top) and theoretical (bottom) isotopic patterns for $[P_2+Pt+3H]^{5+}$ ion, as reported by Apm[2]s.

Table S9. List of CID fragments from Apm[2]s tool analysis of the parent ion $[P_2+Pt+3H]^{5+}$ (*m/z*= *m/z*=664.9087) including fragment type, molecular formula, ionization and variable groups, MS mass, theoretical and experimental masses with ppm error, charge, intensity and similarity scores in percentage. Neutral losses ions are not included.

Type	MF	Ionization	Variable group	MF Mass	Th m/z	Exp m/z	ppm	Charge	Intensity (%)	Similarity
y21	C107H162N31O33(+1)	(H+)2		2409.1951	803.7364	803.7363	-0.06	3	77.61	0.99
y15	C78H122N23O22(+1)	(H+)2		1732.9135	578.3092	578.3090	-0.35	3	65.33	0.99
y19	C100H151N28O29(+1)	(H+)2		2208.1202	736.7114	736.7113	-0.13	3	65.43	0.99
y20	C103H156N29O31(+1)	(H+)2		2295.1522	765.7221	765.7221	-0.01	3	28.64	0.98
y18	C91H142N27O28(+1)	(H+)2		2061.0518	687.6886	687.6884	-0.28	3	94.96	0.98
y24	C120H185N34O36S(+1)	(H+)2		2710.3411	904.1184	904.1185	0.08	3	5.47	0.98
b21	C101H160N27O31PtS2(+3)		Pt++	2506.0863	835.3615	835.3617	0.20	3	6.11	0.98
a24y25	C100H160N29O30PtS2(+3)		Pt++	2506.0975	835.3653	835.3617	-4.28	3	6.11	0.97
y14	C72H111N22O21(+1)	(H+)		1619.8294	810.4181	810.4182	0.13	2	16.21	0.97
y26	C128H199N36O38PtS2(+3)	(H+)	Pt++	3107.3835	777.0973	777.0974	0.08	4	5.04	0.97
b26	C122H198N35O37PtS2(+3)		Pt++	3004.3777	1001.4587	1001.4585	-0.19	3	8.14	0.97
y7	C36H57N12O10(+1)	(H+)		817.4321	409.2194	409.2197	0.80	2	12.09	0.96
b26y19	C85H133N24O25(+1)	(H+)		1889.9874	945.4970	945.4976	0.57	2	5.21	0.96
y22	C112H171N32O34S(+1)	(H+)2		2540.2356	847.4165	847.4165	-0.02	3	8.19	0.96
y17	C86H133N26O27(+1)	(H+)2		1961.9833	654.6658	654.6656	-0.22	3	81.35	0.96
b27	C128H205N38O38PtS2(+3)	(H+)	Pt++	3141.4366	785.6106	785.6107	0.20	4	25.53	0.96
y17	C86H133N26O27(+1)	(H+)		1961.9833	981.4950	981.4951	0.05	2	13.90	0.96
b26	C122H198N35O37PtS2(+3)	(H+)	Pt++	3004.3777	751.3458	751.3457	-0.16	4	5.37	0.95
b17	C85H134N23O25PtS2(+3)		Pt++	2135.9011	711.9665	711.9666	0.25	3	0.82	0.95
y18	C91H142N27O28(+1)	(H+)		2061.0518	1031.0292	1031.0293	0.01	2	7.35	0.95
y15	C78H122N23O22(+1)	(H+)		1732.9135	866.9601	866.9602	0.08	2	10.41	0.95
a27	C127H205N38O37PtS2(+3)	(H+)	Pt++	3113.4417	778.6118	778.6119	0.11	4	4.04	0.95
b27y17	C77H122N25O24(+1)	(H+)		1780.9095	890.9581	890.9579	-0.17	2	4.85	0.95
b26y17	C71H115N22O23(+1)	(H+)		1643.8505	822.4286	822.4290	0.41	2	6.96	0.95
y16	C82H127N24O25(+1)	(H+)2		1847.9404	616.6515	616.6513	-0.26	3	17.83	0.94
b27	C128H205N38O38PtS2(+3)	(H+)2	Pt++	3141.4366	628.6899	628.6896	-0.42	5	5.92	0.94
y16	C82H127N24O25(+1)	(H+)		1847.9404	924.4736	924.4736	0.00	2	6.41	0.94
y14	C72H111N22O21(+1)	(H+)2		1619.8294	540.6145	540.6142	-0.42	3	11.34	0.94
b27y17	C77H122N25O24(+1)	(H+)2		1780.9095	594.3078	594.3083	0.77	3	3.69	0.93
b27y7	C27H46N11O7(+1)	(H+)		636.3582	318.6824	318.6829	1.51	2	0.77	0.93
b27y19	C91H140N27O26(+1)	(H+)2		2027.0463	676.3534	676.3532	-0.29	3	3.56	0.93
y18	C91H142N27O28(+1)	(H+)3		2061.0518	516.0183	516.0181	-0.22	4	1.24	0.93
b3	C12H23N4O4(+1)			287.1719	287.1714	287.1720	2.09	1	0.17	0.93
b26y21	C92H144N27O29(+1)	(H+)		2091.0623	1046.0345	1046.0344	-0.15	2	5.66	0.93
b26y18	C76H124N23O24(+1)	(H+)		1742.9190	871.9628	871.9630	0.22	2	8.23	0.92
b24	C113H182N33O35PtS2(+3)	(H+)	Pt++	2820.2565	705.3155	705.3153	-0.28	4	0.34	0.92
y19	C100H151N28O29(+1)	(H+)		2208.1202	1104.5635	1104.5639	0.39	2	3.22	0.92
y9	C43H67N14O14(+1)	(H+)		1003.4961	502.2514	502.2516	0.34	2	3.91	0.92
b27y21	C98H151N30O30(+1)	(H+)2		2228.1212	743.3784	743.3784	-0.01	3	3.10	0.91
y25	C125H194N35O37PtS2(+3)	(H+)	Pt++	3036.3464	759.3380	759.3380	-0.03	4	2.68	0.91
b25y20	C85H133N24O26(+1)	(H+)		1905.9823	953.4945	953.4964	1.98	2	0.19	0.91
b17y21	C55H80N15O17(+1)	(H+)		1222.5857	611.7962	611.7966	0.73	2	0.34	0.91
y13	C63H102N21O20(+1)	(H+)2		1472.7610	491.5917	491.5919	0.37	3	0.92	0.91
y12	C58H95N20O17(+1)	(H+)		1343.7184	672.3626	672.3630	0.71	2	2.66	0.91

Type	MF	Ionization	Variable group	MF Mass	Th m/z	Exp m/z	ppm	Charge	Intensity (%)	Similarity
y19	C100H151N28O29(+1)	(H+)3		2208.1202	552.7854	552.7854	0.00	4	2.06	0.91
b26y24	C105H167N30O32S(+1)	(H+)		2392.2083	1196.6075	1196.6083	0.63	2	0.50	0.91
b27y18	C82H131N26O25(+1)	(H+)		1879.9779	940.4923	940.4922	-0.07	2	4.29	0.91
y12	C58H95N20O17(+1)	(H+)2		1343.7184	448.5775	448.5776	0.26	3	3.55	0.91
a27	C127H205N38O37PtS2(+3)	(H+)2	Pt++	3113.4417	623.0909	623.0906	-0.59	5	1.24	0.91
y22	C112H171N32O34S(+1)	(H+)3		2540.2356	635.8142	635.8140	-0.42	4	1.80	0.91
b27y18	C82H131N26O25(+1)	(H+)2		1879.9779	627.3306	627.3306	-0.09	3	5.21	0.90
b12y18	C9H16N3O3(+1)			214.1192	214.1186	214.1192	2.62	1	0.49	0.90
b27y20	C94H145N28O28(+1)	(H+)2		2114.0783	705.3641	705.3640	-0.13	3	1.47	0.90
b27y19	C91H140N27O26(+1)	(H+)		2027.0463	1014.0265	1014.0265	0.03	2	1.10	0.90
b13y17	C8H12N3O5(+1)			230.0777	230.0771	230.0777	2.48	1	0.14	0.90
y9	C43H67N14O14(+1)			1003.4961	1003.4956	1003.4955	-0.06	1	5.67	0.89
b27y14	C63H100N21O18(+1)	(H+)		1438.7555	719.8811	719.8813	0.26	2	2.50	0.89
b2	C9H18N3O3(+1)			216.1348	216.1343	216.1348	2.38	1	0.87	0.89
b26y14	C57H93N18O17(+1)	(H+)		1301.6966	651.3517	651.3519	0.39	2	0.83	0.89
b27y15	C69H111N22O19(+1)	(H+)2		1551.8396	517.9512	517.9509	-0.55	3	2.40	0.89
b27y24	C111H174N33O33S(+1)	(H+)2		2529.2673	843.7604	843.7613	1.10	3	0.66	0.89
y10	C46H72N15O15(+1)	(H+)		1074.5332	537.7700	537.7699	-0.14	2	2.90	0.89
y2	C15H19N4O4(+1)			319.1406	319.1401	319.1405	1.22	1	4.85	0.88
b27y15	C69H111N22O19(+1)	(H+)		1551.8396	776.4232	776.4234	0.33	2	3.73	0.88
b25	C119H193N34O36PtS2(+3)	(H+)	Pt++	2933.3406	733.5866	733.5866	0.08	4	0.37	0.88
a27y7	C26H46N11O6(+1)	(H+)		608.3633	304.6850	304.6855	1.55	2	0.40	0.88
b22y10	C13H21N4O6(+1)			329.1461	329.1456	329.1460	1.47	1	0.98	0.88
a10y20	C11H15N2O2(+1)			207.1134	207.1128	207.1133	2.17	1	0.21	0.88
b22y9	C10H16N3O5(+1)			258.1090	258.1084	258.1093	3.18	1	0.03	0.88
b25y19	C82H128N23O24(+1)	(H+)		1818.9503	909.9785	909.9784	-0.08	2	0.32	0.87
b27y20	C94H145N28O28(+1)	(H+)		2114.0783	1057.5425	1057.5433	0.77	2	0.35	0.87
b27y26	C119H188N35O35PtS2(+3)		Pt++	2926.3096	975.4360	975.4378	1.86	3	0.34	0.87
b19y14	C29H45N8O7(+1)			617.3411	617.3406	617.3401	-0.76	1	0.38	0.87
b26y20	C88H138N25O27(+1)	(H+)		1977.0194	989.0131	989.0134	0.32	2	1.42	0.87
b4y26	C8H15N2O2S(+1)			203.0854	203.0849	203.0855	2.88	1	0.05	0.86
a11y19	C13H19N2O(+1)			219.1497	219.1492	219.1497	2.42	1	0.66	0.86
b14y17	C14H23N4O6(+1)			343.1618	343.1612	343.1616	1.16	1	0.85	0.85
y21	C107H162N31O33(+1)	(H+)3		2409.1951	603.0541	603.0535	-1.00	4	0.20	0.85
b10y20	C12H15N2O3(+1)			235.1083	235.1077	235.1081	1.81	1	0.17	0.85
b27y12	C49H84N19O14(+1)	(H+)		1162.6445	581.8256	581.8260	0.62	2	0.32	0.85
b24y18	C67H108N21O22(+1)	(H+)		1558.7978	779.9023	779.9026	0.44	2	0.29	0.85
b27y22	C103H160N31O31S(+1)	(H+)2		2359.1617	787.0586	787.0587	0.16	3	0.80	0.85
a15y15	C14H21N2O(+1)			233.1654	233.1648	233.1653	2.00	1	0.03	0.85
b11y19	C14H19N2O2(+1)			247.1447	247.1441	247.1448	2.99	1	0.24	0.85
b8y22	C9H16N3O3S(+1)			246.0912	246.0907	246.0912	2.06	1	0.36	0.85
b26y15	C63H104N19O18(+1)	(H+)		1414.7807	707.8937	707.8937	0.06	2	3.41	0.84
b27y21	C98H151N30O30(+1)	(H+)		2228.1212	1114.5640	1114.5645	0.44	2	1.78	0.84
y27	C134H211N38O39PtS2(+3)	(H+)2	Pt++	3235.4785	647.4983	647.4968	-2.29	5	0.61	0.84

Type	MF	Ionization	Variable group	MF Mass	Th m/z	Exp m/z	ppm	Charge	Intensity (%)	Similarity
b9y24	C20H35N6O7S(+1)			503.2288	503.2282	503.2283	0.16	1	0.63	0.84
b10y21	C16H21N4O5(+1)			349.1512	349.1506	349.1512	1.51	1	2.52	0.84
b27y13	C54H91N20O17(+1)	(H+)		1291.6871	646.3469	646.3467	-0.38	2	0.27	0.83
b22y15	C45H71N12O13(+1)	(H+)		987.5264	494.2665	494.2662	-0.78	2	0.17	0.82
y10	C46H72N15O15(+1)			1074.5332	1074.5327	1074.5337	0.90	1	1.77	0.82
y24	C120H185N34O36S(+1)	(H+)3		2710.3411	678.3406	678.3419	1.93	4	0.11	0.82
a27y21	C97H151N30O29(+1)	(H+)2		2200.1263	734.0468	734.0468	0.08	3	0.29	0.81
b11y20	C17H24N3O4(+1)			334.1767	334.1761	334.1770	2.60	1	0.03	0.81
a15y24	C56H84N13O15S(+1)	(H+)		1210.5931	605.7999	605.8019	3.31	2	0.09	0.81
b24y13	C39H68N15O14(+1)	(H+)		970.5070	485.7569	485.7572	0.74	2	0.05	0.81
b9y22	C12H21N4O5S(+1)			333.1233	333.1227	333.1235	2.22	1	0.17	0.81
b27y16	C73H116N23O22(+1)	(H+)		1666.8665	833.9366	833.9365	-0.15	2	1.38	0.81
b7y24	C13H24N3O3S(+1)			302.1538	302.1533	302.1537	1.38	1	0.10	0.81
b18y14	C26H40N7O6(+1)			546.3040	546.3035	546.3031	-0.71	1	0.27	0.81
b27y14	C63H100N21O18(+1)	(H+)2		1438.7555	480.2565	480.2564	-0.26	3	0.14	0.80
y4	C24H35N6O6(+1)	(H+)		503.2618	252.1343	252.1348	2.04	2	0.04	0.80
y6	C33H52N11O9(+1)	(H+)		746.3949	373.7008	373.7013	1.20	2	0.18	0.80
b18y13	C17H31N6O5(+1)	(H+)		399.2356	200.1212	200.1221	4.93	2	0.03	0.80
b25y21	C89H139N26O28(+1)	(H+)		2020.0252	1010.5160	1010.5162	0.21	2	0.45	0.80
b8y24	C17H30N5O5S(+1)			416.1968	416.1962	416.1966	0.84	1	1.01	0.80

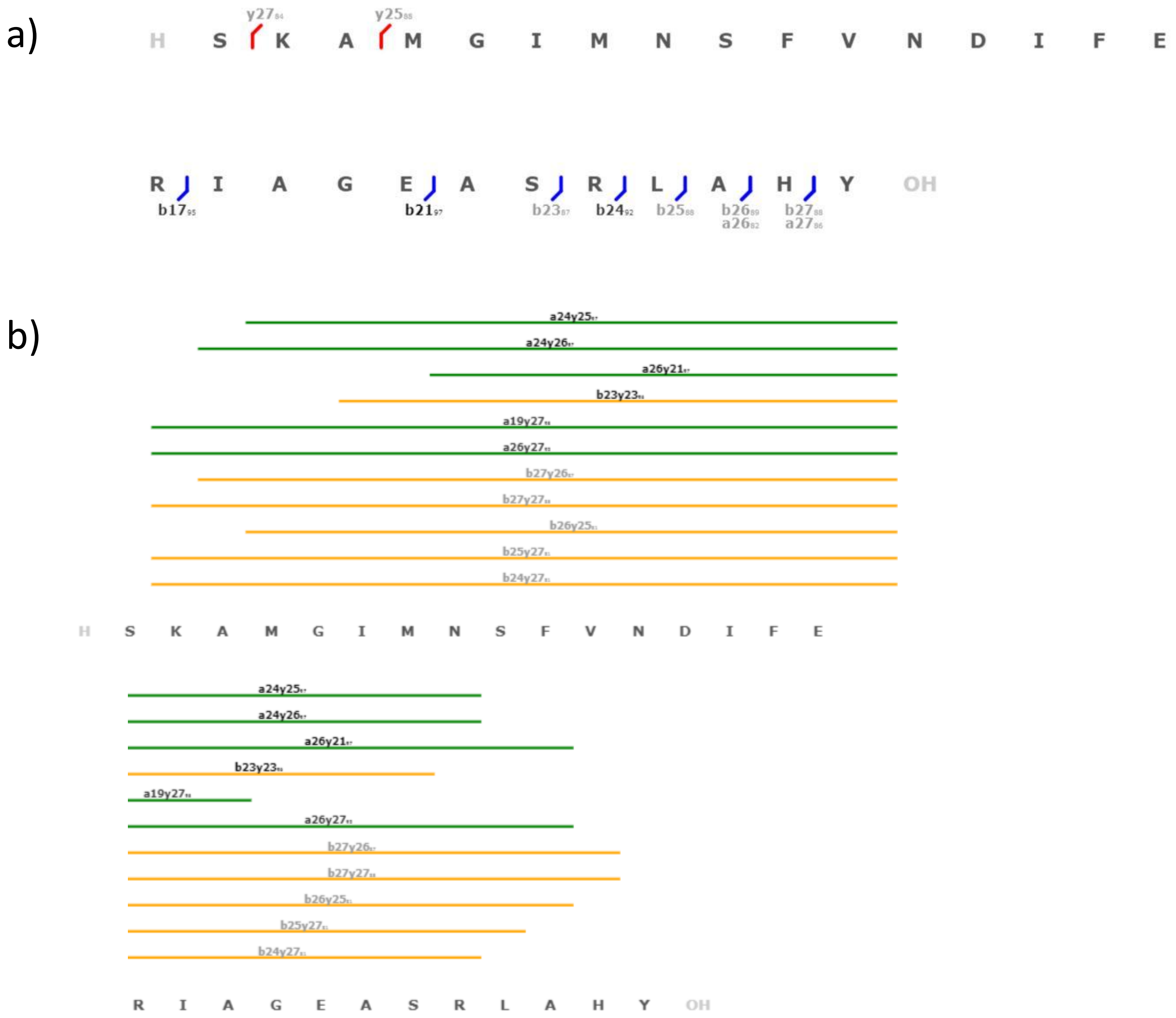

Figure S11. Visual map for CID experiment of the $[P_2+Pt+3H]^{5+}$ ion containing (a) b/y type platinated fragments, (b) Internal-platinated fragment.

Supplementary figure for competitive interaction of cisplatin with P_1 and oligonucleotide

a)

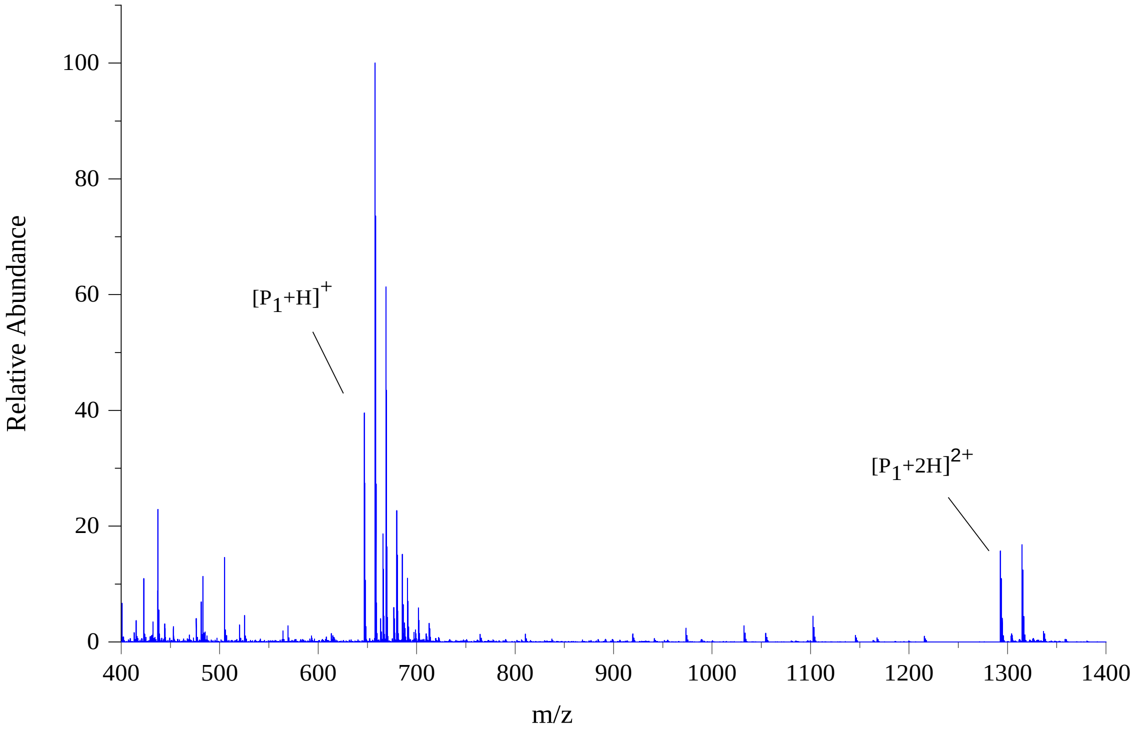

b)

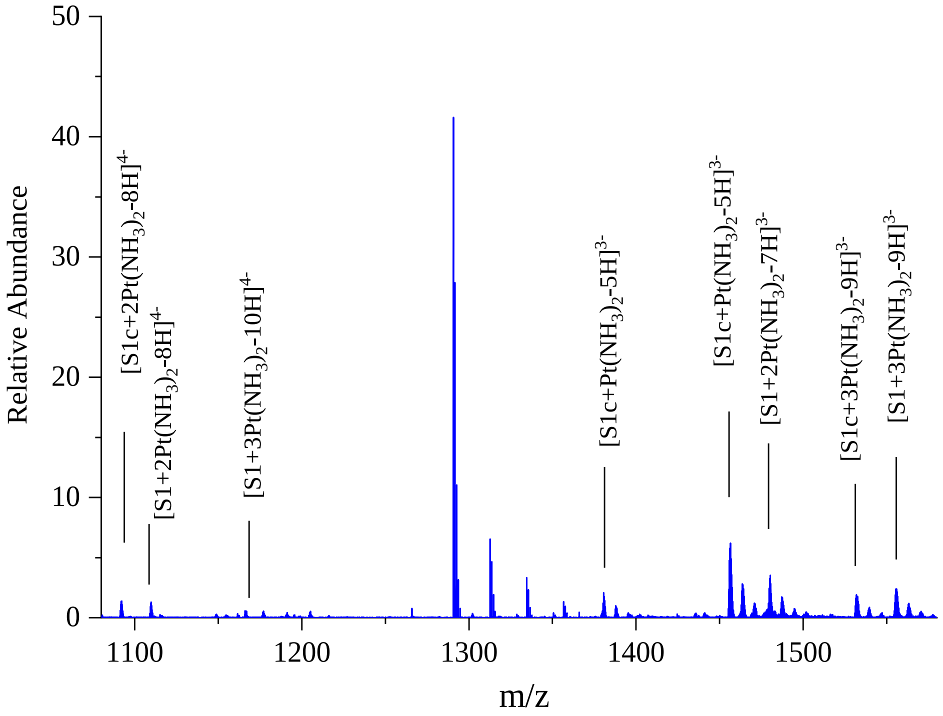

Figure S12. Spectra obtained following 50h of incubation of cisplatin with P_1 and oligonucleotide (a) Positive ion polarity (b) Negative ion polarity.

Supplementary figure for competitive interaction of cisplatin with P_2 and oligonucleotide

Table S10. List of adducts (free and platinated) obtained in negative ion mode using the Aom^{2}s /Apm^{2}s tool for incubation of peptide P2 / Oligonucleotide / cisplatin in a ratio of (1:1:3) for: a) 24 and b) 50 h. The table includes the MF, the ionization type, mass, theoretical and experimental m/z with ppm error, charge, intensity and similarity scores in percentage.

a)

MF	Ionization	MF Mass	Th m/z	Exp m/z	ppm	Charge	Intensity	Similarity
C126H166N50O75P12Pt(+2)	(H+)-5	4145.7212	1380.2279	1380.2357	5.68	-3	1.64	0.94
C126H160N48O75P12	(H+)-3	3916.7033	1304.5605	1304.5637	2.45	-3	0.03	0.84
C128H167N51O77P12Pt(+2)	(H+)-5	4216.7219	1403.8948	1403.9013	4.61	-3	0.04	0.77
C126H178N54O75P12Pt3(+6)	(H+)-9	4603.7570	1531.5627	1531.5703	4.96	-3	0.22	0.75

b)

MF	Ionization	MF Mass	Th m/z	Exp m/z	ppm	Charge	Intensity	Similarity
C128H173N53O77P12Pt2(+4)	(H+)-7	4445.7398	1479.5622	1479.5608	-0.94	-3	0.91	0.99
C126H172N52O75P12Pt2(+4)	(H+)-7	4374.7391	1455.8953	1455.8935	-1.22	-3	1.32	0.98
C126H166N50O75P12Pt(+2)	(H+)-5	4145.7212	1380.2279	1380.2259	-1.44	-3	1.21	0.97
C128H179N55O77P12Pt3(+6)	(H+)-9	4674.7577	1555.2296	1555.2283	-0.83	-3	0.44	0.95
C126H178N54O75P12Pt3(+6)	(H+)-9	4603.7570	1531.5627	1531.5628	0.03	-3	0.07	0.85
C126H165N49O76P12Pt(+2)	(H+)-5	4146.7052	1380.5559	1380.5600	2.96	-3	1.96	0.81
C128H167N51O77P12Pt(+2)	(H+)-5	4216.7219	1403.8948	1403.9007	4.22	-3	0.04	0.78

a)

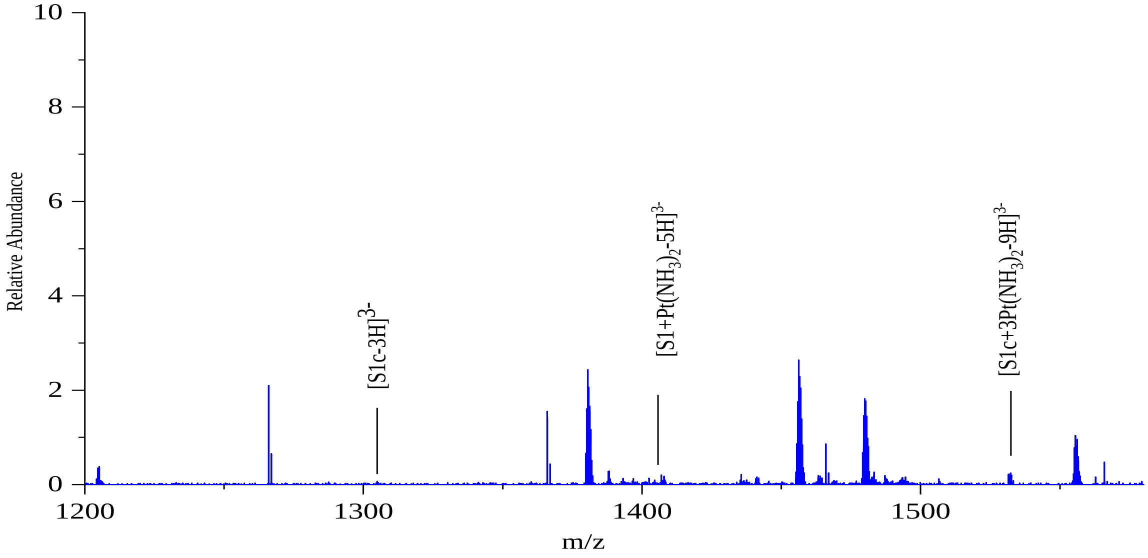

b)

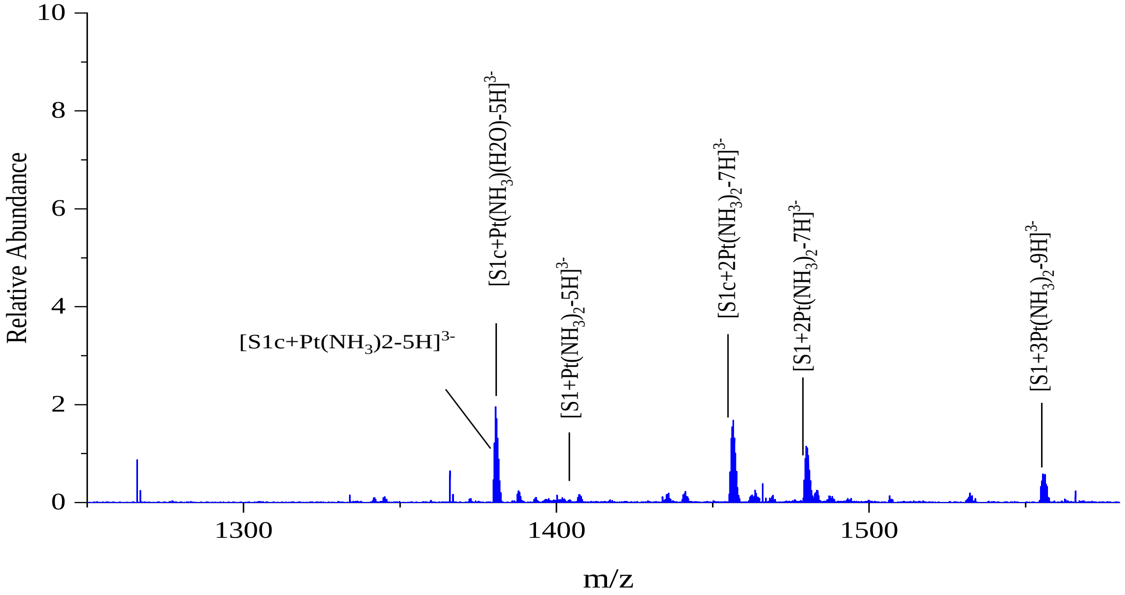

Figure S13. Negative ion spectra obtained following incubation of cisplatin with the P_2 and oligonucleotide for: (a) 24 h and (b) 50 h.

Supplementary figures for transfer studies

Purification of Platinated P_2

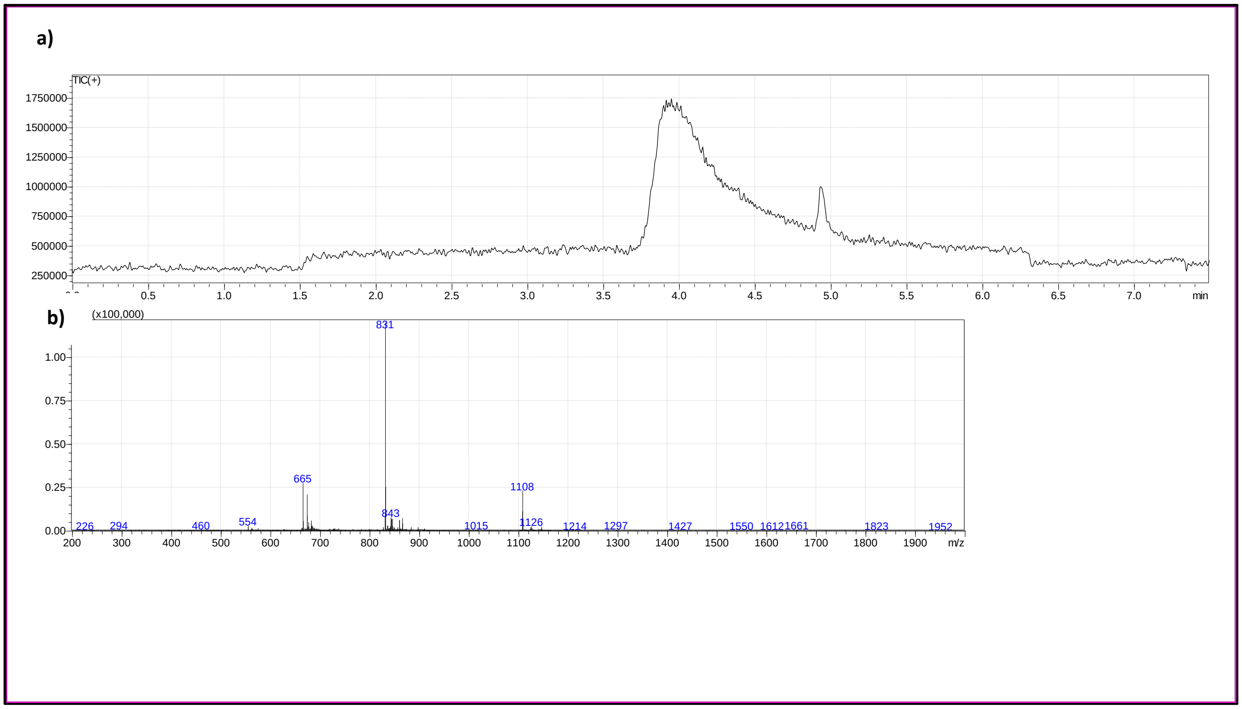

Figure S14. a) Purification of platinated peptide P_2. A) Total Ion Chromatogram obtained in positive ion mode b) Mass spectrum obtained after purification of platinated P_2 showing the main ions at *m/z* 664.90 (z=5), *m/z* 830.8 (z=4), and *m/z* 1107.5 (z=3).

Table S11. List of adducts obtained in positive ion mode using the Apm^{2}s tool for filtered sample of peptide P_2 / cisplatin incubated in a ratio of (1:3) for 48 h. The table includes the MF, the ionization type, mass, theoretical and experimental m/z with ppm error, charge, intensity and similarity scores in percentage.

MF	Ionization	MF Mass	Th m/z	Exp m/z	ppm	Charge	Intensity	Similarity
C137H215N39O41PtS2(+2)	(H+)2	3321.503	830.879	830.876	-3.71	4	16.15	0.99
C137H215N39O41PtS2(+2)	(H+)	3321.503	1107.503	1107.499	-3.35	3	34.21	0.99
C137H215N39O41PtS2(+2)		3321.503	1660.751	1660.745	-3.66	2	1.87	0.96
C137H215N39O41PtS2(+2)	(H+)3	3321.503	664.9047	664.9016	-4.68	5	0.87	0.93
C137H221N41O41Pt2S2(+4)		3550.521	887.6296	887.6258	-4.33	4	0.18	0.92
C137H221N41O41Pt2S2(+4)	(H+)	3550.521	710.3051	710.3029	-3.18	5	0.04	0.72

Table S12. List of adducts obtained in positive ion mode using the Apm^{2}s tool for HPLC purified platinated peptide P_2 from filtered sample of peptide P_2 / cisplatin incubated in a ratio of (1:3) for 48 h. The table includes the MF, the ionization type, mass, theoretical and experimental m/z with ppm error, charge, intensity and similarity scores in percentage.

MF	Ionization	MF Mass	Th m/z	Exp m/z	ppm	Charge	Intensity	Similarity
C137H215N39O41PtS2(+2)	(H+)2	3321.503	830.879	830.8789	-0.14	4	67.34	0.99
C137H215N39O41PtS2(+2)	(H+)	3321.503	1107.503	1107.503	-0.31	3	45.67	0.97
C137H215N39O41PtS2(+2)		3321.503	1660.751	1660.749	-1.27	2	1.03	0.94
C137H221N41O41Pt2S2(+4)		3550.521	887.6296	887.6281	-1.71	4	0.7	0.92
C137H221N41O41Pt2S2(+4)	(H+)	3550.521	710.3051	710.3037	-1.99	5	0.12	0.88

Table S13. Transfer studies. List of adducts (free and platinated) obtained in negative ion mode using the Aom[2]s tool for incubation of platinated peptide P_2 with Oligonucleotide (1:1) for: a) 12h and b) 24h. The table includes the MF, the ionization type, mass, theoretical and experimental m/z with ppm error, charge, and intensity and similarity scores in percentage.

a)

MF	Ionization	MF Mass	Th m/z	Exp m/z	ppm	Charge	Intensity	Similarity
C128H161K2N49O77P12Pt(+4)	(H+)-7	4260.5962	1417.8477	1417.8423	-3.82	-3.00	0.22	0.81
C128H161K2N49NaO77P12Pt(+5)	(H+)-8	4283.5860	1425.1750	1425.1731	-1.31	-3.00	0.03	0.78

b)

MF	Ionization	MF Mass	Th m/z	Exp m/z	ppm	Charge	Intensity	Similarity
C126H160N48O75P12	(H+)-3	3916.7033	1304.5605	1304.5604	-0.06	-3	12.09	0.98
C126H160N48NaO75P12(+1)	(H+)-4	3939.6931	1311.8878	1311.8873	-0.35	-3	4.03	0.96
C128H161N49O77P12	(H+)-3	3987.7040	1328.2274	1328.2278	0.27	-3	2.31	0.95
C128H161K2N49O77P12Pt(+4)	(H+)-7	4260.5962	1417.8477	1417.8404	-5.13	-3	1.22	0.90
C128H161N49NaO77P12(+1)	(H+)-4	4010.6938	1335.5547	1335.5562	1.09	-3	1.27	0.87
C126H160N48O75P12	(H+)-4	3916.7033	978.1685	978.1717	3.24	-4	0.25	0.81
C128H161K2N49NaO77P12Pt(+5)	(H+)-8	4283.5860	1425.1750	1425.1681	-4.85	-3	0.22	0.80
C126H160KN48NaO75P12(+2)	(H+)-5	3978.6568	1324.5398	1324.5350	-3.61	-3	3.05	0.77
C128H161K2N49NaO77P12(+3)	(H+)-6	4088.6212	1360.8586	1360.8636	3.65	-3	0.26	0.77

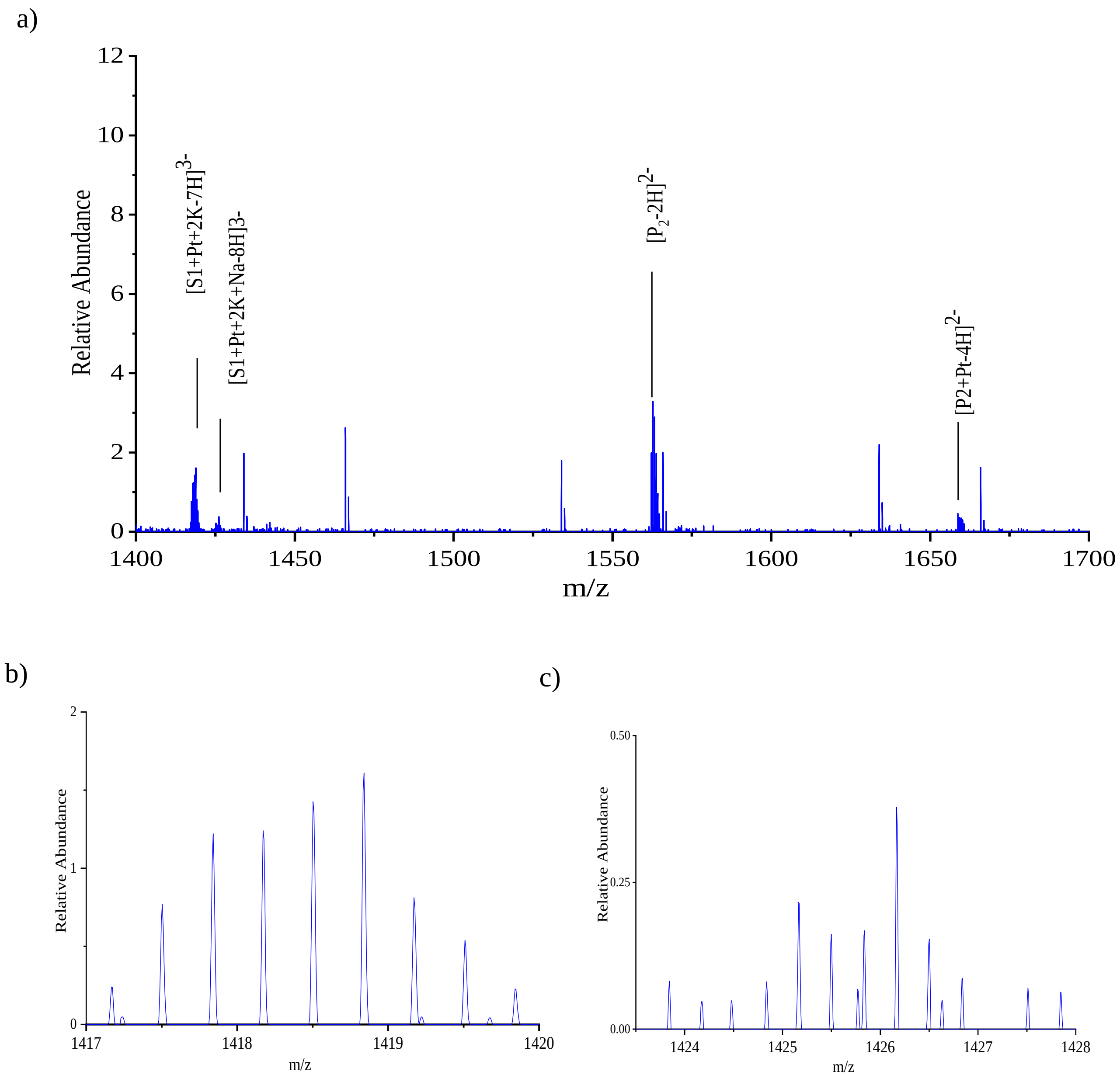

Figure S15. Transfer studies. a) Full scan 1400-1700 m/z mass spectra of 1:1 platinated P_2/oligonucleotide incubated for 24 h at 37°C. Zoom on the *m/z* of platinated oligonucleotide: b) [S1+Pt+2K-7H]$^{3-}$ at *m/z* 1417.8477 (similarity 90%). c) [S1+Pt+2K+Na-8H]$^{3-}$ at *m/z* 1425.1750 (similarity 80%).

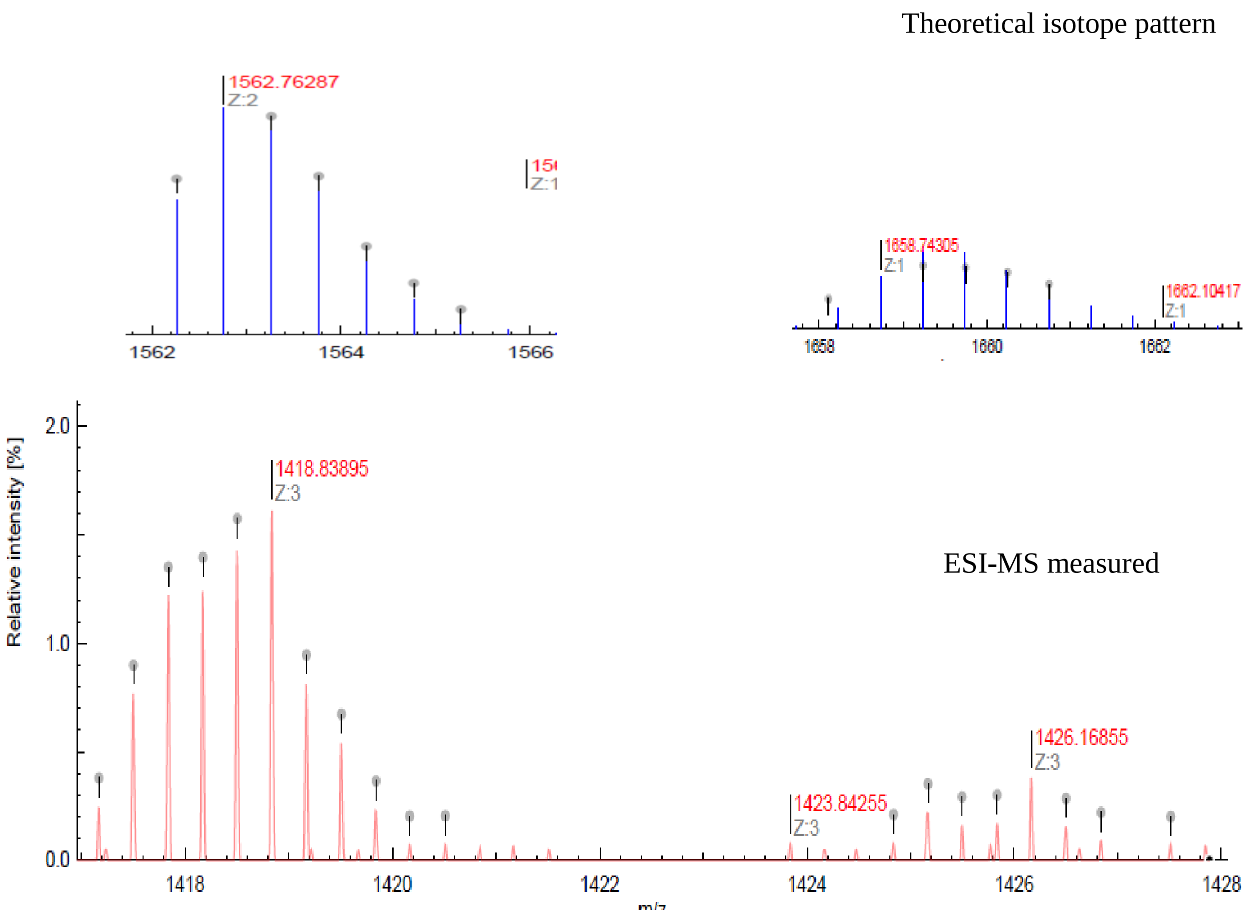

Figure S16. Transfer studies. Negative ion HR mass spectrum. Zoom on the *m/z* of two platinated oligonucleotide, $[S1+Pt+2K-7H]^{3-}$ at *m/z* 1417.8477 (similarity 90%) and $[S1+Pt+2K+Na-8H]^{3-}$ at *m/z* 1425.1750 (similarity 80%) obtained following 24h incubation of the platinated P_2 and oligonucleotide (1:1). The theoretical isotopic patterns are given for comparison.

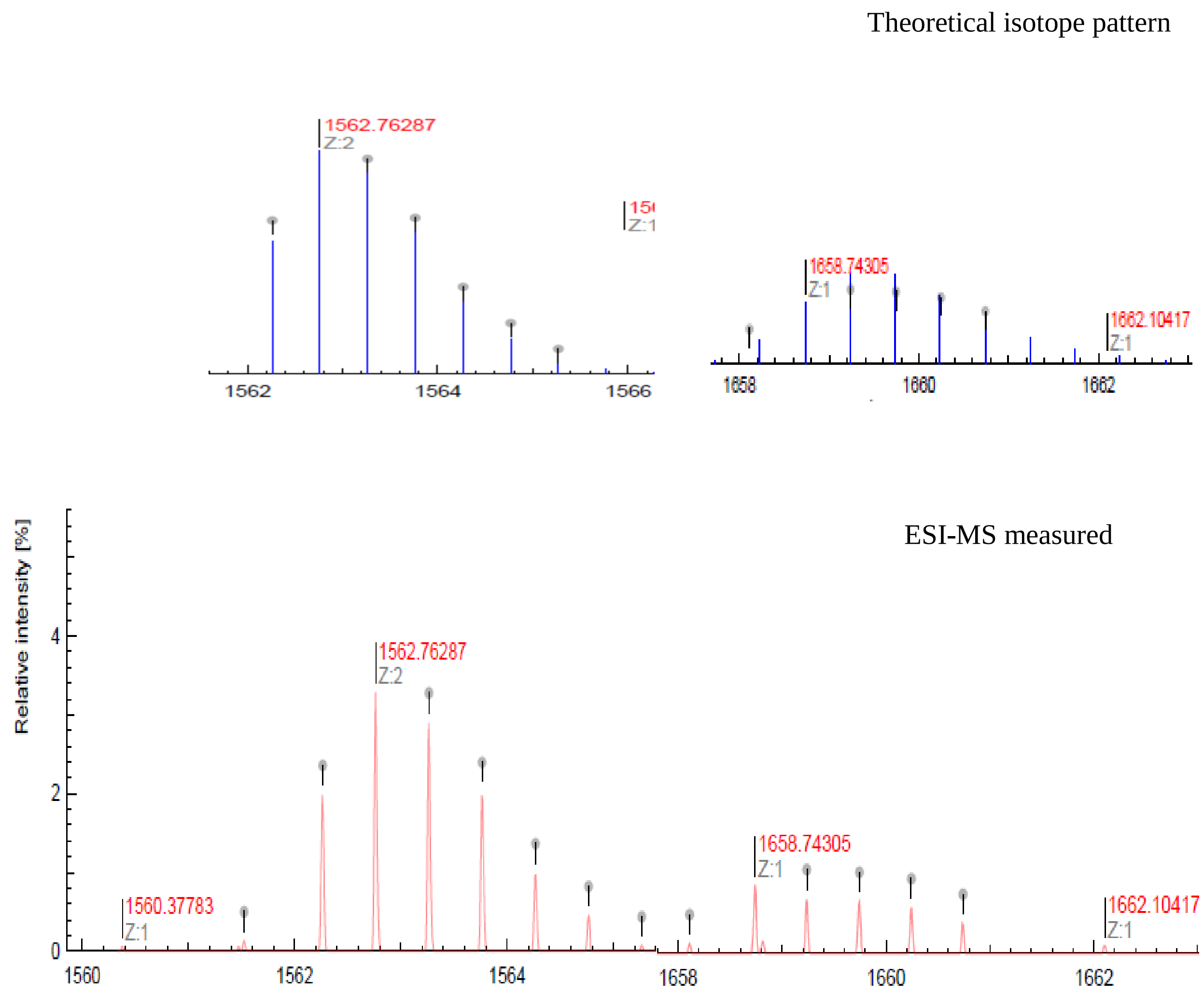

Figure S17. Transfer studies. Negative ion HR mass spectrum. Zoom on the *m/z* ion of free P2 ion species $[P_2\text{-}2H]^{2-}$ at *m/z* 1562.2617 (similarity 96%) and Platinated P_2, $[P_2\text{+}Pt\text{-}4H]^{2-}$ at *m/z* 1658.7430 (similarity 83%) obtained following 24h incubation of the platinated P_2 and oligonucleotide (1:1). The theoretical isotopic patterns are given for comparison.

References:

[1] D. Ortiz, N. Gasilova, F. Sepulveda, L. Patiny, P. J. Dyson, and L. Menin, “Aom2S: A new web-based application for DNA/RNA tandem mass spectrometry data interpretation,” *Rapid Commun. Mass Spectrom.*, vol. 34, no. 23, p. e8927, 2020.

[2] L. Patiny and A. Borel, “ChemCalc: A Building Block for Tomorrow’s Chemical Infrastructure,” *J. Chem. Inf. Model.*, vol. 53, no. 5, pp. 1223–1228, May 2013.

[3] S. A. McLuckey, G. J. Van Berkel, and G. L. Glish, “Tandem mass spectrometry of small, multiply charged oligonucleotides.,” *J. Am. Soc. Mass Spectrom.*, vol. 3, no. 1, pp. 60–70, Jan. 1992.

[4] R. F. S. Lee, L. Menin, L. Patiny, D. Ortiz, and P. J. Dyson, “Versatile Tool for the Analysis of Metal–Protein Interactions Reveals the Promiscuity of Metallodrug–Protein Interactions,” *Anal. Chem.*, vol. 89, no. 22, pp. 11985–11989, 2017.
